# Artificial Intelligence for Myocardial Infarction Detection via Electrocardiogram: A Scoping Review

**DOI:** 10.3390/jcm14196792

**Published:** 2025-09-25

**Authors:** Sosana Bdir, Mennatallah Jaber, Osaid Tanbouz, Fathi Milhem, Iyas Sarhan, Mohammad Bdair, Thaer Alhroob, Walaa Abu Alya, Mohammad Qneibi

**Affiliations:** 1 Department of Medicine, Faculty of Medicine and Allied Health Sciences, An-Najah National University, Nablus P400, Palestine; s12027767@stu.najah.edu (S.B.); jaberminna@gmail.com (M.J.); osaidbtanbouz@gmail.com (O.T.); iyas.sarhan03@gmail.com (I.S.); mohammad.bdair02@gmail.com (M.B.); 2 Neuro-Pal Center, Ramallah P627, Palestine; fathimelhem2021@gmail.com; 3 Department of Internal Medicine, University of Toledo Medical Center, Toledo, OH 43614, USA; thairhroub1710@gmail.com; 4 Department of Internal Medicine, Cleveland Clinic Mercy Hospital, Canton, OH 44708, USA; 5 Department of Biomedical Sciences and Basic Clinical Skills, Faculty of Medicine and Allied Health Sciences, An-Najah National University, Nablus P400, Palestine

**Keywords:** artificial intelligence, myocardial infarction, 12-lead ECG, CNN, detection

## Abstract

**Background/Objectives**: Acute myocardial infarction (MI) is a major cause of death worldwide, and it imposes a heavy burden on health care systems. Although diagnostic methods have improved, detecting the disease early and accurately is still difficult. Recently, AI has demonstrated increasing capability in improving ECG-based MI detection. From this perspective, this scoping review aimed to systematically map and evaluate AI applications for detecting MI through ECG data. **Methods**: A systematic search was performed in Ovid MEDLINE, Ovid Embase, Web of Science Core Collection, and Cochrane Central. The search covered publications from 2015 to 9 October 2024; non-English articles were included if a reliable translation was available. Studies that used AI to diagnose MI via ECG were eligible, and studies that used other diagnostic modalities were excluded. The review was performed per the PRISMA extension for scoping reviews (PRISMA-ScR) to ensure transparent and methodological reporting. Of a total of 7189 articles, 220 were selected for inclusion. Data extraction included parameters such as first author, year, country, AI model type, algorithm, ECG data type, accuracy, and AUC to ensure all relevant information was captured. **Results**: Publications began in 2015 with a peak in 2022. Most studies used 12-lead ECGs; the Physikalisch-Technische Bundesanstalt database and other public and single-center datasets were the most common sources. Convolutional neural networks and support vector machines predominated. While many reports described high apparent performance, these estimates frequently came from relatively small, single-source datasets and validation strategies prone to optimism. Cross-validation was reported in 57% of studies, whereas 36% did not specify their split method, and several noted that accuracy declined under inter-patient or external validation, indicating limited generalizability. Accordingly, headline figures (sometimes ≥99% for accuracy, sensitivity, or specificity) should be interpreted in light of dataset size, case mix, and validation design, with risks of spectrum/selection bias, overfitting, and potential data leakage when patient-level independence is not enforced. **Conclusions**: AI-based approaches for MI detection using ECGs have grown quickly. Diagnostic performance is limited by dataset and validation issues. Variability in reporting, datasets, and validation strategies have been noted, and standardization is needed. Future work should address clinical integration, explainability, and algorithmic fairness for safe and equitable deployment.

## 1. Introduction

Myocardial infarction (MI) or heart attack continues to be among the main causes of death and chronic morbidity globally. It poses a considerable challenge to health systems because it has a high prevalence, imposes heavy economic costs, and necessitates immediate diagnosis and treatment [1,2]. The World Health Organization (WHO) states that millions of cases of MI are documented annually around the world, and this number keeps increasing as a result of aging populations, dietary habits, physical inactivity, and stress [3]. Early detection and accurate diagnosis of MI continue to be challenging despite tremendous developments in clinical treatment and medical technologies, particularly in prehospital triage, overcrowded urban emergency departments, and rural or low-resource hospitals without on-site interventional cardiology or 24/7 expert ECG readers [4,5,6,7].

Among the fundamental diagnostic tools used in managing MI is the electrocardiogram (ECG), which gives real-time information about the heart’s electrical activity. It is extensively used as it is readily available, rapid, and does not invade any anatomical structure [8,9]. The interpretation of an ECG needs clinical acumen and can be limited by atypical presentations, subtle ECG changes, and individual variation in analysis [10]. This can be associated with misdiagnosis, delayed treatment, and worse outcomes in emergencies. This indicates the need for more effective, standardized, precise procedures to interpret ECG information [11].

Artificial intelligence (AI) has quickly become an innovative solution in health care, particularly optimizing diagnosis procedures [12]. Recent analyses of comprehensive AI diagnostic platforms, such as Microsoft’s AI Diagnostic Orchestrator (MAI-DxO), demonstrate their potential to streamline workflows, reduce costs, and enhance clinical decision support [13]. AI’s capability to process big datasets and identify complicated, non-linear trends is now widely used to interpret ECGs in cases of MI [14,15]. Machine and deep learning algorithms, especially Convolutional Neural Networks (CNN), can spot minute and vital ECG features that can be possible signs of the early stages of MI [16]. Such technologies provide uniform, high-speed analysis that can aid doctors in making prompt and accurate decisions [17]. Recent advancements have revealed that AI can enhance ECG diagnosis by identifying trends like ST-segment elevations, T-wave abnormalities, and other electrocardiographic markers of acute injury to the myocardium [18]. Beyond acute detection, AI algorithms can also estimate the risk of future cardiac events by combining ECG information with patient demographics and clinical and laboratory data [19]. Technology such as the MI3 model and risk stratification algorithms assist clinicians in personalizing patient treatment and triaging high-risk patients earlier [20].

Advanced applications like AI-Based Alarm Strategies take it to an additional level by integrating ECG outcomes with clinical signs and troponin values to maximize triage and minimize treatment delays [21]. Such models have emerged as effective in minimizing critical parameters such as “door-to-balloon” time, which is crucial in enhancing outcomes for patients who receive emergency percutaneous coronary intervention. AI further assists medical practitioners in high-volume settings by automating elements of diagnosis to ensure that no hidden clues go unobserved [22,23].

Even with these advancements, there are challenges. The current literature base of AI-aided ECG interpretation continues to be fragmented, with studies differing in data sources, sample sizes, validation methods, and reporting standards, and there is considerable variation in data validity. Furthermore, issues related to algorithm explainability, patient privacy of data, and algorithm biases need to be resolved before widespread clinical application. Compatibility with clinical practice and gaining clinician trust will be equally crucial to successful implementation [24].

In light of the rapidly changing AI technologies and their growing use in cardiology, it is imperative to analyze systematically how these tools influence ECG-based diagnosis of MI. This scoping review will chart the range of AI use in ECG interpretation of MI, discover missing areas in existing literature, and provide input to future studies and practice. By summarizing existing evidence, this review intends to facilitate the development of novel, effective, and equitable diagnostic tools to enhance patient outcomes in MI.

This scoping review systematically maps and evaluates AI methods for MI detection using ECG data by cataloging commonly used model families (e.g., CNN, SVM, artificial neural networks (ANN), random forests) together with input representations and lead configurations; inventorying public versus single-center datasets and whether patient-level independence is enforced; summarizing validation designs (random or intra-patient versus inter-patient splits, internal versus external validation, cross-validation practices); synthesizing reported performance metrics (accuracy, sensitivity, specificity, AUROC, F1, where reported) with attention to case mix and sample size; and identifying gaps and risks of bias, including spectrum/selection bias, overfitting, and data leakage, as well as the current state of clinical integration, explainability, and algorithmic fairness. Methods and reporting follow the PRISMA extension for Scoping Reviews (PRISMA-ScR).

## 2. Materials and Methods

We conducted a scoping review to systematically map research on AI applications for MI detection using ECG data. Methods followed the Joanna Briggs Institute (JBI) guidance for scoping reviews. No formal protocol was registered for this scoping review; however, the review was conducted following the PRISMA-ScR guidelines.

### 2.1. Data Sources and Searches

The search strategy was developed in collaboration with a medical librarian (T.K.) and structured around the Population–Concept–Context (PCC) framework. The Population included patients or human ECG datasets, the concept encompassed artificial intelligence methods (including machine learning, deep learning, and neural networks) for MI detection, and the context included healthcare settings. Controlled vocabulary terms were used alongside keywords to capture relevant studies. Terms included “Artificial Intelligence”, “Myocardial Infarction”, “Electrocardiography”, “Diagnosis”, “Forecasting”, and “Diagnostic Imaging”, as well as their relevant synonyms and keyword variations to maximize search sensitivity across all databases. Boolean operators, truncation, and proximity operators were applied to maximize sensitivity. The search was executed in Ovid MEDLINE, Ovid Embase, Web of Science Core Collection, and Cochrane Central. We systematically searched the literature covering the last 10 years, from 1 January 2015, to 9 October 2024. Non-English studies were included only if a reliable translation was available; otherwise, they were excluded. No publication type restrictions were used in the search strategy.

### 2.2. Eligibility Criteria

We included original studies that used AI methods (e.g., machine learning, deep learning) to detect or diagnose MI using ECG data. Studies whose primary diagnostic input was not ECG (e.g., echocardiography, biomarkers, imaging without ECG) were excluded. (Operational definitions used in searching and screening treated “ECG” and “ECG” as equivalent and “myocardial infarction,” “acute coronary syndrome,” and “ischaemia/infarction” as within the MI diagnostic spectrum, with final inclusion limited to studies explicitly addressing MI detection.)

### 2.3. Study Selection and Data Extraction

We used Covidence software (https://www.covidence.org/, accessed on 15 October 2024) for screening and data extraction. Duplicates were removed both by the software and manually. Two researchers [I.S. and F.M.] independently conducted the title and abstract screening, while two others [S.B. and M.J.] conducted the full-text screening. W.A. resolved conflicts.

The final studies included were exported as an Excel sheet. For each study, we extracted key parameters including first author, year of publication, country, AI model type, algorithm used, ECG data type, and primary performance metrics such as accuracy and area under the receiver operating characteristic curve (AUC). Additional data were also collected to provide comprehensive information on study characteristics and methodology, including data source, types of AI models, common algorithms, data preprocessing techniques, ECG signal features used for AI model training, additional patient data included, cross-validation approaches, outcome specificity, sensitivity, F1-score, and reported clinical outcomes. Data extraction was performed by [W.A. and T.H.], with any discrepancies resolved through discussion.

### 2.4. Synthesis of Results

Given the objectives of a scoping review, we used descriptive, narrative synthesis. We mapped publication trends over time and by country; summarized ECG input types, datasets, and algorithm families; and tabulated model-building/validation practices and performance metrics. The text and figures present findings as counts/percentages, and detailed per-study data are provided in Table S1. Methods for scoping synthesis (mapping and frequency summaries rather than meta-analysis) align with JBI guidance.

### 2.5. Critical Appraisal of Individual Sources

Consistent with scoping reviews to map and characterize evidence, we did not undertake formal risk-of-bias or quality appraisal.

## 3. Results

**Figure 1:**
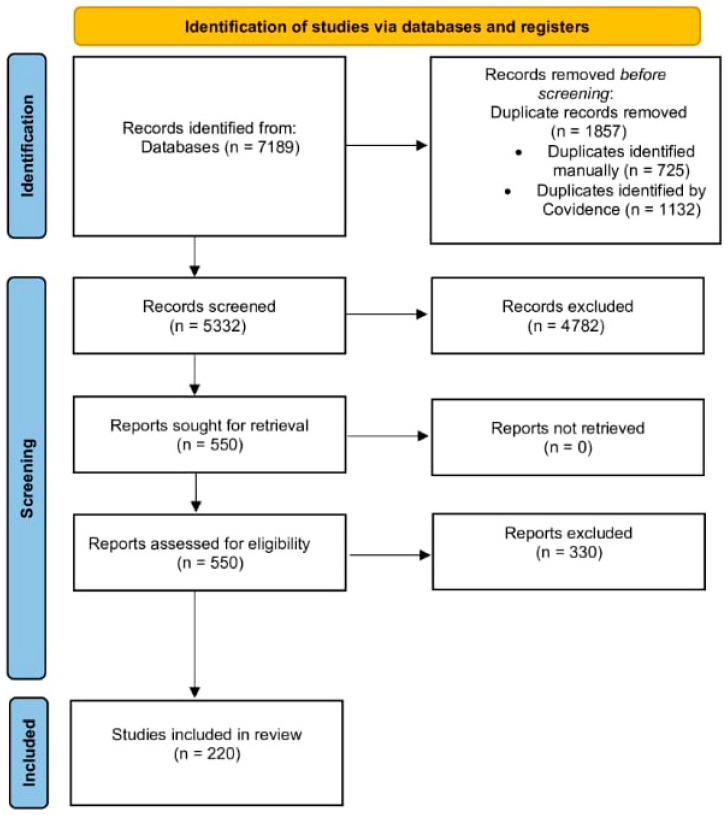
PRISMA Flow Diagram of Study Selection Process.

Our research strategy yielded 7189 articles, of which Covidence identified 1132 as duplicates, while 726 were identified manually. Only two-hundred-and-twenty articles were included in the final data extraction, as shown in the PRISMA flow diagram (Figure 1, Table S1).

**Figure 2:**
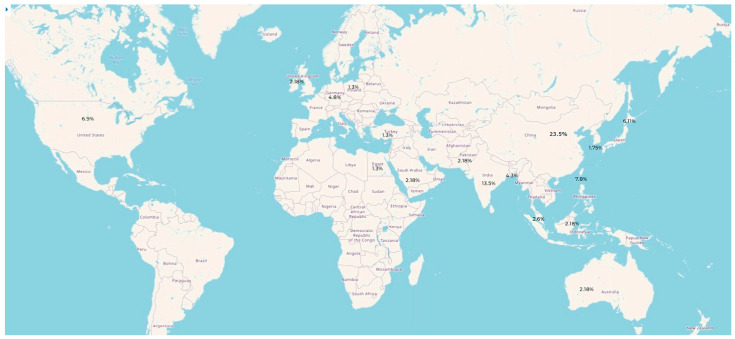
Global Distribution of Publications on AI-Based Detection of MI.

Publications on the use of AI in detecting MI have increased steadily over recent years, reaching a peak in 2022, reflecting growing interest in AI-based ECG detection of myocardial infarction. China contributed the largest number of publications, while 17% were conducted across multiple countries. The number of publications per country is shown in Figure 2.

**Figure 3:**
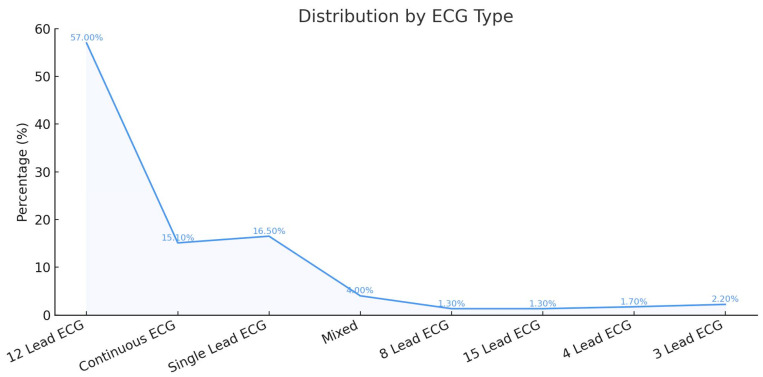
Frequency of ECG Lead Types Used in AI-Based Detection of MI.

Most studies used 12-lead ECG in their AI training and testing, followed by single-lead ECG, most commonly lead II. Continuous ECG was used in 15% of the studies and was sometimes combined with other ECG leads in 4%. Figure 3 shows the frequency of the ECG leads used.

### 3.1. Data Validation Approaches for AI-Based ECG Models

In order to evaluate AI models, data validation methods were performed, including cross-validation (CV), a common method in which the dataset is divided into training and validation sets. The included studies used CV in 57% of the studies, while 36% of the publications did not specify the splitting technique [25]. In addition, inter-patient validation was used to assess model generalizability across entirely unseen patients. Several studies that reported ≥ 99% accuracy on random or intra-patient splits showed lower performance under stricter inter-patient or external evaluation; for example, 100% → 95.65%, 99.92% → 95.49%, and 99.81% → 92.69% (−4–8 percentage points). In some localization/domain-shift settings, declines were larger (≈99% → ≈55%).

### 3.2. ECG Data Source

In addition, several studies relied on custom or institution-specific datasets, including those with multi-lead configurations or continuous ECG recordings (e.g., 12-lead SECG and 3-lead OECG at 1000 Hz, or extended 15-lead ECGs incorporating Frank XYZ vector leads).

**Figure 4:**
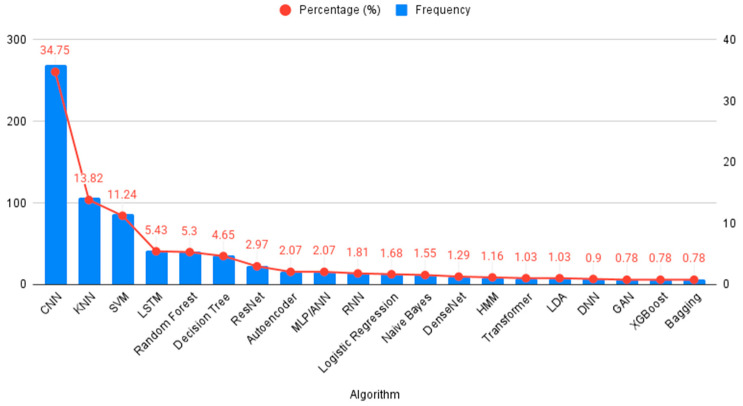
AI Algorithms Used for ECG-Based Detection of MI.

Figure 4 demonstrates the algorithms used in AI training. Some algorithms, such as CNN and SVM, were commonly used across multiple studies, while others, like the Cascade Correlation Neural Network, were used in only a few studies.

### 3.3. Diagnostic Performance

Unsurprisingly, most algorithms demonstrated high accuracy, sensitivity, and specificity, with most studies achieving results between 99% and 100%. Multiple CNN studies reported accuracy above 99%, though these results were often achieved on relatively small datasets and inner-patient validation, which may introduce bias and limit generalizability. Performance tends to be lower with stricter inter-patient validation.

**Figure 5:**
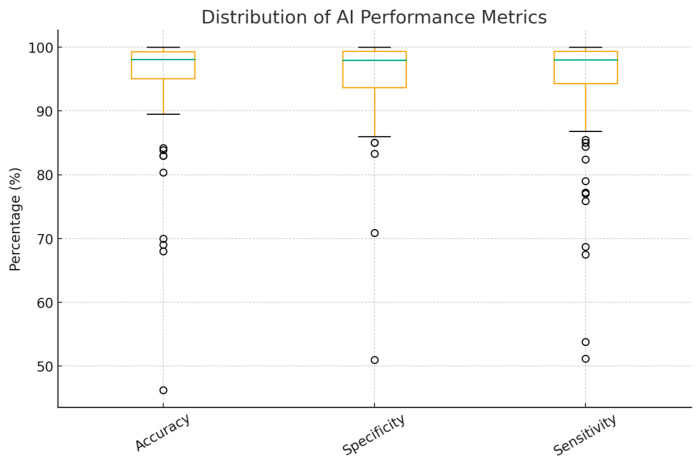
Accuracy, Sensitivity, and Specificity of AI Models Used in MI Detection Studies. Circles represent outliers.

Overall accuracy ranged from 70% to 100%, with the peak performance of 100% observed in many studies, including different models, which are single period with multiple infarction areas, ANN with time-domain HRV parameters, EfficientNetV2B2 for MI detection, CNN—multi-VGG for inner-patient evaluation, SVM for MI detection, and CNN (Table 1). As shown in Figure 5, only a few studies reported poor outcomes, primarily among the earlier publications in the field of AI.

## 4. Discussion

ECG is central to myocardial ischemia assessment, but bedside interpretation has practical limits, with interobserver variability and subtle features that may be overlooked without computer-assisted analysis [26]. In this context, AI has emerged as a potential tool capable of extracting clinically relevant diagnostic information from ECG data. Used as a second reader, it can reduce clinician variation, lower human errors, and enhance clinical decision-making.

This review specifically explores the application of AI in the early detection of MI, which has gained significant interest in recent years, highlighting the growing intersection between digital technologies, especially machine learning, and various aspects of cardiology. A notable observation is China’s dominance in publication output. This reflects national policy support, most prominently the 2017 New Generation Artificial Intelligence Development Plan issued by the State Council, which set milestones for global AI leadership by 2030 and fostered city-level pilot programs, academic–clinical–industry collaborations, and substantial research investment. Consequently, Chinese inventors accounted for about 70% of global generative AI patent family publications between 2014 and 2023, underscoring a strong innovation pipeline and research productivity. In the ECG–AI field specifically, open-access Chinese 12-lead datasets such as the Chapman–Shaoxing collection have lowered entry barriers and provided internationally recognized benchmarks, further driving output and visibility [27]. Many studies report high accuracy, sensitivity, and specificity, but results vary by dataset and evaluation (especially inter-patient/external testing); Figure 5 summarizes this range, with CNNs and SVMs often performing well. CNNs are a subdivision of ANNs that consist of a convolutional, pooling, and fully connected layer that work synergistically to interpret the provided mission effectively. They are specifically designed to capture and process visual patterns in data, making them particularly effective for interpreting signals such as ECG waveforms [28]. CNNs likely predominate because they learn discriminative features directly from raw or transformed waveforms, can exploit multi-lead spatial correlations, and reduce hand-engineered feature dependency, which are advantages that often translate to better cross-dataset robustness when rigorously validated [15]. Recently, Yunfan Chen et al. introduced an advanced version of a CNN, the Multi-Feature Fusion CNN, which moves beyond analyzing morphological or frequency features in isolation. Instead, it integrates both domains to enhance diagnostic performance. This innovative architecture achieved an accuracy of 87%, outperforming the previous version by 6.89% points [29]. This example shows the variability across studies and supports describing performance as a range rather than 99–100%.

Several studies reported strong results with SVMs, particularly when datasets were smaller, inputs used hand-crafted ECG features (e.g., ST-segment shift, QRS shape, frequency measures), and the number of leads was limited. SVM is a supervised method that finds the optimal hyperplane separating classes; a larger margin around the support vectors improves robustness [28]. With kernel functions, SVM can map non-linear ECG patterns into a higher-dimensional space where linear separation is feasible, helping detect subtle ischemic changes that may be missed in the original feature space. These properties fit low-dimensional, engineered inputs and allow class-weights/regularization to address class imbalance [30]. On the other hand, CNNs usually benefit from larger and more diverse training data and from full 12-lead inputs; when data are limited or single-lead, the performance gap becomes small, which explains why some MI detection studies favored SVMs.

SVM pipelines depend on hand-crafted features and careful preprocessing, which can degrade under baseline wander, noise, or device-specific filtering [31,32]. Model behavior is sensitive to kernel choice and hyperparameters (C, γ), and non-linear kernels can overfit small or intra-patient settings, inflating apparent performance. Probabilistic risk estimates are not native and typically require post hoc calibration (e.g., Platt scaling), which many studies omit [33]. Kernel SVMs also scale poorly with large sample sizes or long continuous recordings (time/memory often grow super-linearly with the number of training points), forcing down-sampling or heavy feature summarization [34].

Most of those algorithms used a 12-lead ECG in their AI training and testing. A deep neural network system comprising six layers and trained on over 900,000 standard 12-lead ECG recordings demonstrated high F1 scores across several diagnostic categories, including rhythm disorders (F1 = 0.957), acute coronary syndromes (F1 = 0.925), and conduction abnormalities (F1 = 0.893) [35]. Apart from showing AI’s potential utility in MI detection, these findings also emphasize the enhanced diagnostic performance achievable when leveraging comprehensive 12-lead ECG inputs. Clinically, a 12-lead ECG is the recommended frontline test for suspected ACS and enables anatomical localization (e.g., inferior, anterior, posterior), guiding urgent reperfusion decisions; guideline pathways are anchored in the 12-lead [36]. Single-lead inputs (often lead II) are attractive for ambulatory/wearable screening and can support MI detection in research settings; however, their diagnostic performance for reversible ischemia and regional changes is generally lower than standardized 12-lead acquisition [37]. Continuous ECG/ST-segment monitoring can detect transient or silent ischemia and facilitate earlier triage. However, real-time streams introduce label uncertainty (event timing, noise/artifacts) and distribution shift across care settings, which can reduce external validity unless models are trained and validated accordingly [38].

AI can complement physicians in everyday work. It can serve as a second reader on the first and serial ECGs, flagging borderline ST–T changes and subtle patterns that may be overlooked; in one retrospective evaluation, AI identified MI in ECGs initially interpreted as normal by conventional algorithms [39]. In triage (including prehospital/ED settings), alarm strategies that combine ECG with clinical signs and troponin can route high-risk cases to earlier review and help reduce delays. AI can also assist with signal quality by detecting noise or inconsistent signals before interpretation [40], and computational ECG approaches enable simple comparison with prior recordings to highlight new changes. This way, AI may help reduce missed MI while the final decision remains with the clinician.

In a retrospective evaluation of AI algorithms for detecting MI from ECG initially interpreted as normal but later confirmed to be associated with acute coronary syndromes, the AI excels in detecting 75% of these ECG as MI cases and 86% as abnormal [41]. This reduces human limitations such as missed subtle findings, cognitive fatigue, and interobserver variability. Thus, AI helps prevent diagnosis delays and shortens balloon time.

AI can also support routine workflow by comparing current ECGs with prior ones to flag new changes, prompting repeat ECGs or troponin tests when clinical risk remains, routing high-risk traces earlier in prehospital/ED triage, and detecting poor lead placement or noise before interpretation. Used this way, AI may help reduce missed MI while the final decision remains with the physician.

One of the studies emphasized that certain subtle ECG variations are often misinterpreted as noise, despite their potential to carry significant prognostic value, particularly in post-myocardial infarction risk stratification [42]. To address this challenge, they developed an adaptive downsampling technique that optimizes data processing without compromising diagnostic integrity [42]. Another study introduced the concept of computational ECG, which leverages the integration of portable monitoring devices with cloud-based analytics to enable real-time interpretation of ECG data [43]. These innovations demonstrate the ongoing efforts to enhance the clinical utility of long-duration ECG signals within AI-driven cardiovascular care.

Our review has limitations, including reliance on physician-interpreted ECGs as the reference standard, rather than using definitively diagnosed MI confirmed by clinical outcomes or imaging [44]. This introduces a potential source of bias and affects the model’s validity. Also, two reviewers independently screened titles, abstracts, and full texts, with discrepancies resolved by a third reviewer. Formal inter-rater agreement metrics (e.g., Cohen’s kappa) were not calculated, which may be considered a limitation of the review process. Additionally, the use of small datasets, limited explainability of AI predictions (e.g., through class activation mapping), and the omission of detailed clinical information constrain the generalizability of findings [45]. Beyond dataset size, the heterogeneity of data sources presents another challenge; some studies rely on publicly available databases such as PTB, while others use institution-specific datasets that may not reflect broader population variability. The review highlights considerable variability in reporting practices across studies, including differences in dataset sources, lead configurations, preprocessing steps, algorithm selection, validation strategies, and performance metrics. This variability limits direct comparison of results and interferes with evaluating AI-based interpretations’ overall validity and generalizability. Therefore, there is a clear need for harmonized reporting frameworks, including standardized descriptions of datasets, validation strategies, and performance metrics. Such frameworks would facilitate reproducibility, enable fair benchmarking, and support safe clinical translation of AI-based ECG models for MI detection.

## 5. Conclusions

This scoping review maps the emerging evidence on AI for electrocardiographic detection of myocardial infarction. Building on the heterogeneous datasets, model types, and validation practices we identified, the next step is converging on standardized reporting and evaluation frameworks specific to ECG-AI for MI. At a minimum, future studies should transparently report dataset provenance and case mix; enforce and document patient-level independence; prioritize inter-patient and external validation; provide calibration and decision-threshold rationale; disclose preprocessing and feature pipelines to minimize leakage; share model cards and code/data when feasible; and present stratified performance (e.g., by age, sex, rhythm, comorbidity, and acquisition setting) to surface fairness and generalizability concerns. For clinicians, the current landscape suggests where ECG-AI might assist, e.g., prehospital triage, busy emergency departments, settings without on-site cardiology, yet real-world utility should be established prospectively with clinically meaningful endpoints (time-to-treatment, missed MI, unnecessary activations), human-factors assessment, and integration workflows that preserve clinician oversight. For policymakers and health-system leaders, the path to safe deployment includes setting minimum reporting standards for procurement, requiring external/ongoing performance monitoring and equity audits, enabling privacy-preserving data access for multicenter validation, and supporting interoperability and auditability across vendors. In keeping with the aims of a scoping review, we do not make claims about comparative effectiveness. Rather, we highlight gaps and propose a research agenda: prospective, preregistered, multicenter studies; context-specific implementation trials (prehospital, ED, rural/low-resource hospitals); post-deployment surveillance; and health–economic evaluations. Advancing along this agenda can move ECG-AIs for MI from promising prototypes toward trustworthy, equitable, and clinically actionable tools.

## Figures and Tables

**Table 1:** Family-level diagnostic performance for AMI detection from ECG.

Algorithm Family	Accuracy (Reported)	Sensitivity (Reported)	Specificity (Reported)	Notes/Representative Examples
CNN	Often ≥ 99% (overall model accuracies in the corpus span 70–100%)	Often ≥ 99%	Often ≥ 99%	Multiple CNN studies reported consistently high, though variable, classification (e.g., multi-VGG inner-patient evaluation; other CNN variants). Performance tends to be highest on inner-patient splits and can drop on strict inter-patient validation.
SVM	Often ≥ 99%	Often ≥ 99%	Often ≥ 99%	SVMs trained on engineered ECG features (including ST-T morphology/HRV) frequently matched CNN-level performance; exemplar work reported good results.
ANN (MLP)	Up to ~100% in some reports	Up to ~100% in some reports	Up to ~100% in some reports	ANN using time-domain HRV parameters reported near-perfect performance; results vary with features and validation.
Random Forest	Reported as high in individual studies; no pooled/aggregate family metrics in manuscript text	NA	NA	RF appears among used algorithms (Figure 4), but the narrative does not quantify family-level sensitivity/specificity; see per-study entries in Table S1 for exact values.

NA: Not applicable.

## Data Availability

The original contributions presented in this study are included in the article/Supplementary Material. Further inquiries can be directed to the corresponding authors.

## Supplementary Materials

The following supporting information can be downloaded at: https://www.mdpi.com/article/10.3390/jcm14196792/s1, Table S1: Characteristics and outcomes of included studies.
