# Supplementary material for: Artificial Intelligence for Myocardial Infarction Detection via Electrocardiogram: A Scoping Review
Source: J Clin Med. 2025 Sep 25;14(19):6792. doi: 10.3390/jcm14196792 (PMC12525322; doi:10.3390/jcm14196792)
Supplement: Supplementary file 1 [file jcm-14-06792-s001.zip › jcm-3825479-supplementary.pdf]

Artificial Intelligence for Myocardial Infarction Detection via Electrocardiogram: A Scoping Review

Supplementary material

Table S1. Characteristics and outcomes of included studies.

First Author	Year	Country	AI Model Type	Algorithm	ECG Data Type	Accuracy	AUC
Gibson et al.	2021	Brazil, Colombia, Mexico, Argentina	Deep learning, machine learning, neural networks (specifically 1-D convolutional neural networks)	Convolutional Neural Networks (CNNs)	12-lead EKG, single-lead EKG	90.5% for STEMI detection with Lead V2	NA
Chen et al.	2018	China	Deep learning: Multi-Channel Lightweight Convolutional Neural Network (MCL-CNN)	- Convolutional Neural Networks (CNNs) - Adam Optimizer - Hidden Markov Models (HMMs) - Gaussian Mixture Models (GMMs) - Support Vector Machines (SVM) - Relevance Vector Machine (RVM) - Long Short-Term	Multi-lead EKG (V1, V2, V3), discrete data (500 samples per second)	0.9618	0.955

				Memory (LSTM) networks			
Hao et al.	2019	China	Deep learning, neural networks (specifically ResNet-18, DenseNet-201, shallow neural network)	- Support Vector Machines (SVM) - Classification trees - Hidden Markov Models (HMMs) - Convolutional Neural Networks (CNNs) - ResNet - DenseNet - Shallow neural networks - KNN	12-lead ECG images	0.9473	NA
Wang et al.	2019	China	Deep learning, neural networks (specifically, multi-lead ensemble neural network with sub-networks Net1, Net2, and Net3)	- Traditional algorithms: Support Vector Machines (SVM), threshold, hidden Markov - Deep learning algorithms: Convolutional Neural Networks (CNNs), Multi-feature-branch Convolutional Neural Network (MFCNN), Fully Convolutional Neural Network (FCNN), ResNet, ResNeXt	12-lead EKG, continuous data	- AMI Detection: Sensitivity = 98.35%, Specificity = 97.49%, AUC = 97.92% - IMI Detection: Sensitivity = 93.17%, Specificity = 92.02%, AUC = 92.60%	- AMI: 97.92% - IMI: 92.60%

Zhang et al.	2020	China	Deep learning, Convolutional Neural Network (CNN)	Convolutional Neural Networks (CNNs)	12-lead static EKG	The overall accuracy of the model is close to 95%. Specific accuracy rates include 99.15% for normal rhythm/atrial fibrillation and 98.27% for atrial fibrillation.	NA
Zhao et al.	2020	China	Deep learning, specifically a deep convolutional neural network using Res-Net	Convolutional Neural Networks (CNNs), specifically ResNet	12-lead EKG	- AUC: 0.9954 - Sensitivity: 96.75% - Specificity: 99.20% - Accuracy: 99.01% - Precision: 90.86% - F1 Score: 0.9372	0.9954
Fu et al.	2020	China	Deep learning models: Convolutional Neural Network (CNN), Bidirectional Gated Recurrent Unit (BiGRU), Attention Mechanism	- MLA-CNN-BiGRU (Multi-lead Attention integrated with Convolutional Neural Network and Bidirectional Gated Recurrent Unit) - PCA-MLP (Principal Component	12-lead EKG	- Intra-patient scheme: 99.93% - Inter-patient scheme: 96.50%	NA

				Analysis combined with Multi-Layer Perceptron)			
Sun et al.	2022	China	Machine learning models: decision tree (DT), K-nearest neighbor (KNN), logistic regression (LR), Gaussian kernel SVM, artificial neural network (ANN), random forest (RF), linear SVM	- Support Vector Machines (SVM) - Decision Tree (DT) - K-nearest Neighbor (KNN) - Logistic Regression (LR) - Gaussian kernel SVM - Artificial Neural Network (ANN) - Random Forest (RF)	12-lead EKG, discrete data	- PTB dataset: 94.75% - Independent clinical test dataset: 84.96%	NA
Que et al.	2022	China	Machine learning, probability-driven cellular automata (CA)	NA	12-lead EKG	0.956	NA
Han et al.	2022	China	Deep learning, specifically using a DenseNet network; Knowledge graph	DenseNet network, production rules, knowledge graph	12-lead EKG	- Severity period prediction: 93.65% - MI localization: - Single period and single infarction areas: Average F1 = 86.88% - Single period with multiple infarction	NA

						areas: Overall accuracy = 100.00% - Multiple periods and multiple infarction areas: Overall accuracy = 95.16%	
Zhang et al.	2022	China	Machine learning (bagged decision tree, TreeBagger classifier)	TreeBagger classifier (bagged decision tree)	12-lead EKG	- Total Accuracy: 99.67% - Sensitivity: 99.98% - Specificity: 99.82% - Precision: 99.96% - F1 Score: 0.9997	- The area under the ROC curve is more than 0.88 for every class. - For eight out of eleven cases, the areas under the ROC curve are greater than 0.95. - The area under the PR curve of each class is more than 0.88. - For ASLMI and IPMI with a small number of samples, the final area

							values are greater than 0.99.
Chen et al.	2022	China	Deep learning models: Convolutional Neural Network (CNN), Long Short-Term Memory (LSTM), Convolutional Neural Network-Long Short-Term Memory (CNN-LSTM)	Convolutional Neural Network (CNN), Long Short-Term Memory (LSTM), CNN-LSTM	12-lead EKG, discrete data	- CNN-LSTM: AUC of 0.99 for detecting STEMI - Deep learning models: AUC of 0.96 for discriminating LAD - DL models: AUC of 0.81 for distinguishing RCA from LCX - CNN-LSTM: AUC of 1.00 in Test 1 and 0.99 in Test 2 for STEMI detection - CNN-LSTM: AUC of 0.96 for LAD discrimination - CNN-LSTM: AUC of 0.81 for RCA vs. LCX discrimination	- STEMI detection: 0.99 (CNN-LSTM) - LAD artery discriminati on: 0.96 (Deep learning models) - RCA vs. LCX discriminati on: 0.81 (DL models)
Xu et al.	2022	China	Neural networks, machine learning (federated learning)	Fully connected neural network	NA	- Three-center modeling: 82.3% - Two-center modeling: - Second	NA

						Affiliated Hospital, Zhejiang University School of Medicine and Jiande First People's Hospital: 81% - Second Affiliated Hospital, Zhejiang University School of Medicine and the First People's Hospital of Linping: 79.7% - Single-center modeling: 79%	
Gregg et al.	2022	China	Machine learning, Neural networks	Neural Networks (NN), Repeated Structuring & Learning Procedure (RS&LP)	12-lead ECG	- Differences in QT values: - Validation set: 0.72 ± 22.29 ms (average-based), -0.04 ± 19.42 ms (linear regression-based) - Heart disease dataset: 9.31 ± 21.99 ms (average-	- Learning AUC: 73% to 95% - Testing AUC: 66% to 93%

						based), 7.23 ± 23.79 ms (linear regression-based) - AUC values: - Learning dataset: 73% to 95% - Testing dataset: 66% to 93%	
Wu et al.	2022	China	Machine Learning (LASSO regression)	- Deep learning models (e.g., Res-Net block) - STA-CRNN - LASSO regression	12-lead EKG	- STEMI diagnosis: AUC = 0.94 (internal), AUC = 0.93 (external); Accuracy = 0.85 (internal), Accuracy = 0.84 (external) - LAD/RCA/LCX identification: AUC = 0.92 (internal), AUC = 0.98 (external); Accuracy = 0.84 (internal), Accuracy = 0.95 (external)	- STEMI diagnosis: Internal testing dataset AUC = 0.94, External testing dataset AUC = 0.93 - LAD or non-LAD identificatio n: Internal testing dataset AUC = 0.92, External testing dataset AUC = 0.98
Tang et al.	2023	China	Deep learning, Convolutional Neural	Convolutional Neural Networks	12-lead EKG	0.7	0.75 (95% CI, 0.73 to 0.78)

			Networks (CNNs)	(CNNs) with ResNet-50 backbone architecture and a Squeeze-and-Excitation (SE) module			
Alizadehsani et al.	NA	China	Convolutional neural network and long short-term memory (CNN-LSTM)	Convolutional Neural Network and Long Short-Term Memory (CNN-LSTM)	mini-lead ECG, continuous data	NA	NA
Wang et al.	NA	China	Deep learning models: Convolutional Neural Network (CNN) and Long Short-Term Memory (LSTM)	Convolutional Neural Network (CNN), Long Short-Term Memory (LSTM)	12-lead EKG	- Accuracy: 0.997 - Precision: 0.802 - Recall: 0.977 - Area under the receiver operating characteristic curve: 0.999 - F1 score: 0.881 - Specificity: 0.998	0.999
Abebe et al.	NA	China	Deep learning models, including convolutional neural networks (CNNs), recurrent neural networks (RNNs), and CNN-LSTM architectures,	- Convolutional Neural Networks (CNNs) - Recurrent Neural Networks (RNNs) - Gradient Boosted Tree	12-lead EKG	- Normal: 96.7% - Acute: 82.9% - Recent: 68.6% - Old: 73.8%	- Acute: 96.7% - Recent: 82.9% - Old: 68.6% - Normal: 73.8%

			with transfer learning from existing networks like GoogLeNet and MnasNet.				
Yao et al.	NA	China	Machine learning (ML), Deep learning (ResNet-18), Ensemble ML algorithm (Extreme Gradient Boosting)	- Gaussian Naive Bayes (GNB) - Support Vector Machine (SVM) - Extreme Gradient Boosting (XGBoost) - Residual Neural Network with 18 layers (ResNet-18)	single-lead EKG	0.9616	NA
Liu et al.	2018	China	Deep learning, specifically a 13-layer deep convolutional neural network (CNN)	Convolutional Neural Networks (CNNs)	Single-lead EKG (lead II), continuous data discretized into 3-second segments	- Denoised ECG signal: 99.34% - Original ECG signal: 98.59%	NA
Zhang et al.	2019	China	- Deep learning: Principal Component Analysis Network (PCANet) - Machine learning: Support Vector Machines (SVM), Linear Discriminant	- Support Vector Machines (SVM) - Linear Discriminant Analysis (LDA) - Back propagation neural network (BP) - K-nearest neighbor classifier (KNN)	Single-lead EKG (Lead II)	- Class-oriented experiments: 99.49% - Patient-specific experiments: 93.17%	- Model with noise: 0.9991 - Denoising model: 0.9955

			Analysis (LDA), back propagation neural network (BP), k-nearest neighbor (KNN), Random Forests (RF)	- Random Forests (RF)			
Han et al.	2019	China	Machine learning models: Support Vector Machine (SVM), Principal Component Analysis (PCA), Linear Discriminant Analysis (LDA), Locality Preserving Projection (LPP), Back Propagation Neural Network (BPNN), Bagging tree	- Support Vector Machines (SVM) - Back Propagation Neural Networks (BPNN) - Bagging Trees	12-lead EKG	- Intra-patient paradigm: 99.81% - Inter-patient paradigm: 92.69%	NA
Feng et al.	2019	China	Convolutional Neural Network (CNN), Long-Short Term Memory Network (LSTM)	- Convolutional Neural Networks (CNNs) - Long-Short Term Memory Network (LSTM) - Support Vector Machines (SVM)	Single-lead EKG	0.954	0.9868

				- K-Nearest Neighbor (KNN) - Pattern Recognition Methods			
Khosla et al.	2020	China	- Random Forests - Support Vector Machine (SVM) - Back Propagation Neural Network (BPNN) - K-Nearest Neighbor (KNN)	- Random Forests (RF) - Support Vector Machines (SVM) - Back Propagation Neural Network (BPNN) - K-Nearest Neighbor (KNN)	12-lead EKG	- Intra-patient scheme: 99.71% - Inter-patient scheme: 85.82%	NA
Zeng et al.	2020	China	Neural networks (specifically RBF neural networks)	- Wavelet transforms (WT, CWT, PSWT, DWT) - Kalman filtering - Least mean squares algorithm - Ensemble learning - Artificial neural networks (ANN) - Adaptive neuro-fuzzy inference system (ANFIS) - Support vector machine (SVM) - Deep learning - Convolutional neural networks (CNN) - Random	12-lead ECG and Frank XYZ leads (15 leads in total)	0.9798	NA

				forest - J48 decision tree - Back propagation neural network - Least-squares support vector machine - Dynamical estimators with constant RBF neural networks			
Han et al.	2020	China	Deep learning (specifically, multi-lead residual neural network (ML-ResNet))	- Traditional machine learning algorithms: K Nearest Neighbors (KNN), Support Vector Machines (SVM), Decision Trees (DT), Naive Bayes (NB), Back Propagation Neural Networks (BPNN) - Deep learning methods: Convolutional Neural Networks (CNNs), Recurrent	12-lead EKG, discrete data	- MI Detection (Inter-patient): 95.49% - MI Detection (Intra-patient): 99.92% - MI Location (Intra-patient): 99.72%	- Intra-patient scheme: 0.9999 - Inter-patient scheme: 0.98

				Neural Networks (RNNs), Restricted Boltzmann Machines (RBMs), Autoencoders (AEs) - Proposed model: Multi-Lead Residual Network (ML-ResNet)			
Zhang et al.	2021	China	Machine learning (bagged decision tree, TreeBagger classifier)	TreeBagger classifier (a type of Random Forest) and Parallel Factor Analysis	12-lead EKG	- Detection Accuracy: 99.88% - Localization Accuracy: 99.40%	NA
Xiong et al.	2021	China	Deep learning (DenseNet)	DenseNet (a type of Convolutional Neural Network)	12-lead EKG	- Accuracy: 99.87% - Sensitivity: 99.84% - Specificity: 99.98%	NA
He et al.	2021	China	Deep learning, neural networks	- Convolutional Neural Networks (CNNs) - Support Vector Machines (SVM) - k-Nearest Neighbor (KNN)	12-lead EKG	- Intra-patient scheme: 99.63% - Patient-specific scheme: 96.99% - Hybrid database (PTB and PTB-XL): 94.19%	NA

Cao et al.	2021	China	Deep learning (Convolutional Neural Networks), Machine learning	- Convolutional Neural Networks (CNNs) - Support Vector Machines (SVM) - k-nearest neighbor (KNN) - Linear discriminant (LD) - Decision tree (DT) - Wavelet-transform	12-lead ECG data with specific use of leads V2, V3, V5, and aVL	- Accuracy (ACC): 96.65% - Specificity (Spec): 97.72% - Sensitivity (Sen): 94.30%	0.9671
Ge et al.	2016	China	Machine learning (SVM, binary decision tree)	- Support Vector Machine (SVM) - Binary Decision Tree	- Type of EKG data used: Both 12-lead SECG and 3-lead OECG - Data type: Continuous data - Sampling rate: 1000 samples per second	- 100 Hz FMAR: - HC: 95.63% - MIES: 94.21% - MIAS: 98.83% - AVG: 96.22% - 250 Hz FMAR: - HC: 99.54% - MIES: 98.49% - MIAS: 99.91% - AVG: 99.31% - 500 Hz FMAR: - HC: 98.48% - MIES: 99.50% - MIAS: 99.49% - AVG: 99.15%	NA
Liu et al.	2018	China	Deep learning, Convolutional Neural Network (CNN)	Convolutional Neural Networks (CNNs)	12-lead EKG, discrete data	- Class-based MI detection: 99.95% - Class-based MI localization: 99.81% - Patient-specific	0.9961

						MI detection: 98.79% - Patient-specific MI localization: 94.82%	
Fournier et al.	2021	China and USA (based on dataset origins and ethics committee location)	Deep learning models, specifically residual networks and convolutional neural networks	Convolutional Neural Networks (CNNs) with a residual network architecture	12-lead EKG, discrete data (500 Hz and 100 Hz sampling), continuous over 10 seconds	- AUC: Training set = 0.964, Validation set = 0.944, Testing set = 0.977 - Precision: Training set = 0.827, Validation set = 0.789, Testing set = 0.830 - Sensitivity: Training set = 0.824, Validation set = 0.818, Testing set = 0.951 - Specificity: Training set = 0.950, Validation set = 0.913, Testing set = 0.951 - F1 score: Training set = 0.825, Validation set = 0.803, Testing set = 0.886	- Training set: 0.964 (95% CI: 0.961-0.966) - Validation set: 0.944 (95% CI: 0.939-0.949) - Testing set: 0.977 (95% CI: 0.961-0.991)
Zeng et al.	2024	China, USA	Neural networks (specifically	- k-nearest neighbor (KNN) - Support Vector	Single-lead (lead II) ECG signals	0.9921	NA

			radial basis function neural networks and dynamical RBFNNs), deterministic learning	Machine (SVM) - Self-organizing maps with learning vector quantization - Decision trees - Naïve Bayes (NB) - Random Forest (RF) - Artificial Neural Networks (ANNs) - Linear discriminants - Convolutional Neural Networks (CNN) - Long Short-Term Memory Networks (LSTM) - Hybrid CNN-LSTM - Generative Adversarial Networks - Deterministic learning based dynamical estimators			
Martin et al.	NA	Europe	Deep learning, specifically Long-Short Term Memory (LSTM) neural networks	- Convolutional Neural Networks (CNNs) - Support Vector Machines (SVMs) -	Single-lead (Lead II) electrocardiogra m	- PTB database: 77.12% - PTB-XL validation set: 85.07% - PTB-XL test set: 84.17%	NA

				Random Forests - Long Short-Term Memory (LSTM) networks			
Hannig et al.	2022	Germany	- Convolutional Neural Network (CNN) - Long Short-Term Memory (LSTM) - Convolutional Recurrent Neural Network (CRNN) - Gated Recurrent Unit (GRU) - Residual Neural Network (ResNet) - Autoencoder (AE)	- Deep Learning Models: Convolutional Neural Network (CNN), Long Short-Term Memory (LSTM), Convolutional Recurrent Neural Network (CRNN), Gated Recurrent Unit (GRU), Residual Neural Network (ResNet), Autoencoder (AE) - Traditional Machine Learning Models: Support Vector Machines (SVM), Random Forest (RF), Naive Bayes, Decision Tree (DT), K-Nearest Neighbor	12-lead EKG, single-lead EKG	The reported maximum accuracies of the six different methods are all beyond 97%. The highest performance has been obtained from CNN and ResNet models, with higher accuracy achieved using 12-lead ECG data from the PTB database.	NA

				(KNN), Neural Network (NN)			
Polz-Dacewicz et al.	2022	China	Deep learning (BiLSTM, LSTM), Neural networks (BiLSTM, LSTM)	- Fourier transform - Discrete wavelet transform - Artificial Neural Networks (ANN) - Support Vector Machines (SVM) - K-Nearest Neighbors (KNN) - Convolutional Neural Networks (CNN) - DenseNet - Bidirectional Long Short-Term Memory (BiLSTM)	12-lead EKG	- Accuracy of morphological feature + BiLSTM algorithm in MI detection: 99.4% - Overall accuracy: 98.6% - Sensitivity, specificity, PPV, NPV, and F1 score: all above 98.4% - Kappa coefficient: 0.983	NA
He et al.	2022	China	Deep learning, neural networks	Convolutional Neural Networks (CNNs), Softmax classifier	2-D vectorcardiogram (VCG)	0.9987	NA
Zeng et al.	2022	Unkown	- Neural networks - Dynamical RBF neural networks - SVM classifier with RBF Kernel - CNN classifier	- Support Vector Machines (SVM) - Convolutional Neural Networks (CNN)	15-lead ECG data (12-lead ECG signals synthesized with Frank XYZ leads)	0.982	NA

Xiong et al.	2023	China	Deep learning models: DenseNet, GRU (Gated Recurrent Unit)	- Convolutional Neural Networks (CNNs) - Densely Connected Convolutional Networks - Multi-scale Convolutional Neural Networks - Recurrent Neural Networks (RNNs) like BiLSTM - Discrete Wavelet Transform (DWT) - Support Vector Machines (SVM) - Rough Set Classifiers - K-Nearest Neighbor (KNN) Classifiers	3-lead EKG (leads II, III, and AVF), continuous data	- Intra-patient scheme: 99.95% - Inter-patient scheme: 88.68%	NA
Liu et al.	2023	multicountry	Deep learning, neural networks (specifically, a transformer model)	- Convolutional Neural Networks (CNNs) - Recurrent Neural Networks (RNNs) - Long	12-lead EKG	99.25% (detection), 68.45% (localization)	NA

				Short-Term Memory Neural Networks (LSTMs) - Hybrid CNN-RNN models - Transformer models - Multi-lead Residual Neural Networks (ML-ResNet) - Multi-feature Branching Convolutional Bidirectional Recurrent Neural Networks (MFB-CBRNN) - Multichannel Lightweight Models (ML-Net)			
Parmar et al.	2024	Unkown	Machine learning (ML), Spiking Neural Networks (SNNs), Neural Networks	Spiking Neural Network (SNN)	8-lead discrete ECG data	0.999	NA
Wang et al.	2024	China	Deep learning models: CNN, LSTM, GRU, ResNet, DenseNet; Neural	- Convolutional Neural Networks (CNNs) - Long Short-Term Memory (LSTM)	3-lead EKG (leads II, III, and V2), discrete data (2-second episodes)	0.991	NA

			networks: ISRM-LSTM, ISRM-GRU	- Gated Recurrent Unit (GRU) - Residual Networks			
Qiang et al.	2024	China	Deep learning (DL), specifically a multi-channel dense attention neural network (MCDANN)	- Convolutional Neural Networks (CNNs) - Residual Networks (ResNet) - Multibranch densely connected convolutional network (MB-DenseNet) - Multilead branch with the residual network integrated with squeeze and excitation networks and bidirectional long short-term memory (LSTM) model (MLB-ResNet-SENet-BL)	12-lead EKG	- Intra-patient MI detection: 99.94% - Inter-patient MI detection: 98.27% - Intra-patient MI localisation: 99.85% - Inter-patient MI localisation: 81.70%	NA
Zhang et al.	NA	multiple country	Deep learning, Neural networks	- Random Forest - KNN - ANN - CNN - SVM - RFE - PCA	8-lead EKG	0.9984	NA

			(specifically BiGRU)	- HMM - GMM - CART - SAE			
Liu et al.	2021	China	Deep learning (Convolutional Neural Networks - CNNs)	Convolutional Neural Networks (CNNs)	12-lead EKG	- Detection Accuracy: 99.51% - Localization Macro-F1: 99.14%	NA
He et al.	2022	Multicountry	Deep learning, machine learning, neural networks (specifically, multibranch densely connected convolutional network (MB-DenseNet))	- Convolutional Neural Networks (CNNs) - DenseNet - ResNet - Active Learning (AL) - Semi-Supervised Learning (SSL)	12-lead EKG	- Intra-patient scheme: 99.87% - Patient-specific scheme: 96.09%	NA
Acharya et al.	2017	Multicountry	Deep learning, specifically an eleven-layer deep convolutional neural network (CNN)	Convolutional Neural Networks (CNNs)	Single-lead EKG (lead II), discrete data	- With noise: 93.53% - Without noise: 95.22%	NA
Zhang et al.	2022	Unkwon	Machine learning (TreeBagger classifier)	TreeBagger classifier	3-lead Vectorcardiogram (VCG)	0.998	The area under the receiver operating characteristic curves (AUC) is more than 0.88 for

							each type of VCG signal.
Deng et al.	2020	China	- Convolutional Neural Networks (CNNs) - Recurrent Neural Networks (RNNs) - Autoencoders (AEs) - Deep Belief Neural Networks (DBNs) - Spark-trace Network (ST-Net)	- Convolutional Neural Networks (CNNs) - Recurrent Neural Networks (RNNs) - Autoencoders (AEs) - Deep Belief Neural Networks (DBNs) - Support Vector Machines (SVMs) - K-Nearest Neighbors (KNNs) - Gaussian Mixture Models (GMMs) - Linear Discriminant Analysis (LDA) - Hidden Markov Methods (HMMs) - Multiple Instance Learning (MIL)	Single-lead ECG (Lead-I)	- Accuracy: 98.13% - Sensitivity: 98.19% - Specificity: 98.09%	NA
Jahmunah et al.	2022	Multicountr y	Deep learning models: DenseNet and CNN	DenseNet, CNN (Convolutional Neural Networks)	12-lead EKG, discrete data	- DenseNet model: Average accuracy rate of 98.9% - CNN	NA

			(Convolutional Neural Network)			model: Average accuracy rate of 98.5%	
Kapfo et al.	2020	India	Machine learning: K-nearest neighbour (KNN), Support Vector Machine (SVM) with linear and radial basis function (RBF) kernels	- K-nearest neighbour (KNN) - Support Vector Machine (SVM) - SVM with radial basis function (RBF) kernel	12-lead EKG	0.9988	NA
Yadav et al.	2021	India	Convolutional Neural Network (CNN), Deep Learning	Convolutional Neural Network (CNN)	12-lead EKG, continuous data; also includes vectorcardiogram (VCG) with three leads	0.9982	1
Kora et al.	2017	India	- Artificial Neural Networks (ANN) - Levenberg-Marquardt Neural Network (LMNN) - Support Vector Machine (SVM) - K-Nearest Neighbor (KNN) - Hybrid Firefly and Particle Swarm	- K-Nearest Neighbor (KNN) - Support Vector Machine (SVM) - Levenberg Marquardt Neural Network (LMNN)	Discrete data from MIT-BIH PTB database; lead type not explicitly mentioned	0.993	NA

			Optimization (FFPSO)				
Sharma et al.	2017	India	Machine learning: Support Vector Machine (SVM), K-Nearest Neighbor (KNN)	- Support Vector Machines (SVM) - K-Nearest Neighbors (KNN)	12-lead ECG data, specifically using leads II, III, and aVF; discrete data sampled at 250 Hz	- Class-oriented approach: - KNN: Accuracy = 98.69% - SVM: Accuracy = 98.84% - Subject-oriented approach: Average Accuracy = 81.71%	- KNN: 0.9945 - SVM: 0.9994
Prabhakarar ao et al.	2020	India	Deep learning: Recurrent Neural Networks (RNN), Attention Mechanism	- Machine Learning (ML) approaches: - Threshold-based methods - K-nearest neighbor (KNN) - Support Vector Machines (SVM) - Random Forest - Neural Networks (NN) - Deep Learning (DL) approaches: - Convolutional Neural Networks (CNNs) - Recurrent Neural	12-lead EKG, continuous data (4-second segments)	0.9779	0.98

				Networks (RNNs) with attention mechanisms			
Kumar et al.	2022	India	- Support Vector Machine (SVM) - Artificial Neural Network (ANN)	- Support Vector Machines (SVM) - Artificial Neural Networks (ANN)	Single-lead EKG (lead-II), continuous data	- ANN with time-domain HRV parameters: 100% for CAD, 99.6% for MI - SVM: 75.5% for CAD, 98.9% for MI - Three-class model with SVM and time-domain features: 98.7%	NA
Jahnavi et al.	2023	India	Machine Learning (Random Forest classifier)	Random Forest (RF)	Derived limb leads from leads I and II (not a traditional 12-lead setup)	0.98	NA
Sahu et al.	2024	India	Deep learning models: Convolutional Neural Network (CNN), Long Short-Term Memory (LSTM), Gated Recurrent Unit (GRU)	- Convolutional Neural Networks (CNNs) - Support Vector Machines (SVM) - Ensemble classifiers - K-nearest neighbor (KNN) - Multilayer perceptron (MLP) - Long	12-lead EKG, single-lead analysis	- Intra-patient MI detection: 99.96% (CNN-LSTM on lead-V2) - Inter-patient MI detection: 90.15% (CNN-GRU on lead-V5) - Intra-patient MI localization: 99.87% (CNN-	- CNN-LSTM: AUROC > 0.99 - CNN-GRU: AUROC > 0.9

				Short-Term Memory (LSTM) - Gated Recurrent Units (GRUs)		LSTM on lead-V1) - Inter-patient MI localization: 66.32% (CNN-GRU on lead-V1)	
Pharvesh et al.	2020	India	- Machine Learning (ML) models: - K-nearest neighbour (KNN) - Support Vector Machine (SVM) - Decision Trees (DT) - Logistic Regression (LG) - Deep Learning (DL) model: - 1-dimensional Convolutional Neural Network (CNN)	- K-nearest neighbor (KNN) - Support Vector Machine (SVM) - Convolutional Neural Network (CNN) - Logistic Regression (LG) - Decision Trees (DT)	12-lead EKG, discrete data	- KNN: F1-score of 0.97 for MI detection and 0.94 for localization - SVM (RBF): F1-score of 0.96 for detection and 0.92 for localization - CNN: F1-score of 0.99	- CNN: 99.9% - KNN: 94.4% - SVM: 92.0% - DT: 87.9% - LG: 71.7%
Sridhar et al.	2020	India, Singapore, USA, Lebanon, Taiwan, Japan	- Machine learning models: k-Nearest Neighbor (KNN), Support Vector Machine (SVM), Probabilistic Neural Network (PNN), Decision Tree (DT) -	- k-Nearest Neighbor (KNN) - Support Vector Machine (SVM) - Probabilistic Neural Network (PNN) - Decision Tree (DT)	Single-lead ECG (Lead II), discrete data	0.9796	NA

			Deep learning models: planned for future work (autoencoder, Recurrent Neural Network (RNN), deep generative models)				
Darmawahyuni et al.	2019	Indonesia	- Deep Learning - Recurrent Neural Network (RNN) - Long Short-Term Memory (LSTM) - Gated Recurrent Unit (GRU)	- Recurrent Neural Network (RNN) - Long Short-Term Memory (LSTM) - Gated Recurrent Unit (GRU)	15-lead EKG	- Sensitivity: 98.49% - Specificity: 97.97% - Precision: 95.67% - F1-score: 96.32% - BACC: 97.56% - MCC: 95.32%	NA
Darmawahyuni et al.	2019	Indonesia	Deep learning models: Long Short-Term Memory (LSTM), Recurrent Neural Network (RNN)	Convolutional Neural Networks (CNNs), Long Short-Term Memory (LSTM)	Continuous data from the Physionet PTB Diagnostic ECG Database (PTBDB), digitized at 1000 samples per second, segmented into 4-second windows.	0.83	NA
Yahyaie et al.	2019	Iran	Machine learning: Artificial Neural Network (ANN), Multilayer Perceptron	Multilayer Perceptron Neural Network (MLPNN) with Back	Three-lead EKG, continuous data	0.895	NA

			Neural Network (MLPNN) with Back Propagation (BP) algorithm	Propagation (BP) algorithm			
Hafshejani et al.	2021	Iran	- Classification and Regression Tree (CART) - Feedforward Neural Network (FFNN)	- Classification and Regression Tree (CART) - Feedforward Neural Network (FFNN)	12-lead EKG and 3-lead VCG	- Discrimination between MI and HC cases: 99.4% - Localization of MI to anterior and inferior regions: 98.9%	0.99
Hussein et al.	2021	Iraq	Support Vector Machine (SVM) with Radial Basis Function (RBF)	Support Vector Machines (SVM)	Multi-lead EKG, continuous data over 1 min intervals	0.9909	NA
Bodini et al.	2020	Italy	Machine Learning (Random Forest)	Random Forest (RF)	NA	The outcome accuracy ranges from 0.84 to 0.92.	NA
Goto et al.	2019	Japan	Deep learning, specifically neural networks with one-dimensional convolution and bidirectional LSTM layers	Convolutional Neural Networks (CNNs), Long Short-Term Memory (LSTM) layers	12-lead EKG, discrete data	0.83 (95% CI 0.79-0.88)	- Derivation cohort: 0.89 (95% CI 0.84-0.92) - Validation cohort: 0.88 (95% CI 0.84-0.93)
Shimizu et al.	2022	Japan	Machine learning (ML) models used include light gradient	- Light Gradient Boosting Machine (LGBM) - Extra Tree Classifier	12-lead EKG, discrete data	- Highest accuracy for TTS using a specific ECG parameter: V1	- For aVR STmid: 0.727 - For model_LGBM: 0.868 -

			boosting machine (model_LGBM) and extra tree classifier (model_ET). Conventional deep learning procedures were not used.	(ET) - Ensemble Learning Models - Decision Trees		STJ at 118 mV (0.773) - Highest accuracy for ML models: model_LGBM (0.842), model_ET (0.831)	For model_ET: 0.896
Alimbayeva et al.	2019	Kazakhstan	Neural networks, specifically LVQ (Learning Vector Quantization) and Kohonen Self-Organizing Maps (KSOM)	- Feedforward neural networks (e.g., multilayer perceptron) - Recurrent neural networks (e.g., Kohonen network) - LVQ (Learning Vector Quantization) networks - Kohonen Self-Organizing Maps (KSOM)	12-lead EKG	Specificity: 89%, Sensitivity: 76%	NA
Park et al.	2019	Korea	Deep learning (Convolutional Neural Network - CNN), Machine learning (kNN, SVM, DT)	Convolutional Neural Networks (CNNs), k-Nearest Neighbors (kNN), Support Vector Machines (SVM), Decision Trees (DT)	12-lead EKG	- Initial: Sensitivity = 0.685, Specificity = 0.350, AUC = 0.526 - After preprocessing: Sensitivity = 0.932, Specificity =	0.943

						0.896, AUC = 0.943	
Heo et al.	2020	Korea	Machine learning (KNN classifier)	K-nearest neighbor (KNN) classifier; Convolutional Neural Networks (CNNs) mentioned as potential for future use	12-lead EKG	0.9637	NA
Sraitih et al.	2022	Morocco	Machine learning: Support Vector Machine (SVM), K-Nearest Neighbors (KNN), Random Forest (RF)	- Support Vector Machine (SVM) - K-Nearest Neighbors (KNN) - Random Forest (RF)	12-lead EKG, discrete data	- SVM: 0.68 - KNN: 0.65 - RF: 0.74	NA
Abbas et al.	2024	Multicountries	Deep Learning (DL) models: Recurrent Neural Network (RNN), Convolutional Neural Network (CNN), Deep Neural Network (DNN), Long Short-Term Memory (LSTM)	- Recurrent Neural Network (RNN) - Convolutional Neural Network (CNN) - Deep Neural Network (DNN) - Long Short-Term Memory (LSTM)	12-lead EKG, continuous time-series data	- Binary classification: 99.39% - Multiclass classification: 99.74%	NA
Sbrollini et al.	2023	Netherlands	Deep learning, neural networks, machine	Neural Networks (NNs) created by Advanced	12-lead EKG	- Median AUC for NNs in testing dataset: 83% - Median	0.83

			learning (specifically logistic regression)	Repeated Structuring and Learning Procedure (AdvRS&LP)		Sensitivity for NNs in testing dataset: 77% - Median Specificity for NNs in testing dataset: 89%	
Leur et al.	NA	Netherlands	Deep learning, specifically a 37-layer convolutional residual deep neural network	Convolutional Neural Networks (CNNs)	12-lead EKG	- Overall concordance statistic: 0.93 (95% CI, 0.92-0.95) - Polytomous discriminatory index: 0.83 (95% CI, 0.79-0.87) - Undertriage rate: 8.9% - Overtriage rate: 11% - Sensitivity, specificity, PPV, and NPV are provided in Table 2.	- Overall: 0.93 - Normal: 0.95 - Abnormal, Not Acute: 0.91 - Abnormal, Subacute: 0.94 - Abnormal, Acute: 0.94
Domingo-Gardeta et al.	2024	Netherlands, Spain, Latin America (countries not specified)	Deep learning	Deep learning based algorithm (likely involving neural networks such as CNNs)	12-lead EKG	- Accuracy of OMI diagnosis through ECG analysis compared with OMI clinical label from angiography reports. - Metrics for	NA

						performance evaluation: accuracy, positive predictive value, negative predictive value, sensitivity, specificity, F1-score, area under the curve ROC. - Evaluation across different subgroups: age categories, sex, presence/absence of risk factors, lab measurements, ECG cardiac patterns.	
Alghamdi et al.	2019	NA	Deep learning (Convolutional Neural Network - CNN), Machine learning (Q-Gaussian multi-class support vector machine - QG-MSVM)	Convolutional Neural Networks (CNNs), Support Vector Machines (SVMs)	Single-lead ECG data	- VGG-MI1: 99.02% - VGG-MI2: 99.22%	NA
Mehta et al.	2020	NA	Deep learning (Convolutional Neural	Convolutional Neural	Single-Lead and 12-lead ECG	NA	NA

			Networks - CNN)	Networks (CNNs)			
Fatimah et al.	2021	NA	Machine learning models: k-nearest neighbor (kNN), support vector machine (SVM), ensemble bagged trees (EBT), ensemble subspace k-nearest neighbors (ESkNN)	- k-nearest neighbor (kNN) - Support Vector Machine (SVM) - Ensemble Bagged Trees (EBT) - Ensemble subspace k-nearest neighbors (ESkNN)	Single-lead ECG data (lead II), discrete data sampled at 1000 Hz with 16-bit resolution, continuous over 24 hours	- Primary algorithm with kNN classifier: 99.96% - Modified algorithm: 99.65%	NA
Liu et al.	2021	NA	Deep Learning (DL), Neural Networks (specifically Multiple-Branch Networks), Machine Learning (through Genetic Algorithm optimization)	- Traditional machine learning algorithms: Support Vector Machines (SVM), K-Nearest Neighbors (KNN), Decision Trees (DT), Random Forests (RF) - Deep learning models: Convolutional Neural Networks (CNNs), Recurrent	12-lead EKG, discrete data	- MI Detection on PTB Database: 97.11% - MI Detection on PTB-XL Database: 90.80% - MI Localization on PTB Database: 71.65% - MI Localization on PTB-XL Database: 75.18%	NA

				Neural Networks (RNN), Multiple-Branch Networks (MBNs)			
Li et al.	2022	NA	Deep learning models: Generative Adversarial Networks (GANs), Convolutional Neural Networks (CNNs)	- Support Vector Machines (SVM) - k-nearest neighbors (KNN) - Naïve Bayes - Decision Trees - Convolutional Neural Networks (CNNs) - Recurrent Neural Networks (RNNs) - Generative Adversarial Networks (GANs)	Single-lead ECG data	0.9906	NA
He et al.	2023	NA	Deep learning model (Multi-branch Residual Shrinkage Network)	Convolutional Neural Networks (CNNs), Residual Networks (ResNets)	12-lead EKG	- Intra-patient scheme: 99.89% - Patient-specific scheme: 98.35%	NA
Harnod et al.	2024	NA	- Sparse Representation Classification (SRC) - Artificial	Sparse Representation Classification (SRC), Artificial	12-lead EKG	0.86	NA

			Neural Network (ANN) - Euclidean Distance (ED) comparison	Neural Networks (ANN), Euclidean Distance (ED) comparison			
Kolliyil et al.	2024	NA	Machine learning (eXtreme Gradient Boosting - XGBoost)	eXtreme Gradient Boosting (XGBoost)	12-lead EKG	- AUC: 0.86 - Weighted F1 scores: - Conduction disturbance: 86% - MI: 84% - Hypertrophy: 90% - ST/T change: 86%	0.86
Abdul et al.	2024	NA	- Deep learning: Convolutional Neural Network (CNN) - Machine learning: k-Nearest Neighbor (kNN)	- Convolutional Neural Network (CNN) - k-Nearest Neighbor (kNN) - Support Vector Machines (SVM) - Discrete Wavelet Transform (DWT) - Artificial Neural Networks - Long Short-Term Memory (LSTM)	Single-lead continuous data	- Arrhythmia: 98% - Myocardial Infarction: 97.4%	NA
Wu et al.	NA	NA	Deep learning, Sparse Autoencoder (SAE), Multiple Layer	- Deep learning (specifically sparse autoencoder (SAE)) -	12-lead EKG, discrete data	- Sensitivity: 99.90% (non-MI), 99.72% (MI-1), 99.66% (MI-2) -	NA

			Perceptron (MLP), Softmax Regression	Softmax regression - ANN (Artificial Neural Networks) - Neural-fuzzy - KNN (K-Nearest Neighbors) - MIL+SVM (Multiple Instance Learning with Support Vector Machines)		Specificity: 99.75% (non-MI), 99.95% (MI-1), 99.95% (MI-2)	
Yousuf et al.	NA	NA	- Machine Learning (ML) - Deep Learning (DL) - Neural Networks (specifically, a custom multilayer 2-D-CNN)	Convolutional Neural Networks (CNNs)	Single-lead EKG (lead-II) from a public dataset (PTB ECG dataset from PhysioNet), discrete data	- GASF dataset with noise and baseline wander: 99.68% - GADF dataset with noise and baseline wander: 99.80% - GASF dataset with noise and baseline wander removed: 99.82% - GADF dataset with noise and baseline wander removed: 99.84%	NA

Parupudi et al.	NA	NA	Deep learning (DL), Transfer learning (TL), Machine learning (ML)	Convolutional Neural Networks (CNNs), specifically VGG16, VGG19, ResNet50, EfficientNetV2B2, and DenseNet201	12-lead EKG, discrete data	0.9934	NA
Tripathy et al.	NA	NA	Machine learning: k-nearest neighbor (KNN), fuzzy KNN	K-nearest neighbor (KNN), fuzzy KNN	Multilead ECG data	- All Features: Fuzzy KNN - 84.96% - Selected Features using ANOVA: Fuzzy KNN - 86.09% - Selected Features using SD: Fuzzy KNN - 85.09% - All Features: KNN - 84.29% - Selected Features using ANOVA: KNN - 84.28% - Selected Features using SD: KNN - 84.61%	NA
Oshida et al.	NA	NA	Machine learning	NA	NA	The outcome accuracy is exceeding 90%.	NA

Bhatt et al.	2024	NA	Echo state network (a type of recurrent neural network)	NA	NA	3.5% of transmissions were classified as 'Red' or 'Yellow' alerts after technician review, indicating the outcome accuracy of the remote monitoring system.	NA
Bond et al.	2022	NA	Machine learning	NA	12-lead EKG	The outcome accuracy of using ECGs for diagnosing acute myocardial ischemia is compromised due to limitations such as lead misplacement, overconfidence in machine interpretation, and the challenge of linking subtle changes in cardiac currents to	NA

						perfusion deficits.	
Cho et al.	2020	NA	Deep learning-based artificial intelligence algorithm (DLA), Convolutional neural network (CNN), Variational autoencoder (VAE)	Convolutional Neural Networks (CNNs), Variational Autoencoder (VAE)	12-lead EKG and 6-lead EKG; discrete data	- AUROC for 12-lead ECG: Internal validation = 0.902, External validation = 0.901 - AUROC for 6-lead ECG with VAE: Internal validation = 0.880, External validation = 0.854 - AUROC for 6-lead ECG without VAE: Internal validation = 0.747, External validation = 0.726 - Sensitivity, Specificity, PPV, NPV for 12-lead ECG: Internal validation = Sensitivity: 83.0%, Specificity: 89.4%, PPV: 58.7%, NPV: 96.7%; External	- 12-lead ECG: 0.902 (internal), 0.901 (external) - 6-lead ECG with VAE: 0.880 (internal), 0.854 (external) - 6-lead ECG without VAE: 0.747 (internal), 0.726 (external) - STEMI detection: - 12-lead ECG: 0.992 (internal), 0.951 (external) - 6-lead ECG with VAE: 0.974 (internal), 0.925 (external)

						validation = Sensitivity: 84.4%, Specificity: 88.5%, PPV: 51.8%, NPV: 97.5% - AUROC for STEMI detection: Internal validation = 0.992 (12-lead), 0.974 (6-lead with VAE); External validation = 0.951 (12-lead), 0.925 (6-lead with VAE)	
authors) et al.	NA	NA	Deep learning, Convolutional Neural Networks (CNN)	Convolutional Neural Networks (CNNs)	12-lead EKG	- Overall multiclass accuracy: 97.86% - Binary detection accuracies: - Normal ECG detection: 98% - Abnormal heartbeat detection: 97% - History of MI detection: 96% - MI detection: 99% - Sensitivity and	NA

						specificity: - MI class: sensitivity = 0.99, specificity = 0.99 - Normal class: sensitivity = 0.98, specificity = 1.0	
Qiang et al.	2024	NA	Deep learning models, specifically convolutional neural networks (CNNs) with multi-view learning, multi-task learning, and knowledge distillation techniques.	- Traditional machine learning algorithms: SVM, Random Forest, Decision Tree, K-Nearest Neighbor (KNN) - Deep learning models: CNNs, RNNs, ResNets, Transformers - Baseline deep networks: LSTM, Bi-LSTM, FCN, ResNet1d, Xresnet1d101, ViT, Inception1d - State-of-the-art networks: SE-ResNet, Transformer, ATI-CNN, MVMS-net, MCA-net, EvoMBN, SPN-	12-lead EKG, continuous data	- MI Detection: 93.78% - MI Localization: 83.45%	- MI Detection: 93.78% - MI Localization : 83.45%

				V2, ASTL-Net, SSC-Net, ST-ReGE, SIGxCL, MSCT-Net			
Kumar et al.	2017	NA	Machine learning models: Random Forest (RF), J48 decision tree, Back Propagation Neural Network (BPNN), Least-Squares Support Vector Machine (LS-SVM)	- Random Forest (RF) - J48 decision tree - Back Propagation Neural Network (BPNN) - Least-Squares Support Vector Machine (LS-SVM)	Single-lead (lead-2) EKG	0.9931	NA
Pharvesh et al.	2024	NA	Deep learning models, specifically convolutional neural networks (CNNs), recurrent neural networks (RNNs), and attention-based models like ResNet34 with attention.	Convolutional Neural Networks (CNNs), Recurrent Neural Networks (RNNs), Attention-based models, ResNet34 with attention	12-lead EKG	0.922	- PTB-XL: 97.12% - PTB-XL (from Table IV): 97.17% - SPH: 99.40% - CPSC: 97.96%
Baglivo et al.	NA	NA	Deep learning models, specifically: - Multi-lead-fusion CNN - Multi-layer	Convolutional Neural Networks (CNNs), Recurrent Neural	12-lead EKG	- Precision: 96.59% - Recall: 98.96% - Average Accuracy: 97.67%	NA

			Perceptron (MLP) - Soft attention mechanism	Networks (RNNs), Generative Adversarial Networks (GANs), Long-Short Term Memory (LSTM) networks			
Elmannai et al.	2022	NA	- Deep Learning: Convolutional Neural Networks (CNNs) - Machine Learning: Decision Tree, K-nearest Neighbor, Support Vector Machine (SVM), Random Forest (RF), Logistic Regression (LR)	- Convolutional Neural Networks (CNNs): CNN-Model1, CNN-Model2, CNN-Model3 - Support Vector Machines (SVM) - Random Forests (RF) - K-Nearest Neighbors (KNN) - Logistic Regression (LR) - Decision Trees (DT)	Discrete heartbeat segments; lead type not specified	- MIT-BIH dataset: 99.8% - PTB dataset: 99.7%	NA
Li et al.	2024	NA	Deep learning models: MFB-SENET and MFB-DMIL; Multi-instance learning; DenseNet architecture	Convolutional Neural Networks (CNNs), specifically MFB-SENET and MFB-DMIL models; DenseNet architecture;	12-lead EKG	- PTB Database: Detection accuracy = 93.88%, Localization accuracy = 67.17% - PTB-XL Database: Detection	NA

				Multi-instance learning framework; Attention mechanism		accuracy = 94.89%, Localization accuracy = 85.83%	
Cao et al.	2020	NA	Deep learning (Convolutional Neural Networks), Machine learning	Convolutional Neural Networks (CNNs)	Multi-lead EKG (4 leads: v2, v3, v5, aVL)	0.9665	0.9671
Tripathy et al.	2019	NA	Deep learning (DNN), Machine learning (LSSVM, ANN, SVM)	- Deep Layer Least-Square Support Vector Machine (DL-LSSVM) - Artificial Neural Network (ANN) - Support Vector Machine (SVM)	12-lead EKG, continuous data	0.9974	NA
Zhang et al.	2019	NA	Deep learning, Neural networks (LSTM)	- Support Vector Machine (SVM) - Convolutional Neural Network (CNN) - Recurrent Neural Network (RNN) - Long Short Term Memory (LSTM)	8-lead EKG	0.9991	NA
Ma et al.	2021	NA	Deep learning, Neural networks	Convolutional Dendrite Net (CDD Net)	12-lead EKG, discrete data	0.9895	NA
Zhang et al.	2021 or later (inferre	NA	Deep learning, machine	Convolutional Neural	12-lead EKG	- Synthetic Experiments: r = 0.8624,	NA

	d from software versions)		learning, neural networks	Networks (CNNs)		RMSE = 0.1548, SSIM = 0.7988 - Clinical Experiments: Average Accuracy = 91.6 ± 9.8%	
Qu et al.	2024	NA	Deep learning, machine learning, neural networks	Convolutional Neural Networks (CNNs), multi-VGG DCNN, ML-ResNet, MFB-CBRNN, multi-branch densely connected convolutional network (MBDenseNet)	12-lead EKG, converted to 3-lead VCG	0.9411	NA
Ahmed et al.	2023	NA	- Deep learning: Residual-Dense Convolutional Neural Network (RD-CNN) - Machine learning: Linear Support Vector Machine (LSVM), AdaBoost, ANN, FisherFace, KNN, DecisionTree, NavieBayes, RandomForest	- Residual-Dense Convolutional Neural Network (RD-CNN) - Linear Support Vector Machine (LSVM) - AlexNet-SVM - CNN-filtering - SVM - CNN-LSTM - RNN-LSTM - DeepCNN - CNN-Pool - RD-CNN-AdaBoost -	- MIT-BIH Arrhythmia dataset: Two-channel ambulatory ECG recordings (continuous data) - PTB Diagnostic ECG Dataset: 12-lead ECG recordings (continuous data)	0.985	0.99

				RD-CNN-ANN - RD-CNN-FisherFace - RD-CNN-KNN - RD-CNN-DecisionTree			
Hammad et al.	2022	NA	Deep learning (Convolutional Neural Network - CNN), Machine learning (Support Vector Machine - SVM)	- Convolutional Neural Networks (CNNs) - Support Vector Machines (SVM) - K-nearest neighbor (KNN) - Recurrent Neural Networks (RNNs) - Residual networks - Capsule networks	12-lead EKG	- End-to-end CNN model: 98.90% - CNN with SVM classifier: 99.20%	NA
Sadad et al.	2023	NA	Deep learning (lightweight CNN with attention module)	Convolutional Neural Network (CNN)	12-lead EKG, continuous data	0.9839	NA
Zhang et al.	NA	NA	Deep learning (staked sparse autoencoder), Machine learning (bagged decision tree)	- Stacked Sparse Autoencoder (SAE) - TreeBagger classifier - Decision tree classifiers - Bagging algorithm	single-lead EKG (specifically lead II)	- MI Detection Accuracy: 99.90% - MI Localization Accuracy: 98.88%	NA

Jian et al.	2021	NA	Deep learning, specifically convolutional neural networks (CNN), including N-Net and MSN-Net models.	Convolutional Neural Networks (CNNs)	12-lead EKG	- MI Detection: 95.76% - MI Locating: 61.82%	NA
Liu et al.	NA	NA	Deep learning (DL), Transfer learning models (e.g., EfficientNetV2B 2)	Deep Representation Learning (DRL), EfficientNetV2B 2 (a type of CNN), Transfer Learning Models	12-lead EKG, discrete data	- For the MI class: 100% - Overall accuracy for normal vs. MI vs. other cardiac ailments: 99.03% (fivefold CV) - Highest overall accuracy: 99.92% (fivefold CV)	NA
Datta et al.	2022	NA	Machine learning (ML), Deep learning, Handcrafted models (specifically, multilevel hybrid feature extraction-based classification model), Shallow k-nearest neighbor (kNN) classifier	k-nearest neighbor (kNN) classifier	12-lead EKG	- Lead-wise accuracy: 99.85% (best in Lead III) - IMV accuracy: 99.94%	NA

Kora et al.	2015	NA	- Machine learning: Support Vector Machine (SVM) - Neural networks: Levenberg-Marquardt neural network (LM NN), Scalar Conjugate Gradient (SCG) NN - Nature-inspired optimization: Improved Bat Algorithm (IBA)	- Support Vector Machines (SVM) - K-Nearest Neighbors (KNN) - Levenberg-Marquardt Neural Network (LM NN) - Scalar Conjugate Gradient Neural Network (SCG NN)	Single-lead EKG (Lead III)	0.989	NA
Fang et al.	2022	NA	Deep learning models: multi-VGG deep neural network, VGG-19BN neural network with batch normalization; Machine learning: Grad-CAM ++ method	Convolutional Neural Networks (CNNs), specifically multi-VGG deep neural network with batch normalization and Grad-CAM ++ for interpretability	12-lead EKG	- PTB database: 95.65% inter-patient accuracy, 100% inner-patient accuracy - PTB-XL database: 97.23% inter-patient accuracy	- PTB database: 0.9861 - PTB-XL database: 0.9956
Deng et al.	2023	NA	Machine learning models: Naive Bayes classifier (NBC), Support vector machines (SVM), K-	- Naive Bayes classifier (NBC) - Support vector machines (SVM) - K-Nearest neighbor classifier (KNN)	12-lead EKG	0.984	NA

			Nearest neighbor classifier (KNN), Multilayer perceptron (MLP), Decision tree classifier (DTC); Neural networks: RBF neural networks	- Multilayer perceptron (MLP) - Decision tree classifier (DTC) - Convolutional Neural Networks (CNNs) (mentioned in comparisons) - Hill-climbing feature selection method			
Hernandez et al.	2023	NA	Deep learning, Recurrent Neural Networks (RNN), Long Short-Term Memory (LSTM) networks	Recurrent Neural Networks (RNNs)	Continuous 3-lead ECG data	- PPV: 100% - NPV: 94.73% (STAFF III test dataset) - NPV: 98.57% (PTB database - HC group) - NPV: 96.60% (PTB database - MI group) - NPV: 61.94% (PTB database - BBB group) - False positive rate: below 1%	NA
Chen et al.	2023	NA	Deep learning models: Convolutional Neural Networks (CNN),	- Classical methods: decision tree, support vector machines (SVM), Naive	- MIT-BIH Arrhythmia dataset: Two-channel, continuous data - PTB	- Physionet MIT-BIH dataset: 98.5% - PTB database: 98.28% - Normal class:	NA

			Recurrent Neural Networks (RNN), Long Short-Term Memory (LSTM)	Bayes Classification, multimodal feature fusion, multimodal image fusion (MIF), wavelet transform (WT), independent component analysis (ICA), interval information (RR), discrete cosine transform (DCT), Fisher's linear discriminant analysis - Deep learning models: convolutional neural networks (CNN), artificial neural networks (ANN), long short-term memory (LSTM) - Novel approach: evolving normalization, residual block, gradient clipping,	Diagnostics dataset: Single-lead (Lead II), continuous data - PhysioNet Challenge 2017 dataset: Continuous data	99.8% - Abnormal class: 97.7% - N class: 99% - Q class: 99% - V class: 97% - S class: 90% - F class: 88%	

				normalized gradient			
Chaitanya et al.	2024	NA	Machine learning models: DTE, NBS, KNN, SVM, ESSKNN; Ensemble learning	- Decision Trees (DTE) - Naive Bayes (NBS) - K-Nearest Neighbors (KNN) - Support Vector Machines (SVM) - Ensemble Subspace K-Nearest Neighbor (ESSKNN)	Vectorcardiogram (VCG) signals, segmented into four-second intervals	- 10-fold cross-validation: 99.97% - LOO cross-validation: 91.03% - IDCV: 99.41%	1
Jain et al.	2024	NA	Machine learning (logistic regression, ROCKET algorithm), Deep learning (convolutional neural network)	- Convolutional Neural Networks (CNNs) - Support Vector Machines (SVM) - Logistic Regression - Recurrent Neural Networks (RNNs) - Random Forests - Least Absolute Shrinkage and Selection Operator (LASSO) Regression	12-lead EKG, discrete data	NA	NA
Bellfield et al.	2024	NA	Convolutional neural networks	Convolutional Neural	12-lead EKG, discrete data	- For ECGs with 10 seconds of	- For ECGs with 10

			(CNN), specifically 2-D CNN models for image inputs and 1-D CNN models for signal inputs; machine learning with hyperparameter tuning	Networks (CNNs)	(10 seconds or 2.5 seconds per lead)	data: - Conservative cohort: Signal ECG AUC = 0.971 [0.961, 0.981], Extracted Signal ECG AUC = 0.974 [0.965, 0.984] - Speculative cohort: Signal ECG AUC = 0.931 [0.918, 0.945], Extracted Signal ECG AUC = 0.919 [0.903, 0.934] - For ECGs with 2.5 seconds of data: - Conservative cohort: Image ECG AUC = 0.960 [0.948, 0.973] - Speculative cohort: Image ECG AUC = 0.903 [0.886, 0.920]	seconds of data: - Conservativ e cohort: Signal ECG = 0.971 [0.961, 0.981], Extracted Signal ECG = 0.974 [0.965, 0.984] - Speculative cohort: Signal ECG = 0.931 [0.918, 0.945], Extracted Signal ECG = 0.919 [0.903, 0.934] - For ECGs with 2.5 seconds of data: - Conservativ e cohort: Image ECG = 0.960 [0.948, 0.973] - Speculative cohort: Image ECG =

							0.903 [0.886, 0.920]
Anw et al.	NA	NA	- Deep Learning: Deep Autoencoder (DAE) - Machine Learning: k-Nearest Neighbors (k-NN), Decision Tree (DT), Random Forest (RF), Support Vector Machine (SVM), Logistic Regression (LR), Naive Bayes	- k-NN - Decision Trees (DT) - Random Forests (RF) - Support Vector Machines (SVM) - Logistic Regression (LR) - Naive Bayes - Deep Autoencoder (DAE)	Single-lead EKG data from lead III	- Intrapatient paradigm: Accuracy = 99.74%, F1-score = 99.20% - Interpatient paradigm: Accuracy = 89.49%, F1-score = 89.2%	NA
Xu et al.	NA	NA	Deep learning (BiLSTM and LSTM networks)	- Bi-directional Long Short-Term Memory (BiLSTM) - Long Short-Term Memory (LSTM) - Support Vector Machines (SVM) - Artificial Neural Networks (ANN) - K-Nearest Neighbors (KNN)	12-lead EKG, single-lead EKG	0.9972	NA
Nadler et al.	2021	NA	Machine learning (ML), wavelet	- Support Vector Machines (SVMs) -	12-lead EKG	97.1% (on PTB database), 84.5% (on	0.99

			transforms, support vector machines (SVMs), ensemble learning (including random forest), deep learning	Ensemble Learning - Random Forests (RF) - XGBoost		independent test set), 90.80% (from cross-validation on clinical database)	
Shanmugam et al.	2018	NA	- Multiple Instance Learning (MIL) - Deep Learning - Convolutional Neural Networks (CNN) - Logistic Regression - Fully Connected Networks	Convolutional Neural Networks (CNNs), Logistic Regression, Support Vector Machines (SVM)	Continuous data, sampling rate of 128Hz, recorded over 48 consecutive hours	- 30 days: 0.83 ± 0.007 - 60 days: 0.79 ± 0.018 - 90 days: 0.81 ± 0.003 - 365 days: 0.78 ± 0.005	- 30 days: 0.83 ± 0.007 - 60 days: 0.79 ± 0.018 - 90 days: 0.81 ± 0.003 - 365 days: 0.78 ± 0.005
Chaitanya et al.	2023	NA	Machine learning models: Naive Bayes, Decision tree, K-nearest neighbor (KNN), Support vector machine (SVM), Ensemble subspace KNN; Optimization technique: Binary Harris hawk optimization (BHHO)	- Naive Bayes - Decision Tree - K-Nearest Neighbor (KNN) - Support Vector Machine (SVM) - Ensemble Subspace KNN	12-lead EKG, continuous or discrete data	- Class-oriented approach: 99.8% - Subject-wise strategy: 85.2%	NA

Lui et al.	2018	NA	- Convolutional Neural Network (CNN) - Recurrent Neural Network (RNN) - Long Short-Term Memory (LSTM) - Stacking Decoding	Convolutional Neural Networks (CNNs), Recurrent Neural Networks (RNNs) specifically Long Short-Term Memory (LSTM), Multi-Layer Perceptron (MLP)	Single-lead EKG data	- Sensitivity: 92.4% - Specificity: 97.7% - Positive Predictive Value: 97.2% - F1 Score: 94.6%	NA
Cao et al.	2014	NA	Machine learning models: SVM, Bayesian Logistic Regression, RBF Network, Ada Boost, Decision Tree; Fuzzy neural networks and block-based neural networks mentioned in related works	- Support Vector Machines (SVM) - Bayesian Logistic Regression - Radial Basis Function Network (RBF Network) - Adaptive Boosting (Ada Boost) - Decision Tree	NA	- Sensitivity improvement: 2% - Specificity improvement: 20.5% - Best sensitivity achieved: 0.753 (Ada boost model with ECG Codebook) - Best specificity achieved: 0.692 (RBF Network model with ECG Codebook) - SVM with ECG Codebook: 0.076 improvement	NA

						in sensitivity, 0.66 improvement in specificity	
Agrawal et al.	2022	NA	- Artificial Neural Networks (ANN) - Support Vector Machines (SVM) - Decision Trees (DT)	- Artificial Neural Networks (ANN) - Support Vector Machines (SVM) - Decision Trees (DT)	Vectorcardiography (VCG) data, specifically using XYZ leads and Vector Magnitude (VM) derived from it.	0.9831	0.998
Baloglu et al.	2019	NA	Deep learning, Convolutional Neural Network (CNN)	Convolutional Neural Networks (CNNs)	12-lead EKG	The outcome accuracy is over 99% for all ECG lead signals, with the highest accuracy being 99.78% for lead V4.	NA
Mahmoudinejad et al.	2021	NA	- Logistic Regression (LR) - Simple Decision Tree - Weighted K-nearest Neighbor (KNN) - Linear Support Vector Machine (SVM)	- Logistic Regression (LR) - Simple Decision Tree - Weighted K-nearest Neighbor (KNN) - Linear Support Vector Machine (SVM)	Multi-lead ECG data (at least 3 leads: lead I, lead II, and lead V2), likely discrete data	0.9037	NA
Boluda et al.	2022	NA	- Machine learning: Automated machine learning	- K-neighbors classifier - Extra trees classifier - GAMA (AutoML framework) -	single-lead ECG	- Multiclass problem: 98.30% - Binarization: 99.87%	NA

			methods, generalized island model with successive halving (GEISHA), extra trees classifier - Deep learning: Extreme learning machine (ELM), long short-term memory (LSTM), CNN, multilayer perceptron (MLP), transformers CNN, transformers LSTM	Extreme Learning Machine (ELM) - Long Short-Term Memory (LSTM) - Convolutional Neural Networks (CNN) - Multilayer Perceptron (MLP) - Transformers CNN - Transformers LSTM			
Herman et al.	NA	NA	Deep learning, neural networks	Convolutional Neural Networks (CNNs)	12-lead EKG, discrete data	90.9% (95% CI: 89.7–92.0)	0.938 (95% CI: 0.924-0.951)
Safdarian et al.	2021	NA	Machine learning (SVM classifier with linear, RBF, and polynomial kernels), Meta-heuristic optimization (Grasshopper Optimization Algorithm)	Support Vector Machines (SVM) with linear, RBF, and polynomial kernels	12-lead EKG	- MI Detection: 100% ± 0% - MI Classification (Polynomial Kernel): 94.2% ± 0.2%	NA

Chauhan et al.	2023	NA	- KNN (K-nearest neighbor) - SVM (support vector machine) - SAE-DNN (stacked autoencoder-based deep neural network)	- K-nearest neighbor (KNN) - Support vector machine (SVM) - Stacked autoencoder-based deep neural network (SAE-DNN)	12-lead EKG, continuous data	- For 30-minute ECG recordings: - MI detection: 98.84% (KNN), 98.27% (SVM), 98.27% (SAE-DNN) - MI localization: 86.64% (KNN), 83.17% (SVM), 81.98% (SAE-DNN) - For 4-second ECG recordings: - MI detection: 96.53% - MI localization: 93.32%	NA
Makimoto et al.	2020	NA	Convolutional Neural Network (CNN), Deep Learning	Convolutional Neural Networks (CNNs)	12-lead EKG	- CNN: F1 = 83 ± 4%, Accuracy = 81 ± 4% - Physicians: F1 = 70 ± 7%, Accuracy = 67 ± 7%	- Full: 0.88 ± 0.04 - Half: 0.87 ± 0.05 - Quarter: 0.87 ± 0.05 - Square: 0.85 ± 0.05
Kumar et al.	2021	NA	Deep learning: One-dimensional convolutional neural network (CNN); Potential future use: GoogLeNet, ResNet	- Convolutional Neural Networks (CNNs) - k nearest neighbor (KNN) - Support Vector Machines (SVM) - Tree-based algorithms	12-lead EKG, discrete data	0.8621	NA

Prati et al.	2022	NA	Deep learning, specifically Convolutional Neural Networks (CNN), particularly a 1D-CNN architecture	Convolutional Neural Networks (CNNs)	15-lead EKG (12 standard leads and 3 Frank leads)	The outcome accuracy is 99.98%.	NA
Zhang et al.	2019	NA	Deep learning (specifically, deep convolutional neural network)	Convolutional Neural Networks (CNNs)	12-lead EKG	- External validation: 91.0% - Comparative test: 90%	- External validation: 0.961 (95%CI, 0.933 to 0.989) - Comparativ e test: 0.955 (95%CI, 0.912 to 0.997)
Zhao et al.	2021	NA	Deep learning, Convolutional Neural Networks (CNNs), Long Short-Term Memory (LSTM) networks	Convolutional Neural Networks (CNNs), Long Short-Term Memory (LSTM) networks	12-lead EKG, continuous data	0.94	NA
Omar et al.	2020	NA	Deep learning models: 1D-CNN and Bi-LSTM	Convolutional Neural Networks (CNNs), Bidirectional Long Short-Term Memory (Bi-LSTM)	12-lead EKG, discrete data	- Training accuracy: 99.05% - Testing accuracy: 98.50%	NA

Shahnawaz et al.	2021	NA	Deep learning, Artificial Neural Network (ANN)	Artificial Neural Network (ANN)	Single-lead, continuous data	- MI Detection: 99.1% - Classification: 98.85%	NA
Sitaula et al.	2022	NA	Convolutional Neural Network (CNN), Deep Learning	Convolutional Neural Networks (CNNs)	12-lead EKG, discrete data	- Setting 1: MI detection accuracy = 99.82%, MI localization accuracy = 99.28% - Setting 2: MI detection accuracy = 93.93%, MI localization accuracy = 69.27%	NA
here et al.	2023	NA	Machine Learning (ML) techniques: K Nearest Neighbor (KNN), Support Vector Machine (SVM), Random Forest (RF)	K Nearest Neighbors (KNN), Support Vector Machine (SVM), Random Forest (RF)	Single-lead, continuous data	- KNN: 92.3% - SVM: 89.51% - RF: 90.91%	NA
Acharya et al.	2017	NA	Machine learning: k-Nearest Neighbors (KNN), Decision Tree (DT)	k-Nearest Neighbors (KNN), Decision Tree (DT)	12-lead EKG (from St. Petersburg database), single-lead (from Fantasia database), discrete data (sampled at 250	- KNN classifier with 13 bispectrum features: 98.17% - DT classifier with 31 cumulant features: 98.99%	NA

					and 257 samples per second)		
Herman et al.	NA	NA	NA	NA	12-lead EKG	- AUROC: 0.941 (95% CI: 0.926, 0.954) - Sensitivity: 82.6% (95% CI: 78.9%, 86.1%) - Specificity: 92.8% (95% CI: 91.5%, 93.9%) - PPV: 0.741 (95% CI: 0.7%, 0.778%) - NPV: 0.955 (95% CI: 0.945%, 0.965%)	0.941 (95% CI: 0.926, 0.954)
Tripathy et al.	2022	NA	- Machine learning (ML) - Deep learning (DL) - Convolutional neural networks (CNNs) - U-Net model architectures - SEResNet architecture - Rule-based AF detector	Convolutional Neural Networks (CNNs), U-Net models, SEResNet architecture, Rule-based algorithm	12-lead EKG	- MI detection 1D: Random weights (0.828283), WASP (0.848386), Enhanced WASP (0.883326) - AF detection 1D: Random weights (0.689362), WASP (0.831858), Enhanced	NA

						WASP (0.83004) - MI detection 2D: Random weights (0), Imagenet weights (0.862651), WASP (0.651869), Enhanced WASP (0.864383), Mixed modality model (0.760756) - AF detection 2D: Random weights (0), Imagenet weights (0.821429), WASP (0.531469), Enhanced WASP (0.846304), Mixed modality model (0.767329), Rule-based model (0.524871)	
Maria et al.	2018	NA	- Fully convolutional neural networks	Convolutional Neural Networks	12-lead EKG	- Sensitivity: 93.3% -	NA

			- Recurrent neural networks (LSTM) - Resnets	(CNNs), Recurrent Neural Networks (RNNs), specifically Long Short-Term Memory (LSTM) cells		Specificity: 89.7%	
Huong et al.	2024	NA	Deep learning (CNN-LSTM), Machine learning (PSO optimization)	- Convolutional Neural Networks (CNNs) - Long Short-Term Memory (LSTM) - Particle Swarm Optimization (PSO) - Support Vector Machine (SVM) - K-Nearest Neighbour (KNN) - Decision Tree (DT)	12-lead EKG	Lead I: Acc: 81.5 %, Sens: 57 %, Spec: 88.4 %, Prec: 58 %, BAcc: 73 %, F1: 57.5 % Lead II: Acc: 88 %, Sens: 64 %, Spec: 94 %, Prec: 76 %, BAcc: 79 %, F1: 69 % Lead III: Acc: 87 %, Sens: 84 %, Spec: 87 %, Prec: 65 %, BAcc: 86 %, F1: 74 % Lead IV: Acc: 85.2 %, Sens: 50.1 %, Spec: 90.5 %, Prec: 66.4 %, BAcc: 70 %, F1: 57.1 % Lead V: Acc: 85.6 %, Sens: 50.1 %, Spec: 96 %,	NA

						Prec: 76.3 %, BAcc: 72.8 %, F1: 60.5 % Lead VI: Acc: 87.7 %, Sens: 80 %, Spec: 90 %, Prec: 69 %, BAcc: 85 %, F1: 74 % Score Fusion: Acc: 92 %, Sens: 80 %, Spec: 94 %, Prec: 81 %, BAcc: 88 %, F1: 81 %	
Jahmunah et al.	2021	NA	Deep learning models: Convolutional Neural Network (CNN), GaborCNN model	Convolutional Neural Networks (CNNs), GaborCNN model	Single-lead (Lead II)	- CNN model: 99.55% - GaborCNN model: 98.74%	NA
Kumar et al.	2022	NA	- Artificial Neural Network (ANN) - Support Vector Machine (SVM)	- Artificial Neural Network (ANN) - Support Vector Machine (SVM)	Single-lead, continuous data	- ANN: 100% for CAD and MI vs. controls, 99.0% for CAD vs. MI - SVM: 99.6% for CAD and MI vs. controls, 99.3% for CAD vs. MI	NA
Pan et al.	2022	NA	Deep learning models, specifically a multi-task	- Traditional machine learning methods: SVM,	12-lead EKG	- MI Detection: More than 90% accuracy on both PTB and	- Location-5: 78.76% - Location-7: 74.05%

			channel attention network (MCA-net) with a residual structure, Convolutional Neural Network (CNN), and Long Short-Term Memory (LSTM).	KNN, Random Forests, HMM - Deep learning methods: CNNs, GANs, RNNs		PTBXL datasets. - MI Location on PTB: 68.90% and 49.18% accuracy. - MI Location on PTBXL: More than 80% accuracy.	
Sugimoto et al.	2019	NA	Deep learning (Convolutional Autoencoder), Machine learning (k-Nearest Neighbor classifier)	Convolutional Autoencoder (CAE), k-Nearest Neighbor (k-NN)	12-lead EKG, discrete data	0.9987	NA
Sharma et al.	2018	singapore	Machine learning: k-nearest neighbor (KNN)	k-nearest neighbor (KNN)	Single-lead EKG data	- Noisy dataset: 99.62% - Clean dataset: 99.74%	1
Cho et al.	2022	South Korea	Deep learning, specifically using a convolutional neural network (CNN) architecture with a multi-channel CNN network and a non-local network block.	Convolutional Neural Networks (CNNs)	12-lead ECG images	- Area under the curve (AUC): 0.947 - Sensitivity: 98.1% (95% CI, 94.6%, 100.0%) - Specificity: 76.9% (95% CI, 60.7%, 93.1%) - Positive	0.947

						Predictive Value (PPV): 89.8% (95% CI, 82.1%, 97.5%) - Negative Predictive Value (NPV): 95.2% (95% CI, 86.1%, 100.0%)	
Campuzano et al.	2022	South Korea	Deep learning model (DLM)	Convolutional Neural Networks (CNNs), specifically a deep learning model (DLM) based on ResNet structure	12-lead EKG, discrete data	- AUROC: 0.998 (0.996-0.999) - Sensitivity: 97.4% (95.7-100) - Specificity: 99.2% (98.1-99.4) - NPV: 99.9% - PPV: 20.2%	- Overall STEMI: 0.998 (0.996-0.999) - RCA: 0.998 (0.995-0.999) - LAD: 0.998 (0.996-0.999) - LCX: 0.999 (0.998-1.000)
Won et al.	2023	South Korea	- Deep Learning (DL) models: - ResNet - 1D ResNet - Bi-LSTM - 1D CNN - MultiHeadAttention - Machine Learning (ML) models: - Logistic	- ResNet - Logistic Regression (LR) - Random Forest (RF) - Long Short Term Memory (LSTM) - Transformer	12-lead EKG	- Obstructive coronary artery disease (ObCAD): 0.638 - Acute myocardial infarction (AMI): 0.885	- ObCAD: 0.693 - AMI: 0.923

			Regression (LR) - Random Forest (RF) - Long Short Term Memory (LSTM) - Transformer				
Lee et al.	2024	South Korea	Deep learning, specifically a deep ensemble model combining 5 convolutional neural networks.	Convolutional Neural Networks (CNNs)	12-lead EKG	- Test set: Accuracy = 92.1%, Sensitivity = 95.4%, Specificity = 91.8% - Clinical validation set: Accuracy = 89.3%, Sensitivity = 95.0%, Specificity = 89.1% - External validation set: Accuracy = 97.6%, Sensitivity = 83.3%, Specificity = 97.9%	- Test set: AUROC of 0.981 - Clinical validation set: AUROC of 0.978 - External validation set: AUROC of 0.979
Han et al.	NA	South Korea	Deep learning, neural networks	Convolutional Neural Networks (CNNs), Residual Networks (ResNet), Multi-	Asynchronous ECG lead sets derived from standard 12-lead ECG reports, including 12-	- AUROC values: - 12-lead set: 0.880 - 4-lead sets: 0.858 (SD 0.008) - 3-lead sets: 0.845 (SD	- 12-lead set: AUROC 0.880 - 4-lead sets: AUROC 0.858 (SD 0.008) - 3-

				head Self-Attention Module	lead, 4-lead, 3-lead, 2-lead, and single-lead sets.	0.011) - 2-lead sets: 0.813 (SD 0.018) - Single-lead sets: 0.768 (SD 0.001) - AUPRC values: - 12-lead set: 0.314 - 4-lead sets: 0.225 (SD 0.011) - 3-lead sets: 0.210 (SD 0.020) - 2-lead sets: 0.171 (SD 0.020) - Single-lead sets: 0.138 (SD 0.014) - Gains in sensitivity: - At specificity=0.866: - 12-lead set: +13.9% - 4-lead sets: +10.2% (SD 1.6%) - 3-lead sets: +8.5% (SD 2.7%) - At specificity=0.647: - 12-lead set: +11.9% - 4-lead sets: +9.8% (SD 1.2%) - 3-lead sets: +8.1% (SD 1.5%)	lead sets: AUROC 0.845 (SD 0.011) - 2-lead sets: AUROC 0.813 (SD 0.018) - Single-lead sets: AUROC 0.768 (SD 0.001)
Gustafsson et al.	2022	Sweden	Deep learning, specifically	Convolutional Neural	12-lead EKG	- C-statistic for STEMI: 0.991	- Random test set:

			convolutional neural networks (CNNs) and ensembles of neural networks	Networks (CNNs), specifically Residual Networks (ResNet)		(random test set), 0.985 (temporal test set) - C-statistic for NSTEMI: 0.832 (random test set), 0.867 (temporal test set) - Brier score for STEMI: 0.001 (random test set), 0.002 (temporal test set) - Brier score for NSTEMI: 0.008 (random test set)	STEMI vs. control = 0.991, NSTEMI vs. control = 0.832 - Temporal test set: STEMI vs. control = 0.985, NSTEMI vs. control = 0.867 - External test set: STEMI vs. control = 0.985
Liu et al.	2021	Taiwan	Deep learning model (DLM) based on ECG12Net	Convolutional Neural Networks (CNNs)	12-lead EKG	- STEMI detection: AUC=0.976 (human-machine competition), AUC=0.997 (independent use); Sensitivity=98.4%, Specificity=96.9% - NSTEMI detection: AUC=0.978 (combined	- STEMI detection by DLM: 0.997 - NSTEMI detection by DLM and cTnI: 0.978

						with cTnI); Sensitivity=91.6%, Specificity=96.7%	
Kavak et al.	2022	Taiwan	- Primary AI model: 2D-CNN (Convolutional Neural Network) - Transfer learning models: VGG, ResNetV2, Xception, InceptionV3, InceptionResNetV2, Mobile-NetV2, DenseNet	Convolutional Neural Networks (CNNs), specifically the proposed 2D-CNN model and various transfer learning models including VGG, ResNetV2, Xception, InceptionV3, InceptionResNetV2, Mobile-NetV2, and DenseNet.	12-lead EKG	0.963	0.962
Tseng et al.	2022	Taiwan	Deep learning, Convolutional Neural Network (CNN), Neural Networks	Convolutional Neural Network (CNN)	12-lead EKG, continuous data	- LAD: Sensitivity 85.7%, Specificity 88.4% - LCX: Sensitivity 37%, Specificity 99% - RCA: Sensitivity 88.4%, Specificity 82.4% - Overall Accuracy with	NA

						STFT: 79.3% - Overall Accuracy with CWT: 83.7%	
Lin et al.	2023 or 2024 (inferred based on trial dates)	Taiwan	Deep learning, machine learning, neural networks	NA	12-lead EKG	- Positive Predictive Value: 89.5% (95% CI, 85.3 to 93.6%) - Negative Predictive Value: 99.9% (95% CI, 99.9 to 100.0%) - Sensitivity: 89.5% (95% CI, 85.3 to 93.6%) - Specificity: 99.9% (95% CI, 99.9 to 100.0%)	NA
Alizadehsani et al.	NA	Taiwan	Deep learning (CNN-LSTM)	Convolutional Neural Network (CNN) and Long Short-Term Memory (LSTM)	12-lead EKG, discrete data	- Accuracy: 0.992 - Precision: 0.889 - Specificity: 0.994 - Recall: 0.941 - Area under the receiver operating characteristic curve: 0.997 - F1 score: 0.914	0.997

Chumachenko et al.	2022	Ukraine	Machine learning models: k-nearest neighbor classifier, radial basis function, decision tree, random forest	- K-nearest neighbor classifier - Radial basis function - Decision tree - Random forest	12-lead EKG, discrete data (10-second recordings)	99.63% (or 99.629%) for the optimized Random Forest model	NA
Lujain et al.	2020	United Arab Emirates	- Convolutional Neural Network (CNN) - Recurrent Neural Network (RNN) - Decision-tree based model: XGBoost	- Convolutional Neural Network (CNN) - Recurrent Neural Network (RNN) - XGBoost (decision-tree based model)	12-lead EKG	- CNN: 89.9% - RNN: 84.6% - XGBoost: 97.5%	- CNN: 90.7% - RNN: 82.9% - XGBoost: 96.5%
Garvey et al.	2016	United States	NA	NA	12-lead EKG	- Sensitivity: AG1 = 0.69, AG2 = 0.68, AG3 = 0.62 - Specificity: AG1 = 0.89, AG2 = 0.91, AG3 = 0.95 - A-ROC for culprit artery: 0.78-0.79 - A-ROC for STEMI activation: 0.82-0.87	- For culprit artery identification: 0.78-0.79 - For STEMI activation: 0.82-0.87
Rashid et al.	2020	United States	Binary Convolutional Neural Network (BCNN)	- Convolutional Neural Networks (CNNs) -	Single-lead EKG data (Lead 11)	0.9029	NA

				Support Vector Machines (SVMs) - Random Forests - Binary Convolutional Neural Network (BCNN) - k-Nearest Neighbor (k-NN)			
Al-Zaiti et al.	2020	United States	- Logistic Regression (LR) - Gradient Boosting Machine (GBM) - Artificial Neural Network (ANN)	- Logistic Regression (LR) - Gradient Boosting Machine (GBM) - Artificial Neural Network (ANN) - Support Vector Machines (SVM) - Naive Bayes - Random Forest	12-lead EKG, discrete data	- Predicting any ACS event: - Sensitivity: 0.77 (0.67–0.85) - Specificity: 0.76 (0.72–0.81) - PPV: 0.43 (0.38–0.48) - NPV: 0.94 (0.91–0.96) - Predicting NSTE-ACS events: - Sensitivity: 0.72 (0.60–0.81) - Specificity: 0.76 (0.72–0.80) - PPV: 0.36 (0.31–0.41) - NPV: 0.94 (0.91–0.93)	NA

Panchal et al.	2020	United States	Deep learning (1-D convolutional neural network)	Convolutional Neural Network (CNN)	NA	- TLF: 8.50% vs. 16.50% (p = 0.004) - Cardiac death: 0.98% vs. 1.96% (p = 0.451) - TV-MI: 4.68% vs. 12.44% (p < 0.001) - Clinically driven target lesion revascularizati on: 4.40% vs. 7.46% (p = 0.129) - Periprocedural TV-MI: 3.21% vs. 7.46% (p = 0.024) - Spontaneous TV-MI: 1.48% vs. 5.00% (p = 0.015) - Definite/proba ble ST: 0.49% vs. 2.00% (p = 0.096) - Late/very late ST: 0% vs. 1.87% (p = 0.012)	NA
Sharma et al.	2020	United States	Machine learning (k-	k-Nearest Neighbors (kNN)	Single-lead ECG data (lead V5 for detection,	- MI detection using 12 top-ranked	- MI detection using 12

			Nearest Neighbors)		lead V3 for localization)	features: 99.00% - MI detection using features from lead V5 only: 99.05% - MI localization using 5 top-ranked features: 99.76% - MI localization using features from lead V3 only: 99.28%	top-ranked features: AUC = 0.99 - MI detection using lead V5 only: AUC = 0.99 - MI localization using 5 top-ranked features: AUC = 0.99 - MI localization using lead V3 only: AUC = 0.98
Zhang et al.	2021	United States	Deep learning, Machine learning (specifically XGBoost and other ensemble methods like AdaBoost, GBDT, Bagging, Random Forest, ExtraTrees)	- Convolutional Neural Networks (CNNs) - eXtreme Gradient Boosting (XGBoost) - K-Nearest Neighbor (KNN) - Gaussian Naive Bayes (GNB) - Linear Discriminant Analysis (LDA) - Decision Tree (DT) - Support Vector Machine	12-lead EKG, discrete data	0.9986	NA

				(SVM) - Logistic Regression (LR) - Random Forest - ExtraTrees - AdaBoost - Bagging - GBDT			
Bouzid et al.	2022	United States	Machine learning (random forest classifier)	Random Forests	12-lead EKG	- Confirmed acute coronary syndrome: 288 (13.6%) - Final discharge diagnosis of NSTE-ACS: 179 (8%) - Subsequent inhospital evolution of STE-ACS: 109 (5%)	- Baseline AUC for expert interpretati on of out-of-hospital ECG: 0.69 - AUC for serial ECG changes: 0.80 - AUC for artificial intelligence-augmented out-of-hospital ECG: 0.83
Mclaren et al.	2022	United States	Deep learning, Neural networks	NA	12-lead EKG, serial ECGs, continuous monitoring	- Sensitivity of Dr. Smith: 90%, 86%, 79% - Sensitivity of STEMI Criteria: 44%, 41%, 36% - Specificity of Dr. Smith: 88%, 91%, 93% -	NA

						Specificity of STEMI Criteria: 94%, 94%, 95% - Accuracy of Dr. Smith: 89%, 89%, 88% - Accuracy of STEMI Criteria: 79%, 77%, 72%	
Xiao et al.	2023	United States	Deep learning, specifically using a novel multimodal deep learning architecture and a deep neural network (xResNet)	Convolutional Neural Networks (CNNs), specifically a variant of ResNet (xResNet)	12-lead EKG, discrete data (10 s waveforms)	0.874	0.921
Yao et al.	2023	United States	Deep learning models: Convolutional Neural Networks (CNNs), Residual Network with SE blocks, Transformer encoder; Machine learning: Feature extraction and classification;	- Convolutional Neural Networks (CNNs) - Transformer encoder - Residual network with SE blocks - Customized Pooling Component (CPC)	12-lead EKG	0.83	0.76868

			Natural Language Processing (NLP): Transformer encoder				
Karnewar et al.	2023	United States	Machine learning (K-nearest neighbor (kNN) clustering, decision tree classifier with entropy criterion)	K-nearest neighbor (kNN), Decision Tree (Entropy)	Continuous, two-channel EKG data	- MIT-BIH Arrhythmia dataset: 87.5% - BIDMC CHF dataset: 99.33%	NA
Ul et al.	2024	United States	- Deep Learning: Deep Residual CNN - Machine Learning: k-NN, SVM, Tree, Bagged, Naïve Bayes, Ensemble - Bayesian Optimization: Used for hyperparameter optimization	- Convolutional Neural Networks (CNNs): Deep Residual CNN - Support Vector Machines (SVM) - k-NN - Tree - Naive Bayes - Ensemble	NA	1	NA
Kim et al.	2024	United States	- Deep learning: Deep Convolutional Neural Network (CNN) - Machine learning: Light Gradient Boost	Convolutional Neural Networks (CNNs), Light Gradient Boost Machine (LightGBM)	Nocturnal single-lead ECG	0.99387	NA

			Machine (LightGBM)				
authors) et al.	NA	United States	Machine learning models including regularized logistic regression, linear discriminant analysis, support vector machine, Gaussian Naïve Bayes, random forest, gradient boosting machine, extreme gradient boosting, stochastic gradient descent logistic regression, k-nearest neighbors, and artificial neural networks.	- Regularized logistic regression - Linear discriminant analysis - Support vector machine - Gaussian Naïve Bayes - Random forest - Gradient boosting machine - Extreme gradient boosting - Stochastic gradient descent logistic regression - K-nearest neighbors - Artificial neural networks	12-lead EKG	- Sensitivity: 0.86 (95% CI 0.81-0.91) - Specificity: 0.98 (95% CI 0.97-0.99) - PPV: 0.54 (95% CI 0.46-0.62) - NPV: 0.99 (95% CI 0.98-0.99)	NA
Yildirim et al.	NA	United States	Deep Neural Network (DNN)	Deep Neural Network (DNN)	12-lead EKG, discrete data collected for 10 seconds at 500Hz sampling frequency	- Training accuracy: >98% - Validation accuracy: 71 ± 5% - AUC-ROC: 0.730	0.73

Jegan et al.	2016	United States (implied by the reference to the University of California, Irvine)	- Feed Forward Neural Network (FFNN) - Cascade Correlation Neural Network (CNN) - Support Vector Machine (SVM) - Genetic Optimized Neural Network (GAANN) - Particle Swarm Optimized Neural Network (PSONN)	Feed Forward Neural Network (FFNN), Cascade Correlation Neural Network (CNN), Support Vector Machine (SVM)	NA	- CNN: 85.6% - FFNN: 81.2% - SVM: 79% - GAANN: 85.9% and 88.5% - PSONN: 89.61% (Cleveland), 85.62% (clinical)	NA
Holst et al.	NA	United States (inferred from the mention of Bowman Gray School of Medicine Data Base and AHA specifications)	Neural networks (specifically, multi-layer perceptron architecture), machine learning (modified k-means clustering technique)	Artificial Neural Network (multi-layer perceptron architecture), Modified k-means clustering technique	12-lead EKG, discrete data	- Overall ROC area: 0.887 - ROC area for 25% with lowest error estimates: 0.995 - Total accuracy: 83.7% - Accuracy excluding high/low probability estimates: 83.8%	0.887 (0.845-0.922)
Odema et al.	2021	United States of America	- Primary model: Binary Convolutional Neural Networks	- Convolutional Neural Networks (CNNs) - Support Vector	Single-lead EKG data	0.9122	NA

			(BCNNs) - Related works: Support Vector Machine (SVM), Random Forest (RF), Convolutional Neural Networks (CNNs)	Machines (SVM) - Random Forests (RF) - Binary Convolutional Neural Networks (BCNN)			
Camara et al.	NA	United States, Germany, United Kingdom	- Deep learning networks - Conditional generative model - Variational autoencoders (VAEs) - Generative adversarial networks (GANs) - Cycle-consistent GAN (CycleGAN) - Encoder-decoder architecture - Condition mapping network - Discriminator networks - PyTorch framework	- Generative model for heart ageing synthesis - Biomechanical regularization for displacement vector field estimation - PyTorch for model implementation - Adam optimizer for training	NA	The outcome accuracy is implied by the model's performance in predicting realistic anatomical changes and its comparison to other models, with metrics such as Dice score and Hausdorff distance indicating high accuracy. Specific values are provided in the tables referenced.	- For 3D shape inputs: ~16% improvement over clinical benchmarks - For 3D contraction inputs: ~6% improvement over clinical benchmarks
Liu et al.	2018	Unkown	Deep learning, Convolutional	- Convolutional Neural	Multilead EKG (using leads V2,	0.96	NA

			Neural Network (CNN), Neural Networks	Networks (CNNs) - Support Vector Machines (SVM) - Neural Networks (NN) - K-Nearest Neighbors (KNN) - Decision Trees - Random Forests (RF)	V3, V5, and aVL), continuous data		
Prabhakararao et al.	2020	unkown	Deep learning, specifically recurrent neural networks (RNNs) with intra-and inter-lead attention modules	- Deep Convolutional Neural Network (DCNN) - Multiple-feature-branch CNN (MFB-CNN) - MFB-CRNN (MFB-CNN followed by bi-directional recurrent neural network) - Multi-lead residual neural network (ML-ResNet) - Recurrent Neural Network (RNN) with intra-and inter-lead attention modules	12-lead EKG	0.983	0.987±0.004

Liu et al.	2020	Unkown	Deep learning models: Convolutional Neural Network (CNN), Recurrent Neural Network (RNN), Bidirectional Long Short-Term Memory (BLSTM); Machine learning algorithms: conventional ML-based schemes	- Convolutional Neural Networks (CNNs) - Recurrent Neural Networks (RNNs) - Bidirectional Long Short Term Memory (BLSTM) - Support Vector Machines (SVM) - K-Nearest Neighbor (KNN) - Multilayer Perceptron (MLP)	12-lead EKG	- Class-based accuracy: 99.90% - Subject-based accuracy: 93.08%	NA
Sinha et al.	2021	Unkown	Machine learning: Support Vector Machines (SVM), Boosting framework with ensemble of SVM classifiers, Clustering-based training sample set selection algorithm	- Boosted Support Vector Machine (SVM) - Adaboost - Single SVM - K-Nearest Neighbors (KNN) - Ensemble classification with majority voting	12-lead EKG, discrete data	- Detection accuracy: 98.96% - Identification accuracy: 98.85%	NA
Jafarian et al.	2020	USA	- Classic approach: Shallow neural networks (NN) -	- Classic approach: Discrete Wavelet	12-lead EKG	- Classic approach: Over 98% accuracy - End-to-end	NA

			End-to-end approach: Convolutional Neural Network (CNN) with deep residual learning	Transform (DWT), Principal Component Analysis (PCA), Shallow Neural Networks (NN) - End-to-end approach: Convolutional Neural Networks (CNNs) with deep residual learning and dilated convolutions		CNN model: 100% accuracy	
Martin et al.	2021	USA	Long Short-Term Memory Neural Network (LSTM), Recurrent Neural Networks (RNNs), Deep Learning	- Convolutional Neural Networks (CNNs) - Support Vector Machines (SVM) - Random Forests - Hybrid Firefly algorithm - k-nearest neighbor classifier - Bagging tree classifier - Multi-branch fusion network - Long Short-Term Memory	Single-lead EKG (Lead II)	89.56% (with a 95% Confidence Interval of ±2.79%)	NA

				(LSTM) neural network			
Muminov et al.	2020	Uzbekistan	Convolutional Neural Network (CNN), Deep Learning	Convolutional Neural Network (CNN)	12-lead EKG	0.9847	NA
Yousuf et al.	2024	Mulitcountry	NA	2-D convolutional neural network (CNN),equivalent dataset of grayscale images using Gramian angular summation field (GASF) and Gramian angular difference field (GADF) operations	lead-II of the ECG data	99.68%, 99.80%, 99.82%, and 99.84% under GASF dataset with noise and baseline wander, GADF dataset with noise and baseline wander, GASF dataset with noise and baseline wander removed, and GADF dataset with noise and baseline wander removed, respectively.	NA
ZHANG et al.	2019	China	deep learning networks	staked sparse autoencoder (SAE) and the bagged decision tree (TreeBagger)	single-lead	0.999	NA

Anwar et al.	2024	india	NA	autoencoder-k-NN classifier	NA	0.9974	NA
Bhaskarpandit et al.	2023	India	NA	eigendomain-based deep representation learning (DRL) approach	12-lead ECG	99.03	NA
Chang et al.	2021	NA	NA	long short-term memory (LSTM) model	NA	0.987	0.997
Chen et al.	2022	Taiwan	NA	convolutional neural network and long short-term memory (CNN-LSTM)	12-lead ECG	0.992	0.997
Dey et al.	2021	Bangladish	NA	one dimensional (1-D) convolutional neural network (CNN) and a bidirectional long short-term memory (bi-LSTM) layer	12-lead ECG	0.99246	NA
al et al.	2022	Unkown	CNN	NA	NA	0.92	NA
Herman et al.	2023	multicountries	NA	NA	12-lead	0.909	0.938
NA	NA	NA	NA	NA	NA	NA	NA
Jahmunah et al.	2023	Mulitcountry	NA	Dirichlet DenseNet model	lead V6	80	NA
Jiang et al.	2024	China	deep learning; electrocardiogram	CNN	NA	97.67	NA

Kolliyil et al.	2024	NA	NA	temporal projection peak of the wavelet transform to assess the QRS complex strength. Binary classification models for each ECG class were built using eXtreme Gradient Boosting (XGBoost).	NA	NA	0.86
Liang et al.	2024	China	NA	tree method with a joint analysis of static and dynamic features	NA	97.1% accuracy under the inter-patient framework on the PTB database. Clinical database, resulting in an accuracy of 84.5%.	NA
Mehta et al.	2020	Unknown	A 1-D convolutional neural network was implemented	NA	Single lead	NA	NA
Mehta et al.	2020	Unknown	1-D convolutional neural network	NA	12 leads	0.924	NA

Mehta et al.	2020	Unknown	NA	NA	Artificial Intelligence (AI)–augmented EKG	98.1	NA
Mishra et al.	2024	Unknown	deep learning algorithm	convolutional neural networks (CNN)	NA	97.86% for multiclass classification, Binary detection yielded for MI	NA
Lin et al.	2024	Taiwan	NA	NA	12-lead electrocardiograms	NA	NA
Dandapat et al.	NA	India	NA	NA	2-D multilead ECG	0.953	NA
Parmar et al.	2024	NA	NA	NA	VLSI architecture	0.999	NA
Parupudi et al.	2023	NA	smartphone-enabled deep learning (DL)-	five branches of network layers, with each branch consisting of a frozen transfer learner block followed by global average pooling, batch normalization, drop-out, and Softmax layers	12-lead ECG	0.9934	NA
Sbrollini et al.	2021	NA	NA	neural networks (NN) alternating structuring: maximal	NA	NA	92% were characterized by AUC≥80%

				number of layers (NL), maximal number of initializations (NI) and maximal number of confirmations (NC)			and 31% by AUC≥85%.
yet et al.	NA	NA	NA	NA	NA	NA	NA
Zhu et al.	2021	China and Kenya	Machine learning	end-to-end deep learning approach, DeepMI	12-lead ECG	NA	0.829
Wu et al.	2022	China	NA	denoising and segmentation modules	NA	NA	NA

Abbreviations: AI – Artificial Intelligence; ECG – Electrocardiogram; MI – Myocardial Infarction; CNN – Convolutional Neural Network; AUC – Area Under the ROC Curve.
