# Supplementary material for: Artificial Intelligence for Myocardial Infarction Detection via Electrocardiogram: A Scoping Review
Source: J Clin Med. 2025 Sep 25;14(19):6792. doi: 10.3390/jcm14196792 (PMC12525322; doi:10.3390/jcm14196792)
Supplement: Supplementary file 1 [file jcm-14-06792-s001.zip › References list.pdf]

Artificial Intelligence for Myocardial Infarction Detection via Electrocardiogram: A Scoping Review

Supplementary material

Title	Reference
Evolution of single-lead ECG for STEMI detection using a deep learning approach	(1)
Multi-Channel Lightweight Convolution Neural Network for Anterior Myocardial Infarction Detection	(2)
Multi-branch fusion network for Myocardial infarction screening from 12-lead ECG images	(3)
Myocardial Infarction Detection Based on Multi	(4)
Automated detection of cardiovascular disease by electrocardiogram signal analysis: a deep learning system	(5)
Early detection of ST-segment elevated myocardial infarction by artificial intelligence with 12-lead electrocardiogram	(6)
Hybrid Network with Attention Mechanism for Detection and Location of Myocardial Infarction Based on 12-Lead Electrocardiogram Signals	(7)
A dynamic learning-based ECG feature extraction method for myocardial infarction detection	(8)
An ECG generative model of myocardial infarction	(9)
Automated localization and severity period prediction of myocardial infarction with clinical interpretability based on deep learning and knowledge graph	(10)
Automated Localization of Myocardial Infarction of Image-Based Multilead ECG Tensor With Tucker2 Decomposition	(11)
Deep Learning Networks Accurately Detect ST-Segment Elevation Myocardial Infarction and Culprit Vessel	(12)
High performance of privacy-preserving acute myocardial infarction auxiliary diagnosis based on federated learning: a multicenter retrospective study	(13)
ECG detection of ACS by convolutional neural network utilizing transfer learning	(14)
LASSO Regression-Based Diagnosis of Acute ST-Segment Elevation Myocardial Infarction (STEMI) on Electrocardiogram (ECG)	(15)
The feasibility of early detecting coronary artery disease using deep learning-based algorithm based on electrocardiography	(16)
Artificial intelligence-assisted remote detection of ST-elevation myocardial infarction using a mini--lead electrocardiogram device in prehospital ambulance care	(17)
Implementation of an All-Day Artificial Intelligence-Based Triage System to Accelerate Door-to-Balloon Times	(18)

DeepMI: Deep multi-lead ECG fusion for identifying myocardial infarction and its occurrence-time	(19)
Enhanced Automated Diagnosis of Coronary Artery Disease Using Features Extracted From QT Interval Time Series and ST-T Waveform	(20)
A Simple and Effective Method for Detecting Myocardial Infarction Based on Deep Convolutional Neural Network	(21)
Automated Detection of Myocardial Infarction Using a Gramian Angular Field and Principal Component Analysis Network	(22)
Automated interpretable detection of myocardial infarction fusing energy entropy and morphological features	(23)
Myocardial Infarction Classification Based on Convolutional Neural Network and Recurrent Neural Network	(24)
Application of multi-feature fusion and random forests to the automated detection of myocardial infarction Action editor	(25)
Classification of myocardial infarction based on hybrid feature extraction and artificial intelligence tools by adopting tunable-Q wavelet transform (TQWT), variational mode decomposition (VMD) and neural networks	(26)
A novel network to detect and locate myocardial infarction using 12 leads ECG	(27)
A multi-dimensional association information analysis approach to automated detection and localization of myocardial infarction	(28)
Localization of myocardial infarction with multi-lead ECG based on DenseNet	(29)
A lightweight and updatable myocardial infarction diagnosis system based on convolutional neural networks and active learning	(30)
ML-Net: Multi-Channel Lightweight Network for Detecting Myocardial Infarction	(31)
Discrimination of different myocardial infarction stages using wide band electrocardiogram	(32)
Multiple-feature-branch convolutional neural network for myocardial infarction diagnosis using electrocardiogram	(33)
Acute Myocardial Infarction Detection Using Deep Learning-Enabled Electrocardiograms	(34)
Detection of myocardial infarction using Shannon energy envelope, FA-MVEMD and deterministic learning	(35)
Real-time frequency-independent single-Lead and single-beat myocardial infarction detection ☆	(36)
Deep Learning for Detecting and Locating Myocardial Infarction by Electrocardiogram: A Literature Review	(37)
Intelligent Recognition Algorithm of Multiple Myocardial Infarction Based on Morphological Feature Extraction	(38)
Localization of myocardial infarction using a multi-branch weight sharing network based on 2-D vectorcardiogram	(39)

Myocardial infarction detection using ITD, DWT and deterministic learning based on ECG signals	(40)
Detection of inferior myocardial infarction based on multi branch hybrid network	(41)
SRTNet: Scanning, Reading, and Thinking Network for myocardial infarction detection and localization	(42)
An SNN-Inspired Area-and Power-Efficient VLSI Architecture of Myocardial Infarction Classifier for Wearable Devices	(43)
Automated detection of myocardial infarction based on an improved state refinement module for LSTM/GRU	(44)
Automatic detection and localisation of myocardial infarction using multi-channel dense attention neural network	(45)
Localization of Myocardial Infarction With Multi-Lead Bidirectional Gated Recurrent Unit Neural Network	(46)
Myocardial Infarction Detection and Localization with Electrocardiogram Based on Convolutional Neural Network	(47)
A novel myocardial infarction localization method using multi-branch DenseNet and spatial matching-based active semi-supervised learning	(48)
Application of deep convolutional neural network for automated detection of myocardial infarction using ECG signals	(49)
Automated Localization of Myocardial Infarction From Vectorcardiographic via Tensor Decomposition	(50)
ST-Net: Synthetic ECG tracings for diagnosing various cardiovascular diseases	(51)
Explainable detection of myocardial infarction using deep learning models with Grad-CAM technique on ECG signals	(52)
Automated detection of myocardial infarction from ECG signal using variational mode decomposition based analysis	(53)
Convolutional Neural Networks Based Diagnosis of Myocardial Infarction in Electrocardiograms	(54)
ECG based Myocardial Infarction detection using Hybrid Firefly Algorithm	(55)
Inferior myocardial infarction detection using stationary wavelet transform and machine learning approach	(56)
Myocardial Infarction Severity Stages Classification From ECG Signals Using Attentional Recurrent Neural Network	(57)
Autonomic Features in Prediction of Coronary Artery Disease and Myocardial Infarction	(58)
Automated Myocardial Infarction Screening Using Morphology-Based Electrocardiogram Biomarkers	(59)
PSO Optimized Hybrid Deep Learning Model for Detection and Localization of Myocardial Infarction	(60)

An Evaluation of Machine Learning Classifiers for Detection of Myocardial Infarction Using Wavelet Entropy and Eigenspace Features	(61)
Accurate detection of myocardial infarction using non linear features with ECG signals	(62)
Deep Learning with a Recurrent Network Structure in the Sequence Modeling of Imbalanced Data for ECG-Rhythm Classifier	(63)
Deep Learning with Long Short-Term Memory for Enhancement Myocardial Infarction Classification	(64)
Use of Internet of Things to Provide a New Model for Remote Heart Attack Prediction	(65)
Identification of myocardial infarction using morphological features of electrocardiogram and vectorcardiogram	(66)
An Automated High-Accuracy Detection Scheme for Myocardial Ischemia Based on Multi-Lead Long-Interval ECG and Choi-Williams Time-Frequency Analysis Incorporating a Multi-Class SVM Classifier	(67)
Interpretability Analysis of Machine Learning Algorithms in the Detection of ST-Elevation Myocardial Infarction	(68)
Artificial intelligence to predict needs for urgent revascularization from 12-leads electrocardiography in emergency patients	(69)
Machine learning of microvolt-level 12-lead electrocardiogram can help distinguish takotsubo syndrome and acute anterior myocardial infarction	(70)
Neural Network Analysis of Electrocardiogram Signal	(71)
Preprocessing Method for Performance Enhancement in CNN-Based STEMI Detection From 12-Lead ECG	(72)
A novel method for detecting ST segment elevation myocardial infarction on a 12-lead electrocardiogram with a three-dimensional display	(73)
A Robustness Evaluation of Machine Learning Algorithms for ECG Myocardial Infarction Detection	(74)
A Novel Deep Learning Approach for Myocardial Infarction Detection and Multi-Label Classification	(75)
Advanced repeated structuring and learning procedure to detect acute myocardial ischemia in serial 12-lead ECGs	(76)
Automatic Triage of 12-Lead ECGs Using Deep Convolutional Neural Networks	(77)
Rationale and design of the artificial intelligence scalable solution for acute myocardial infarction (ASSIST) study	(78)
Detection of myocardial infarction based on novel deep transfer learning methods for urban healthcare in smart cities	(79)
IN THE DEVELOPMENT OF ARTIFICIAL INTELLIGENCE-GUIDED, SINGLE AND 12-LEAD ECG TO DETECT ST-ELEVATION MYOCARDIAL INFARCTION	(80)

Efficient detection of myocardial infarction from single lead ECG signal	(81)
EvoMBN: Evolving Multi-Branch Networks on Myocardial Infarction Diagnosis Using 12-Lead Electrocardiograms	(82)
SLC-GAN: An automated myocardial infarction detection model based on generative adversarial networks and convolutional neural networks with single-lead electrocardiogram synthesis	(83)
A robust myocardial infarction localization system based on multi-branch residual shrinkage network and active learning with clustering	(84)
A transferable in-silico augmented ischemic model for virtual myocardial perfusion imaging and myocardial infarction detection	(85)
INFARCTION AND OTHER ECG ABNORMALITIES USING PHYSIOLOGICALLY INTERPRETABLE FEATURES	(86)
Electrocardiogram Heartbeat Classification using Convolutional Neural Network-k Nearest Neighbor	(87)
Myocardial Infarction Detection and Classification-A New Multi-Scale Deep Feature Learning Approach	(88)
A Smartphone-Enabled Deep Learning Approach for Myocardial Infarction Detection Using ECG Traces for IoT-Based Healthcare Applications	(89)
SYSTEMS-LEVEL QUALITY IMPROVEMENT Detection of Cardiac Abnormalities from Multilead ECG using Multiscale Phase Alternation Features	(90)
The intestinal membrane permeability of dried bonito-derived dipeptides, which have an antihypertensive effect in vivo, suggests that dipeptides absorbed from the intestinal membrane may have an antihypertensive effect.	(91)
CLINICAL AND ADMINISTRATIVE BURDEN OF HIGH-INTENSITY REMOTE MONITORING	(92)
Tutorial: Acute Myocardial Ischemia The physiological underpinnings of acute myocardial ischemia	(93)
Artificial intelligence algorithm for detecting myocardial infarction using six-lead electrocardiography	(94)
LEAD ELECTROCARDIOGRAMS USING DEEP CONVOLUTIONAL NEURAL NETWORKS	(95)
MT-MV-KDF: A novel Multi-Task Multi-View Knowledge Distillation Framework for myocardial infarction detection and localization	(96)
Automated Diagnosis of Myocardial Infarction ECG Signals Using Sample Entropy in Flexible Analytic Wavelet Transform Framework	(97)
Morphology-Aware ECG Diagnostic Framework With Cross-Task Attention Transfer for Improved Myocardial Infarction Diagnosis	(98)

Myocardial infarction detection method based on the continuous T-wave area feature and multi-lead-fusion deep features	(99)
Diagnosis Myocardial Infarction Based on Stacking Ensemble of Convolutional Neural Network	(100)
Multi-branch myocardial infarction detection and localization framework based on multi-instance learning and domain knowledge	(101)
Multi-channel Lightweight Convolutional Neural Network for Remote Myocardial Infarction Monitoring	(102)
A Novel Approach for Detection of Myocardial Infarction From ECG Signals of Multiple Electrodes	(103)
A New Automatic Approach to Distinguish Myocardial Infarction Based on LSTM	(104)
Convolutional Dendrite Net detects myocardial infarction based on ECG signal measured by flexible sensor	(105)
Frequency-Enhanced Geometric-Constrained Reconstruction for Localizing Myocardial Infarction in 12-Lead Electrocardiograms	(106)
An interpretable shapelets-based method for myocardial infarction detection using dynamic learning and deep learning	(107)
A Residual-Dense-Based Convolutional Neural Network Architecture for Recognition of Cardiac Health Based on ECG Signals	(108)
Automated Detection of Myocardial Infarction and Heart Conduction Disorders Based on Feature Selection and a Deep Learning Model	(109)
Efficient Classification of ECG Images Using a Lightweight CNN with Attention Module and IoT	(110)
Acute Myocardial Infarction Detection Using Deep Learning on 12-Lead ECGs	(111)
Detection of Myocardial Infarction Using ECG and Multi-Scale Feature Concatenate	(112)
End-to-End Convolutional Neural Network Model to Detect and Localize Myocardial Infarction Using 12-Lead ECG Images without Preprocessing	(113)
Multilevel hybrid accurate handcrafted model for myocardial infarction classification using ECG signals	(114)
Improved Bat algorithm for the detection of myocardial infarction	(115)
A visually interpretable detection method combines 3-D ECG with a multi-VGG neural network for myocardial infarction identification	(116)
Classification of cardiac electrical signals between patients with myocardial infarction and normal subjects by using nonlinear dynamics features and different classification models	(117)

Continuous monitoring of acute myocardial infarction with a 3-Lead ECG system	(118)
Electrocardiogram Heartbeat Classification for Arrhythmias and Myocardial Infarction	(119)
Cross Subject Myocardial Infarction Detection From Vectorcardiogram Signals Using Binary Harry Hawks Feature Selection and Ensemble Classifiers	(120)
Design of an Integrated Myocardial Infarction Detection Model Using ECG Connectivity Features and Multivariate Time Series Classification	(121)
Impact of ECG data format on the performance of machine learning models for the prediction of myocardial infarction	(122)
Inferior Myocardial Infarction Detection From Lead II of ECG: A Gramian Angular Field-Based 2D-CNN Approach	(123)
A deep learning algorithm for detecting acute myocardial infarction	(124)
Dark Matter Constraints from a Unified Analysis of Strong Gravitational Lenses and Milky Way Satellite Galaxies	(125)
Machine Learning for Healthcare Multiple Instance Learning for ECG Risk Stratification	(126)
Automated detection of myocardial infarction using binary Harry Hawks feature selection and ensemble KNN classifier	(127)
Multiclass classification of myocardial infarction with convolutional and recurrent neural networks for portable ECG devices	(128)
ECG Codebook Model for Myocardial Infarction Detection	(129)
A machine learning approach for classifying healthy and infarcted patients using heart rate variabilities derived vector magnitude	(130)
Classification of myocardial infarction with multi-lead ECG signals and deep CNN	(130)
Evaluating Morphological Features of Electrocardiogram Signals for Diagnosing of Myocardial Infarction Using Classification-Based Feature Selection	(131)
Interpretable Assessment of ST-Segment Deviation in ECG Time Series	(132)
International evaluation of an artificial intelligence-powered electrocardiogram model detecting acute coronary occlusion myocardial infarction	(133)
Detection and Classification of Myocardial Infarction with Support Vector Machine Classifier Using Grasshopper Optimization Algorithm	(134)
Patient specific higher order tensor based approach for the detection and localization of myocardial infarction using 12-lead ECG	(135)
performance of a convolutional neural network derived from an ecG database in recognizing myocardial infarction	(136)
Detection of Myocardial Infarction from 12 Lead ECG Images	(137)

Automatic Classification of 15 Leads ECG Signal of Myocardial Infarction Using One Dimension Convolutional Neural Network	(138)
DIAGNOSIS OF ST-ELEVATED MYOCARDIAL INFARCTION BY ARTIFICIAL INTELLIGENCE WITH 12-LEAD ELECTROCARDIOGRAPH. THE ALERT-PILOT STUDY	(139)
Classification of myocardial infarction based on ECG signals and multi-network stacking model	(140)
Detection of Myocardial Infarction from ECG Signal Through Combining CNN and Bi-LSTM	(141)
An Effective Deep Learning Model for Automated Detection of Myocardial Infarction Based on Ultrashort-Term Heart Rate Variability Analysis	(142)
End-to-End Convolutional Neural Network Model to Detect and Localize Myocardial Infarction Using 12-Lead ECG Images without Preprocessing	(143)
An IoT-based Framework for Detecting Heart Conditions using Machine Learning	(144)
Application of higher-order spectra for the characterization of Coronary artery disease using electrocardiogram signals	(145)
Automatic diagnosis of the 12-lead ECG using a deep neural network	(146)
WaSP-ECG: A Wave Segmentation Pretraining Toolkit for Electrocardiogram Analysis	(147)
Detecting and interpreting myocardial infarction using fully convolutional neural networks	(148)
MI-OPTNET: AN OPTIMIZED DEEP LEARNING FRAMEWORK FOR MYOCARDIAL INFARCTION DETECTION	(149)
Automated detection of coronary artery disease, myocardial infarction and congestive heart failure using GaborCNN model with ECG signals	(150)
Heart rate dynamics in the prediction of coronary artery disease and myocardial infarction using artificial neural network and support vector machine	(151)
A multi-task channel attention network for Myocardial infarction detection and location using 12-lead ECGs	(152)
Detection and localization of myocardial infarction based on a convolutional autoencoder ☆	(153)
A novel automated diagnostic system for classification of myocardial infarction ECG signals using an optimal biorthogonal filter bank	(154)
A Retrospective Clinical Evaluation of an Artificial Intelligence Screening Method for Early Detection of STEMI in the Emergency Department	(155)
Diagnostic Accuracy of the Deep Learning Model for the Detection of ST Elevation Myocardial Infarction on Electrocardiogram	(156)

Electrocardiogram-based deep learning algorithm for the screening of obstructive coronary artery disease	(157)
Development of Clinically Validated Artificial Intelligence Model for Detecting ST-segment Elevation Myocardial Infarction	(158)
Automated Detection of Acute Myocardial Infarction Using Asynchronous Electrocardiogram Signals-Preview of Implementing Artificial Intelligence With Multichannel Electrocardiographs Obtained From Smartwatches: Retrospective Study	(159)
Development and validation of deep learning ECG-based prediction of myocardial infarction in emergency department patients	(160)
Explainable detection of myocardial infarction using deep learning models with Grad-CAM on ECG signals	(161)
Application of CNN for Detection and Localization of STEMI Using 12-Lead ECG Images	(162)
Identification of Coronary Culprit Lesion in ST Elevation Myocardial Infarction by Using Deep Learning	(163)
Artificial Intelligence-Powered Rapid Identification of ST-Elevation Myocardial Infarction via Electrocardiogram (ARISE) -A Pragmatic Randomized Controlled Trial	(164)
Detection of Myocardial Infarction Using ECG and Multi-Lead Fusion Deep Features	(165)
Machine Learning Methods in Predicting Patients with Suspected Myocardial Infarction Based on Short-Time HRV Data	(166)
Explainable Prediction of Acute Myocardial Infarction Using Machine Learning and Shapley Values	(167)
Electrocardiographic diagnosis of ST segment elevation myocardial infarction: An evaluation of three automated interpretation algorithms	(168)
Energy-efficient Real-time Myocardial Infarction Detection on Wearable Devices	(169)
Machine learning-based prediction of acute coronary syndrome using only the pre-hospital 12-lead electrocardiogram	(170)
Impact of Chronic Kidney Disease on Coronary Revascularization and In-Hospital Outcomes in Patients With Acute ST-Segment Elevation Myocardial Infarction	(171)
Myocardial Infarction Detection and Localization Using Optimal Features Based Lead Specific Approach	(172)
Interpretable Detection and Location of Myocardial Infarction Based on Ventricular Fusion Rule Features	(173)
Incorporation of Serial 12-Lead Electrocardiogram With Machine Learning to Augment the Out-of-Hospital Diagnosis of Non-ST Elevation Acute Coronary Syndrome	(174)

of Computers in Electrocardiology: 'What Should ECG Deep Learning Focus on? The diagnosis of acute coronary occlusion!'	(175)
Integrating multimodal information in machine learning for classifying acute myocardial infarction	(176)
Multi-scale SE-residual network with transformer encoder for myocardial infarction classification	(177)
Myocardial Infarction Detection using Morphological Features of ECG Signal	(178)
MI-CSBO: a hybrid system for myocardial infarction classification using deep learning and Bayesian optimization	(179)
Sleep MI: An AI-based screening algorithm for myocardial infarction using nocturnal electrocardiography	(180)
A Novel Real-Time Detection and Classification Method for ECG Signal Images Based on Deep Learning	(181)
Deep Neural Network Trained on Surface ECG Improves Diagnostic Accuracy of Prior Myocardial Infarction Over Q Wave Analysis	(182)
MYOCARDIAL INFARCTION DETECTION USING INTELLIGENT ALGORITHMS	(183)
A confident decision support system for interpreting electrocardiograms	(184)
Energy-Aware Design Methodology for Myocardial Infarction Detection on Low-Power Wearable Devices	(185)
Cardiac image analysis has advanced significantly in recent years.	(186)
Real-Time Multilead Convolutional Neural Network for Myocardial Infarction Detection	(187)
Automatic Classification of 15 Leads ECG Signal of Myocardial Infarction Using One-Dimension Convolutional Neural Network	(188)
MFB-CBRNN: A Hybrid Network for MI Detection Using 12-Lead ECGs	(189)
Identification and Localization of Myocardial Infarction Based on Analysis of ECG Signal in Cross Spectral Domain Using Boosted SVM Classifier	(190)
Automating detection and localization of myocardial infarction using shallow and end-to-end deep neural networks	(191)
Near real-time single-beat myocardial infarction detection from single-lead electrocardiogram using Long Short-Term Memory Neural Network	(192)
Localization and Classification of Myocardial Infarction Based on Artificial Neural Network	(193)
Inferior Myocardial Infarction Detection From Lead II of ECG: A Gramian Angular Field-Based 2D-CNN Approach	(194)
Automated Detection and Localization of Myocardial Infarction With staked Sparse Autoencoder and TreeBagger	(195)

A Lightweight Method of Myocardial Infarction Detection and Localization From Single Lead ECG Features Using Machine Learning Approach	(196)
Detection of Myocardial Infarction From 12-Lead ECG Trace Images Using Eigendomain Deep Representation Learning	(197)
Usefulness of multi-labelling artificialUsefulness of multi-labelling artificial intelligence in detecting rhythm disorders and acute ST-elevation myocardial infarction on 12-lead electrocardiogram intelligence in detecting rhythm disorders and acute ST-elevation myocardial infarction on 12-lead electrocardiogram	(198)
Artificial intelligence-assisted remote detection of ST-elevation myocardial infarction using a mini-12-lead electrocardiogram device in prehospital ambulance care	(199)
Temporal Feature-Based Classification Into Myocardial Infarction and Other CVDs Merging CNN and Bi-LSTM From ECG Signal	(200)
Machine Learning Classification of Coronary Heart Syndrome Based on Electrocardiogram Image Using Convolutional Neural Network Algorithm	(201)
International Evaluation of an Artificial Intelligence-Powered Ecg Model Detecting Occlusion Myocardial Infarction	(202)
Uncertainty quantification in DenseNet model using myocardial infarction ECG signals	(203)
Myocardial infarction detection method based on the continuous T-wave area feature and multi-lead-fusion deep features	(204)
Autonomous Detection of Myocardial Infarction and Other Ecg Abnormalities Using Physiologically Interpretable Features	(205)
An interpretable ensemble trees method with joint analysis of static and dynamic features for myocardial infarction detection	(206)
Single Lead for Stemi Detection Does Not Localize or Identify Culprit Lesion	(207)
TCT CONNECT-42 Artificial Intelligence Single Lead ECG as a Blueprint for Symptom-to-Balloon Time Reduction	(208)
TCT CONNECT-19 Base Camp to Everest Summit: Rapid Ascent of Artificial Intelligence Algorithms for ST-Elevation Myocardial Infarction Diagnosis	(209)
Automated Myocardial Infarction Detection in 12-Lead Electrocardiograms Using Deep Convolutional Neural Networks	(210)
Application of Heartbeat-Attention Mechanism for Detection of Myocardial Infarction Using 12-Lead ECG Records	(211)
Third-order tensor based analysis of multilead ECG for classification of myocardial infarction	(212)

Artificial Intelligence Detection of Occlusive Myocardial Infarction from Electrocardiograms Interpreted as “Normal” by Conventional Algorithms	(213)
Hybrid Network with Attention Mechanism for Detection and Location of Myocardial Infarction Based on 12-Lead Electrocardiogram Signals	(214)
Repeated Structuring & Learning Procedure for Detection of Myocardial Ischemia: a Robustness Analysis	(215)
ROMIAE (Rule-Out Acute Myocardial Infarction Using Artificial Intelligence Electrocardiogram Analysis) trial study protocol: a prospective multicenter observational study for validation of a deep learning-based 12-lead electrocardiogram analysis model for	(216)
DeepMI: Deep multi-lead ECG fusion for identifying myocardial infarction and its occurrence-time	(217)
Using Multi-Task Learning-Based Framework to Detect ST-Segment and J-Point Deviation From Holter	(218)
A Multi-Domain Feature Fusion CNN for Myocardial Infarction Detection and Localization	(219)
The Role of the ECG in the Primary PCI Pathway: A Clinical Perspective	(220)

Bibiography

.1 Gibson CM, Mehta S, Ceschim MR, Frauenfelder A, Vieira D, Botelho R, et al. Evolution of single-lead ECG for STEMI detection using a deep learning approach. International journal of cardiology. 2022;346:47-52.

.2 Chen Y, Chen H, He Z, Yang C, Cao Y, editors. Multi-channel lightweight convolution neural network for anterior myocardial infarction detection. 2018 IEEE SmartWorld, Ubiquitous Intelligence & Computing, Advanced & Trusted Computing, Scalable Computing & Communications, Cloud & Big Data Computing, Internet of People and Smart City Innovation (SmartWorld/SCALCOM/UIC/ATC/CBDCom/IOP/SCI); 2018: IEEE.

.3 Hao P, Gao X, Li Z, Zhang J, Wu F, Bai C. Multi-branch fusion network for Myocardial infarction screening from 12-lead ECG images. Computer methods and programs in biomedicine. 2020;184:105286.

.4 Wang H, Zhao W, Jia D, Hu J, Li Z, Yan C, et al., editors. Myocardial infarction detection based on multi-lead ensemble neural network. 2019 41st Annual international conference of the IEEE engineering in medicine and biology society (EMBC); 2019: IEEE.

.5 Zhang X, Gu K, Miao S, Zhang X, Yin Y, Wan C, et al. Automated detection of cardiovascular disease by electrocardiogram signal analysis: a deep learning system. Cardiovascular Diagnosis and Therapy. 20.227:(2)10;20

.6 Zhao Y, Xiong J, Hou Y, Zhu M, Lu Y, Xu Y, et al. Early detection of ST-segment elevated myocardial infarction by artificial intelligence with 12-lead electrocardiogram. International Journal of Cardiology. 2020;317:223-30.

.7 Fu L, Lu B, Nie B, Peng Z, Liu H, Pi X. Hybrid network with attention mechanism for detection and location of myocardial infarction based on 12-lead electrocardiogram signals. Sensors. 2020;20(4):1020.

.8 Sun Q, Xu Z, Liang C, Zhang F, Li J, Liu R, et al. A dynamic learning-based ECG feature extraction method for myocardial infarction detection. Physiological measurement. 2023;43(12):124005.
.9 Que W, Han C, Zhao X, Shi L. An ECG generative model of myocardial infarction. Computer Methods and Programs in Biomedicine.225:107062;2022 .
.10 Han C, Pan S, Que W, Wang Z, Zhai Y, Shi L. Automated localization and severity period prediction of myocardial infarction with clinical interpretability based on deep learning and knowledge graph. Expert Systems with Applications. .209:118398;2022
.11 Zhang J, Liu M, Xiong P, Du H, Zhang H, Sun G, et al. Automated localization of myocardial infarction of image-based multilead ECG tensor with Tucker2 decomposition. IEEE Transactions on Instrumentation and Measurement. 2021;71:1-15.
.12 Wu L, Huang G, Yu X, Ye M, Liu L, Ling Y, et al. Deep learning networks accurately detect ST-segment elevation myocardial infarction and culprit vessel. Frontiers in cardiovascular medicine. 2022;9:797207.
.13 Xu J, Zhang Y, Yu H, Lin B, Xiang P, Lin T, et al. High performance of privacy-preserving acute myocardial infarction auxiliary diagnosis based on federated learning: a multicenter retrospective study. Annals of Translational Medicine. 2022;10(18):1006.
.14 Gregg RE, An J, Babaeizadeh S, Faramand Z, Bouzid Z, Al Zaiti S. ECG detection of ACS by convolutional neural network utilizing transfer learning. Journal of Electrocardiology. 2022;73:18-9.
.15 Wu L, Zhou B, Liu D, Wang L, Zhang X, Xu L, et al. LASSO regression-based diagnosis of acute ST-segment elevation myocardial infarction (STEMI) on electrocardiogram (ECG). Journal of Clinical Medicine. 2022;11(18):5408.
.16 Tang P, Wang Q, Ouyang H, Yang S, Hua P. The feasibility of early detecting coronary artery disease using deep learning-based algorithm based on electrocardiography. Aging (Albany NY). 2023;15(9):3524.
.17 Chen KW, Wang YC, Liu MH, Tsai BY, Wu MY, Hsieh PH, et al. Artificial intelligence-assisted remote detection of ST-elevation myocardial infarction using a mini-12-lead electrocardiogram device in prehospital ambulance care. Front Cardiovasc Med. 2022;9:1001982.
.18 Wang Y-C, Chen K-W, Tsai B-Y, Wu M-Y, Hsieh P-H, Wei J-T, et al., editors. Implementation of an all-day artificial intelligence–based triage system to accelerate door-to-balloon times. Mayo Clinic Proceedings; 2022: Elsevier.
.19 Tadesse GA, Javed H, Weldemariam K, Liu Y, Liu J, Chen J, et al. DeepMI: Deep multi-lead ECG fusion for identifying myocardial infarction and its occurrence-time. Artif Intell Med. 2021;121:10.2192
.20 Yao L, Liu C, Li P, Wang J, Liu Y, Li W, et al. Enhanced automated diagnosis of coronary artery disease using features extracted from QT interval time series and ST–T waveform. IEEE Access. 2020;8:129510-24.
.21 Liu N, Wang L, Chang Q, Xing Y, Zhou X. A simple and effective method for detecting myocardial infarction based on deep convolutional neural network. Journal of Medical Imaging and Health Informatics. 2018;8(7):1508-12.
.22 Zhang G, Si Y, Wang D, Yang W, Sun Y. Automated detection of myocardial infarction using a gramian angular field and principal component analysis network. IEEe Access. 2019;7:171570-83.
.23 Han C, Shi L. Automated interpretable detection of myocardial infarction fusing energy entropy and morphological features. Computer methods and programs in biomedicine. 2019;175:9-23.

.24 Feng K, Pi X, Liu H, Sun K. Myocardial infarction classification based on convolutional neural network and recurrent neural network. Applied Sciences. 2019;9(9):1879.
.25 Wang Z, Qian L, Han C, Shi L. Application of multi-feature fusion and random forests to the automated detection of myocardial infarction. Cognitive Systems Research. 2020;59:15-26.
.26 Zeng W, Yuan J, Yuan C, Wang Q, Liu F, Wang Y. Classification of myocardial infarction based on hybrid feature extraction and artificial intelligence tools by adopting tunable-Q wavelet transform (TQWT), variational mode decomposition (VMD) and neural networks. Artificial Intelligence in Medicine. 2020;106:101848.
.27 Han C, Shi L. ML–ResNet: A novel network to detect and locate myocardial infarction using 12 leads ECG. Computer methods and programs in biomedicine. 2020;185:105138.
.28 Zhang J, Liu M, Xiong P, Du H, Zhang H, Lin F, et al. A multi-dimensional association information analysis approach to automated detection and localization of myocardial infarction. Engineering Applications of Artificial Intelligence. 2021;97:104092.
.29 Xiong P, Xue Y, Zhang J, Liu M, Du H, Zhang H, et al. Localization of myocardial infarction with multi-lead ECG based on DenseNet. Computer Methods and Programs in Biomedicine. 2021;203:106024.
.30 He Z, Yuan Z, An P, Zhao J, Du B. MFB-LANN: A lightweight and updatable myocardial infarction diagnosis system based on convolutional neural networks and active learning. Computer Methods and Programs in Biomedicine. 2021;210:106379.
.31 Cao Y, Wei T, Zhang B, Lin N, Rodrigues JJ, Li J, et al. ML-Net: Multi-channel lightweight network for detecting myocardial infarction. IEEE Journal of Biomedical and Health Informatics. 2.31-3721:(10)25;021
.32 Ge D, Zhou W. Discrimination of different myocardial infarction stages using wide band electrocardiogram. Biomedical Signal Processing and Control. 2016;25:143-9.
.33 Liu W, Huang Q, Chang S, Wang H, He J. Multiple-feature-branch convolutional neural network for myocardial infarction diagnosis using electrocardiogram. Biomedical Signal Processing and Control. 2018;45:22-32.
.34 Chen X, Guo W, Zhao L, Huang W, Wang L, Sun A, et al. Acute myocardial infarction detection using deep learning-enabled electrocardiograms. Frontiers in cardiovascular medicine. 2021;8:654515.
.35 Zeng W, Shan L, Yuan C, Du S. Detection of myocardial infarction using Shannon energy envelope, FA-MVEMD and deterministic learning. Complex & Intelligent Systems..73-4755:(4)10;2024
.36 Martin H, Morar U, Izquierdo W, Cabrerizo M, Cabrera A, Adjouadi M. Real-time frequency-independent single-Lead and single-beat myocardial infarction detection. Artificial intelligence in medicine. 2021;121:102179.
.37 Xiong P, Lee SM-Y, Chan G. Deep learning for detecting and locating myocardial infarction by electrocardiogram: A literature review. Frontiers in cardiovascular medicine. 2022;9:860032.
.38 Xu W, Wang L, Wang B, Cheng W. Intelligent Recognition Algorithm of Multiple Myocardial Infarction Based on Morphological Feature Extraction. Processes. 2022;10(11):2348.
.39 He C, Liu M, Xiong P, Yang J, Du H, Xu J, et al. Localization of myocardial infarction using a multi-branch weight sharing network based on 2-D

vectorcardiogram. Engineering Applications of Artificial Intelligence. 2022;116:105428.
.40 Zeng W, Yuan C. Myocardial infarction detection using ITD, DWT and deterministic learning based on ECG signals. Cognitive Neurodynamics. 2023;17(4):941-64.
.41 Xiong P, Yang L ,Zhang J, Xu J, Yang J, Wang H, et al. Detection of inferior myocardial infarction based on multi branch hybrid network. Biomedical Signal Processing and Control. 2023;84:104725.
.42 Liu K, Liu T, Wen D, Zang M, Zhou S, Liu C. SRTNet: Scanning, Reading, and Thinking Network for myocardial infarction detection and localization. Expert Systems with Applications. 2024;240:122402.
.43 Parmar R, Yadav K, Anand G, Trivedi G. An SNN-Inspired Area- and Power-Efficient VLSI Architecture of Myocardial Infarction Classifier for Wearable Devices. IEEE Transactions on Circuits and Systems II: Express Briefs. 2024;71(6):3191-5.
.44 Wang J, Guo X. Automated detection of myocardial infarction based on an improved state refinement module for LSTM/GRU. Artificial Intelligence in Medicine. 2024;152:102865.
.45 Qiang Y, Dong X, Yang Y. Automatic detection and localisation of myocardial infarction using multi-channel dense attention neural network. Biomedical Signal Processing and Control. 2024;89:105766.
.46 Zhang X, Li R, Dai H, Liu Y, Zhou B, Wang Z. Localization of Myocardial Infarction With Multi-Lead Bidirectional Gated Recurrent Unit Neural Network. IEEE Access. 2019;7:161152-66.
.47 Jikui L, Ruxin W, Bo W, Zengding L, Fen M, Ye L. Myocardial Infarction Detection and Localization with Electrocardiogram Based on Convolutional Neural Network. 2021;30(5):833-42.
.48 He Z, Yuan S, Zhao J, Du B, Yuan Z, Alhudhaif A, et al. A novel myocardial infarction localization method using multi-branch DenseNet and spatial matching-based active semi-supervised learning. Information Sciences. 2022;606:649-68.
.49 Acharya UR, Fujita H, Oh SL, Hagiwara Y, Tan JH, Adam M. Application of deep convolutional neural network for automated detection of myocardial infarction using ECG signals. Information Sciences. 2017;415-416:190-8.
.50 Zhang J, Liu M, Xiong P, Du H, Yang J, Xu J, et al. Automated Localization of Myocardial Infarction From Vectorcardiographic via Tensor Decomposition. IEEE Transactions on Biomedical Engineering. 2023;70(3):812.23-
.51 Deng Y, Gao Z, Xu S, Ren P, Wen Y, Mao Y, et al. ST-Net: Synthetic ECG tracings for diagnosing various cardiovascular diseases. Biomedical Signal Processing and Control. 2020;61:101997.
.52 Jahmunah V, Ng EYK, Tan R-S, Oh SL, Acharya UR. Explainable detection of myocardial infarction using deep learning models with Grad-CAM technique on ECG signals. Computers in Biology and Medicine. 2022;146:105550.
.53 Kapfo A, Dandapat S, Kumar Bora P. Automated detection of myocardial infarction from ECG signal using variational mode decomposition based analysis. 2020;7(6):155-60.
.54 Yadav SS, More SB, Jadhav SM, Sutar SR, editors. Convolutional Neural Networks Based Diagnosis of Myocardial Infarction in Electrocardiograms. 2021 International Conference on Computing, Communication, and Intelligent Systems (ICCCIS); 2021 19-20 Feb. 2021.
.55 Kora P. ECG based Myocardial Infarction detection using Hybrid Firefly Algorithm. Computer Methods and Programs in Biomedicine. 2017;152:141-8.

.56 Sharma LD, Sunkaria RK .Inferior myocardial infarction detection using stationary wavelet transform and machine learning approach. Signal, Image and Video Processing. 2018;12(2):199-206.
.57 Prabhakararao E, Dandapat S. Myocardial Infarction Severity Stages Classification From ECG Signals Using Attentional Recurrent Neural Network. IEEE Sensors Journal. 2020;20(15):8711-20.
.58 Kumar R, Aggarwal Y, Nigam VK. Autonomic Features in Prediction of Coronary Artery Disease and Myocardial Infarction. IETE Journal of Research. 2023;69.61-8354:(11)
.59 Jahnavi D, Dash A, Ghosh N, Patra A, editors. Automated Myocardial Infarction Screening Using Morphology-Based Electrocardiogram Biomarkers. 2023 45th Annual International Conference of the IEEE Engineering in Medicine & Biology Society (EMBC); 2023 24-27 July 2023.
.60 Sahu G, Ray KC. PSO Optimized Hybrid Deep Learning Model for Detection and Localization of Myocardial Infarction. IEEE Sensors Journal. 2024;24(5):6643-54.
.61 Choudhary PS, Dandapat S, editors. An Evaluation of Machine Learning Classifiers for Detection of Myocardial Infarction Using Wavelet Entropy and Eigenspace Features. 2020 IEEE Applied Signal Processing Conference (ASPCON); 2020 7-9 Oct. 2020.
.62 Sridhar C, Lih OS, Jahmunah V, Koh JEW, Ciaccio EJ, San TR, et al. Accurate detection of myocardial infarction using non linear features with ECG signals. Journal of Ambient Intelligence and Humanized Computing. 2021;12(3):3227-44.
.63 Darmawahyuni A, Nurmaini S, Sukemi, Caesarendra W, Bhayyu V, Rachmatullah MN, et al. Deep Learning with a Recurrent Network Structure in the Sequence Modeling of Imbalanced Data for ECG-Rhythm Classifier. Algorithms. 2019;12(6):118.
.64 Darmawahyuni A, Nurmaini S, Sukemi, editors. Deep Learning with Long Short-Term Memory for Enhancement Myocardial Infarction Classification. 2019 6th International Conference on Instrumentation, Control, and Automation (ICA); 2019 31 July-2 Aug. 2019.
.65 Yahyaie M, Tarokh MJ, Mahmoodyar MA. Use of Internet of Things to Provide a New Model for Remote Heart Attack Prediction. Telemedicine and e-Health. 2019;25(6):499-510.
.66 Hafshejani NJ, Mehridehnavi A, Hajian R, Boudagh S, Behjati M. Identification of myocardial infarction using morphological features of electrocardiogram and vectorcardiogram. 2021;15(9):.85-674
.67 Hussein AF, Hashim SJ, Rokhani FZ, Wan Adnan WA. An Automated High-Accuracy Detection Scheme for Myocardial Ischemia Based on Multi-Lead Long-Interval ECG and Choi-Williams Time-Frequency Analysis Incorporating a Multi-Class SVM Classifier. Sensors. 2021;21(7):2311.
.68 Bodini M, Rivolta MW, Sassi R, editors. Interpretability Analysis of Machine Learning Algorithms in the Detection of ST-Elevation Myocardial Infarction. 2020 Computing in Cardiology; 2020 13-16 Sept. 2020.
.69 Goto S, Kimura M, Katsumata Y, Goto S, Kamatani T, Ichihara G, et al. Artificial intelligence to predict needs for urgent revascularization from 12-leads electrocardiography in emergency patients. PLOS ONE. 2019;14(1):e0210103.
.70 Shimizu M, Suzuki M, Fujii H, Kimura S, Nishizaki M, Sasano T. Machine learning of microvolt-level 12-lead electrocardiogram can help distinguish takotsubo syndrome and acute anterior myocardial infarction. Cardiovascular Digital Health Journal. 2022;3(4):179-88.

.71 Alimbayeva ZN, Ozhikenov KA ,Ozhikenova AK, Bodin ON, Gerasimov AI, Mukazhanov YB, editors. Neural Network Analysis of Electrocardiogram Signal. 2019 20th International Conference of Young Specialists on Micro/Nanotechnologies and Electron Devices (EDM); 2019 29 June-3 July 2019.
.72 Park Y, Yun ID, Kang SH. Preprocessing Method for Performance Enhancement in CNN-Based STEMI Detection From 12-Lead ECG. IEEE Access. 2019;7:99964-77.
.73 Heo J, Lee JJ, Kwon S, Kim B, Hwang SO, Yoon YR. A novel method for detecting ST segment elevation myocardial infarction on a 12-lead electrocardiogram with a three-dimensional display. Biomedical Signal Processing and Control. 2020;56:101700.
.74 Sraitih M, Jabrane Y, Hajjam El Hassani A. A Robustness Evaluation of Machine Learning Algorithms for ECG Myocardial Infarction Detection. Journal of Clinical Medicine. 2022;11(17):4935.
.75 Abbas S, Ojo S, Krichen M, Alamro MA, Mihoub A, Vilcekova L. A Novel Deep Learning Approach for Myocardial Infarction Detection and Multi-Label Classification. IEEE Access. 2024;12:76003-21.
.76 Sbrollini A, ter Haar CC, Leoni C, Morettini M, Burattini L, Swenne CA. Advanced repeated structuring and learning procedure to detect acute myocardial ischemia in serial 12-lead ECGs. Physiological Measurement. 2023;44(8):08400.3
.77 van de Leur RR, Blom LJ, Gavves E, Hof IE, van der Heijden JF, Clappers NC, et al. Automatic Triage of 12-Lead ECGs Using Deep Convolutional Neural Networks. Journal of the American Heart Association. 2020;9(10):e015138.
.78 Domingo-Gardeta T, Montero-Cabezas JM, Jurado-Román A, Sabaté M, Aboal J, Baranchuk A, et al. Rationale and design of the artificial intelligence scalable solution for acute myocardial infarction (ASSIST) study. Journal of Electrocardiology. 2024;86:153768.
.79 Alghamdi A, Hammad M, Ugail H, Abdel-Raheem A, Muhammad K, Khalifa HS, et al. Detection of myocardial infarction based on novel deep transfer learning methods for urban healthcare in smart cities. Multimedia Tools and Applications. 2024;83(5):14913-34.
.80 Mehta S, Vieira D, Guillen V, Zerpa D, Quintana A, Sanchez C, et al. Artificial intelligence-guided, single-lead EKG may be a game-changer for symptom-to-balloon time reduction in ST-elevated myocardial infarction. European Heart Journal. 2022;43(Supplement_2):ehac544..1170
.81 Fatimah B, Singh P, Singhal A, Pramanick D, S P, Pachori RB. Efficient detection of myocardial infarction from single lead ECG signal. Biomedical Signal Processing and Control. 2021;68:102678.
.82 Liu W, Ji J, Chang S, Wang H, He J, Huang Q. EvoMBN: Evolving Multi-Branch Networks on Myocardial Infarction Diagnosis Using 12-Lead Electrocardiograms. Biosensors. 2022;12(1):15.
.83 Li W, Tang YM, Yu KM, To S. SLC-GAN: An automated myocardial infarction detection model based on generative adversarial networks and convolutional neural networks with single-lead electrocardiogram synthesis. Information Sciences. 2022;589:738-50.
.84 He Z, Yuan S, Zhao J, Yuan Z, Chen Y. A robust myocardial infarction localization system based on multi-branch residual shrinkage network and active learning with clustering. Biomedical Signal Processing and Control. 2023;80:104238.
.85 Harnod Z, Lin C, Yang H-W, Wang Z-W, Huang H-L, Lin T-Y, et al. A transferable in-silico augmented ischemic model for virtual myocardial perfusion

imaging and myocardial infarction detection. Medical Image Analysis. 2024;93:103087.
.86 Gliner V, Levy I, Tsutsui K, Acha MR, Schliamser J, Schuster A, et al. Clinically meaningful interpretability of an AI model for ECG classification. npj Digital Medicine. 2025;8(1):109.
.87 Abdul ZK, Al-Talabani AK, Rahman CM, Asaad SM. Electrocardiogram Heartbeat Classification using Convolutional Neural Network-k Nearest Neighbor. ARO-THE SCIENTIFIC JOURNAL OF KOYA UNIVERSITY. 2024;12(1):61-7.
.88 Wu JF, Bao YL, Chan SC, Wu HC, Zhang L, Wei XG, editors. Myocardial infarction detection and classification — A new multi-scale deep feature learning approach. 2016 IEEE International Conference on Digital Signal Processing (DSP); 2016 16-18 Oct. 2016.
.89 Parupudi VS, Panda AK, Tripathy RK. A Smartphone-Enabled Deep Learning Approach for Myocardial Infarction Detection Using ECG Traces for IoT-Based Healthcare Applications. IEEE Sensors Letters. 2023;7(11):1-4.
.90 Tripathy RK, Dandapat S. Detection of Cardiac Abnormalities from Multilead ECG using Multiscale Phase Alternation Features. Journal of Medical Systems. 2016;40(6):143.
.91 Miralles B, Amigo L, Recio I. Critical Review and Perspectives on Food-Derived Antihypertensive Peptides. Journal of Agricultural and Food Chemistry. 2018;66(36):9384-90.
.92 Haddad TC, Coffey JD, Deng Y, Glasgow AE, Christopherson LA, Sangaralingham LR, et al. Impact of a High-Risk, Ambulatory COVID-19 Remote Patient Monitoring Program on Utilization, Cost, and Mortality. Mayo Clinic Proceedings. 2022;97(12):2215-25.
.93 Macleod R, Zenger B, Bergquist JA, Rupp LC, Good WWJJoE. Tutorial: Acute Myocardial Ischemia The physiological underpinnings of acute myocardial ischemia. 2022.
.94 Cho Y, Kwon J-m, Kim K-H, Medina-Inojosa JR, Jeon K-H, Cho S, et al. Artificial intelligence algorithm for detecting myocardial infarction using six-lead electrocardiography. Scientific Reports. 2020;10(1):20495.
.95 van de Leur RR, van Sleuwen M, Zwetsloot PM, van der Harst P, Doevendans PA, Hassink RJ, et al. Automatic triage of twelve-lead electrocardiograms using deep convolutional neural networks: a first implementation study. European heart journal Digital health. 2024;5(1):89-96.
.96 Qiang Y, Dong X, Liu X, Yang Y. MT-MV-KDF: A novel Multi-Task Multi-View Knowledge Distillation Framework for myocardial infarction detection and localization. Biomedical Signal Processing and Control. 2024;95:106382.
.97 Kumar M, Pachori RB, Acharya UR. Automated Diagnosis of Myocardial Infarction ECG Signals Using Sample Entropy in Flexible Analytic Wavelet Transform Framework. Entropy. 2017;19(9):488.
.98 Choudhary PS, Dandapat S. Morphology-Aware ECG Diagnostic Framework With Cross-Task Attention Transfer for Improved Myocardial Infarction Diagnosis. IEEE Transactions on Instrumentation and Measurement. 2024;73:1-11.
.99 Jiang M, Bian F, Zhang J, Huang T, Xia L, Chu Y, et al. Myocardial infarction detection method based on the continuous T-wave area feature and multi-lead-fusion deep features. 2024;45.
.100 Elmannai H, Saleh H, Algarni AD, Mashal I, Kwak KS, El-Sappagh S, et al. Diagnosis Myocardial Infarction Based on Stacking Ensemble of Convolutional Neural Network. Electronics. 2022;11(23):3976.

.101 Li X, Huang Y, Ning Y, Wang M, Cai W. Multi-branch myocardial infarction detection and localization framework based on multi-instance learning and domain knowledge. Physiological Measurement. 2024;45(4):045009.
.102 Cao Y, Wei T, Lin N, Zhang D, Rodrigues JJPC, editors. Multi-Channel Lightweight Convolutional Neural Network for Remote Myocardial Infarction Monitoring. 2020 IEEE Wireless Communications and Networking Conference Workshops (WCNCW); 2020 6-9 April 2020.
.103 Tripathy RK, Bhattacharyya A, Pachori RB. A Novel Approach for Detection of Myocardial Infarction From ECG Signals of Multiple Electrodes. IEEE Sensors Journal. 2019;19(12):4509-17.
.104 Zhang X, Li R, Hu Q, Zhou B, Wang Z, editors. A New Automatic Approach to Distinguish Myocardial Infarction Based on LSTM. 2019 8th International Symposium on Next Generation Electronics (ISNE); 2019 9-10 Oct. 2019.
.105 Majumdar D, Tüzün B, Pal TK, Saini RV, Bankura K, Mishra D. Structurally diverse heterobimetallic Pb (II)-Salen complexes mechanistic notion of cytotoxic activity against neuroblastoma cancer cell: Synthesis, characterization, protein–ligand interaction profiler, and intuitions from DFT. Polyhedron. 2021;210:115504.
.106 Lian S, Gao Z, Wang H, Liu X, Xu L, Liu H, et al. Frequency-Enhanced Geometric-Constrained Reconstruction for Localizing Myocardial Infarction in 12-Lead Electrocardiograms. IEEE Transactions on Biomedical Engineering. 2024;71(9):2599-611.
.107 Qu J, Sun Q, Wu W, Zhang F, Liang C, Chen Y, et al. An interpretable shapelets-based method for myocardial infarction detection using dynamic learning and deep learning. Physiological Measurement. 2024;45(3):035001.
.108 Ahmed AES, Abbas Q, Daadaa Y, Qureshi I, Perumal G, Ibrahim MEA. A Residual-Dense-Based Convolutional Neural Network Architecture for Recognition of Cardiac Health Based on ECG Signals. Sensors. 2023;23(16):7204.
.109 Hammad M, Chelloug SA, Alkanhel R, Prakash AJ, Muthanna A, Elgendy IA, et al. Automated Detection of Myocardial Infarction and Heart Conduction Disorders Based on Feature Selection and a Deep Learning Model. Sensors. 2022;22(17):6503.
.110 Sadad T, Safran M, Khan I, Alfarhood S, Khan R, Ashraf I. Efficient Classification of ECG Images Using a Lightweight CNN with Attention Module and IoT. Sensors. 2023;23(18):7697.
.111 Chen X, Guo W, Zhao L, Huang W, Wang L, Sun A, et al. Acute Myocardial Infarction Detection Using Deep Learning-Enabled Electrocardiograms. 2021;8.
.112 Jian J-Z, Ger T-R, Lai H-H, Ku C-M, Chen C-A, Abu PAR, et al. Detection of Myocardial Infarction Using ECG and Multi-Scale Feature Concatenate. Sensors. 2021;21(5):1906.
.113 Uchiyama R, Okada Y, Kakizaki R, Tomioka S. End-to-End Convolutional Neural Network Model to Detect and Localize Myocardial Infarction Using 12-Lead ECG Images without Preprocessing. Bioengineering (Basel, Switzerland). 2022.(9)9;
.114 Barua PD, Aydemir E, Dogan S, Kobat MA, Demir FB, Baygin M, et al. Multilevel hybrid accurate handcrafted model for myocardial infarction classification using ECG signals. International Journal of Machine Learning and Cybernetics. 2023;14(5):1.68-651
.115 Kora P, Kalva SR. Improved Bat algorithm for the detection of myocardial infarction. SpringerPlus. 2015;4(1):666.
.116 Fang R, Lu C-C, Chuang C-T, Chang W-H. A visually interpretable detection method combines 3-D ECG with a multi-VGG neural network for myocardial

infarction identification. Computer Methods and Programs in Biomedicine. 2022;219:106762.
.117 Deng M, Huang X, Liang Z, Lin W, Mo B, Liang D, et al. Classification of cardiac electrical signals between patients with myocardial infarction and normal subjects by using nonlinear dynamics features and different classification models. Biomedical Signal Processing and Control. 2023;79:104105.
.118 Aranda Hernandez A, Bonizzi P, Peeters R, Karel J. Continuous monitoring of acute myocardial infarction with a 3-Lead ECG system. Biomedical Signal Processing and Control. 2023;79:104041.
.119 Pham B-T, Le PT, Tai T-C, Hsu Y-C, Li Y-H, Wang J-C. Electrocardiogram Heartbeat Classification for Arrhythmias and Myocardial Infarction. Sensors. 2023;2.2993:(6)3
.120 Chaitanya MK, Sharma LD. Cross Subject Myocardial Infarction Detection From Vectorcardiogram Signals Using Binary Harry Hawks Feature Selection and Ensemble Classifiers. IEEE Access. 2024;12:28247-59.
.121 Jain P, Deshmukh A, Padole H. Design of an Integrated Myocardial Infarction Detection Model Using ECG Connectivity Features and Multivariate Time Series Classification. IEEE Access. 2024;12:9070-81.
.122 Bellfield RAA, Ortega-Martorell S, Lip GYH, Oxborough D, Olier I. Impact of ECG data format on the performance of machine learning models for the prediction of myocardial infarction. Journal of Electrocardiology. 2024;84:17-26.
.123 Yousuf A, Hafiz R, Riaz S, Farooq MA, Riaz K, Rahman MMUJISL. Inferior Myocardial Infarction Detection From Lead II of ECG: A Gramian Angular Field-Based 2D-CNN Approach. 2023;8:1-4.
.124 Liu WC, Lin CS, Tsai CS, Tsao TP, Cheng CC, Liou JT, et al. A deep learning algorithm for detecting acute myocardial infarction. EuroIntervention : journal of EuroPCR in collaboration with the Working Group on Interventional Cardiology of the European Society of Cardiology. 2021;17(9):765-73.
.125 Nadler EO, Birrer S, Gilman D, Wechsler RH, Du X, Benson A, et al. Dark Matter Constraints from a Unified Analysis of Strong Gravitational Lenses and Milky Way Satellite Galaxies. The Astrophysical Journal. 2021;917(1):7.
.126 Shanmugam D, Blalock D, Guttag J. Multiple Instance Learning for ECG Risk Stratification. In: Finale D-V, Jim F, Ken J, David K, Rajesh R, Byron W, et al., editors. Proceedings of the 4th Machine Learning for Healthcare Conference; Proceedings of Machine Learning Research: PMLR; 2019. p. 124--39.
.127 Chaitanya MK, Sharma LD. Automated detection of myocardial infarction using binary Harry Hawks feature selection and ensemble KNN classifier. Computer Methods in Biomechanics and Biomedical Engineering. 2024;27(14):2024-40.
.128 Lui HW, Chow KL. Multiclass classification of myocardial infarction with convolutional and recurrent neural networks for portable ECG devices. Informatics in Medicine Unlocked. 2018;13:26-33.
.129 Cao D, Lin D, Lv Y, editors. ECG codebook model for Myocardial Infarction detection. 2014 10th International Conference on Natural Computation (ICNC); 2014 19-21 Aug. 2014.
.130 Agrawal RK, Sewani RR, Delen D, Benjamin B. A machine learning approach for classifying healthy and infarcted patients using heart rate variabilities derived vector magnitude. Healthcare Analytics. 2022;2:100121.
.131 Mahmoudinejad SA, Safdarian N. Evaluating Morphological Features of Electrocardiogram Signals for Diagnosing of Myocardial Infarction Using

Classification-Based Feature Selection. Journal of Medical Signals & Sensors. 2021;11(2):79-91.
.132 Campero Jurado I, Fedjajevs A, Vanschoren J, Brombacher A. Interpretable Assessment of ST-Segment Deviation in ECG Time Series. Sensors. 2022;22(13):4919.
.133 Herman R, Meyers HP, Smith SW, Bertolone DT, Leone A, Bermpeis K, et al. International evaluation of an artificial intelligence–powered electrocardiogram model detecting acute coronary occlusion myocardial infarction. European Heart Journal - Digital Health. 2024;5(2):123-33.
.134 Safdarian N, Nezhad SYD, Dabanloo NJ. Detection and Classification of Myocardial Infarction with Support Vector Machine Classifier Using Grasshopper Optimization Algorithm. Journal of Medical Signals & Sensors. 2021;11(3):185-93.
.135 Chauhan C, Tripathy RK, Agrawal M. Patient specific higher order tensor based approach for the detection and localization of myocardial infarction using 1-2lead ECG. Biomedical Signal Processing and Control. 2023;83:104701.
.136 Makimoto H, Höckmann M, Lin T, Glöckner D, Gerguri S, Clasen L, et al. Performance of a convolutional neural network derived from an ECG database in recognizing myocardial infarction. Scientific Reports. 2020;10(1):8445.
.137 Sane RKS, Choudhary PS, Sharma LN, Dandapat PS, editors. Detection of Myocardial Infarction from 12 Lead ECG Images. 2021 National Conference on Communications (NCC); 2021 27-30 July 2021.
.138 Mirza AH, Nurmaini S, Partan RU. Automatic Classification of 15 Leads ECG Signal of Myocardial Infarction Using One Dimension Convolutional Neural Network. Applied Sciences. 2022;12(11):5603.
.139 Zhang Y, Zhao Y, Xu Y. DIAGNOSIS OF ST-ELEVATED MYOCARDIAL INFARCTION BY ARTIFICIAL INTELLIGENCE WITH 12-LEAD ELECTROCARDIOGRAPH. THE ALERT-PILOT STUDY. Journal of Hypertension. 2019;37:e234.
.140 Zhao T, Deng M, Lin P, Wang J, Cao J, editors. Classification of myocardial infarction based on ECG signals and multi-network stacking model. 2021 40th Chinese Control Conference (CCC); 2021 26-28 July 2021.
.141 Omar N, Dey M, Ullah MA, editors. Detection of Myocardial Infarction from ECG Signal Through Combining CNN and Bi-LSTM. 2020 11th International Conference on Electrical and Computer Engineering (ICECE); 2020 17-19 Dec. 2020.
.142 Shahnawaz MB, Dawood H. An Effective Deep Learning Model for Automated Detection of Myocardial Infarction Based on Ultrashort-Term Heart Rate Variability Analysis. 2021;2021(1):6455053.
.143 Uchiyama R, Okada Y, Kakizaki R, Tomioka S. End-to-End Convolutional Neural Network Model to Detect and Localize Myocardial Infarction Using 12-Lead ECG Images without Preprocessing. Bioengineering. 2022;9(9):430.
.144 Alnaggar M, Handosa M, Medhat T, Rashad MZJIJoACS, Applications. An IoT-based Framework for Detecting Heart Conditions using Machine Learning. 2023.
.145 Acharya UR, Sudarshan VK, Koh JEW, Martis RJ, Tan JH, Oh SL, et al. Application of higher-order spectra for the characterization of Coronary artery disease using electrocardiogram signals. Biomedical Signal Processing and Control. 2017;31:31-43.
.146 Ribeiro AH, Horta Ribeiro M, Paixão GMM, Oliveira DMd, Gomes PR, Canazart JA, et al. Automatic diagnosis of the 12-lead ECG using a deep neural network. 2019;11.

.147 Brisk R, Bond RR, Finlay DD, McLaughlin JAD, Piadlo A, McEneaney DJJFiP. WaSP-ECG: A Wave Segmentation Pretraining Toolkit for Electrocardiogram Analysis. 2022;13.
.148 Strodthoff N, Strodthoff C. Detecting and interpreting myocardial infarction using fully convolutional neural networks. Physiological Measurement. 2019;40(1):015001.
.149 MI-OPTNET: AN OPTIMIZED DEEP LEARNING FRAMEWORK FOR MYOCARDIAL INFARCTION DETECTION. Jurnal Teknologi (Sciences & Engineering). 2024;86(3):115-25.
.150 Jahmunah V, Ng EYK, San TR, Acharya UR. Automated detection of coronary artery disease, myocardial infarction and congestive heart failure using GaborCNN model with ECG signals. Computers in Biology and Medicine. 2021;134:104457.
.151 Kumar R, Aggarwal Y ,Kumar Nigam V. Heart rate dynamics in the prediction of coronary artery disease and myocardial infarction using artificial neural network and support vector machine. Journal of Applied Biomedicine. 2022;20(2):70-9.
.152 Pan W, An Y, Guan Y, Wang J. MCA-net: A multi-task channel attention network for Myocardial infarction detection and location using 12-lead ECGs. Computers in Biology and Medicine. 2022;150:106199.
.153 Sugimoto K, Kon Y, Lee S, Okada Y. Detection and localization of myocardial infarction based on a convolutional autoencoder. Knowledge-Based Systems. 2019;178:123-31.
.154 Sharma M, Tan RS, Acharya UR. A novel automated diagnostic system for classification of myocardial infarction ECG signals using an optimal biorthogonal filter bank. Computers in Biology and Medicine. 2018;102:341-56.
.155 Kim D, Hwang JE, Cho Y, Cho H-W, Lee W, Lee JH, et al. A Retrospective Clinical Evaluation of an Artificial Intelligence Screening Method for Early Detection of STEMI in the Emergency Department. jkms.:(10)37;2022 e81-0.
.156 Choi HY, Kim W, Kang GH, Jang YS, Lee Y, Kim JG, et al. Diagnostic Accuracy of the Deep Learning Model for the Detection of ST Elevation Myocardial Infarction on Electrocardiogram. Journal of Personalized Medicine. 2022;12(3):336.
.157 Choi SH, Lee H-G, Park S-D, Bae J-W, Lee W, Kim M-S, et al. Electrocardiogram-based deep learning algorithm for the screening of obstructive coronary artery disease. BMC Cardiovascular Disorders. 2023;23(1):287.
.158 Lee S-H, Jeon KL, Lee Y-J, You SC, Lee S-J, Hong S-J, et al. Development of Clinically Validated Artificial Intelligence Model for Detecting ST-segment Elevation Myocardial Infarction. Annals of Emergency Medicine. 2024;84(5):540-8.
.159 Han C, Song Y, Lim H-S, Tae Y, Jang J-H, Lee BT, et al. Automated Detection of Acute Myocardial Infarction Using Asynchronous Electrocardiogram Signals—Preview of Implementing Artificial Intelligence With Multichannel Electrocardiographs Obtained From Smartwatches: Retrospective Study. J Med Internet Res. 2021;23(9):e31129.
.160 Gustafsson S, Gedon D, Lampa E, Ribeiro AH, Holzmann MJ, Schön TB, et al. Development and validation of deep learning ECG-based prediction of myocardial infarction in emergency department patients. Scientific Reports. 2022;12(1).19615:
.161 Jahmunah V, Ng EYK, Tan R, Oh SL, Acharya URJCib, medicine. Explainable detection of myocardial infarction using deep learning models with Grad-CAM technique on ECG signals. 2022;146:105550.
.162 Kavak S, Chiu XD, Yen SJ, Chen MYC. Application of CNN for Detection and Localization of STEMI Using 12-Lead ECG Images. IEEE Access. 2022;10:38923-30.

.163 Tseng LM, Chuang CY, Chua SK, Tseng VS. Identification of Coronary Culprit Lesion in ST Elevation Myocardial Infarction by Using Deep Learning. IEEE Journal of Translational Engineering in Health and Medicine. 2023;11:70-9.
.164 Lin C, Liu W-T, Chang C-H, Lee C-C, Hsing S-C, Fang W-H, et al. Artificial Intelligence–Powered Rapid Identification of ST-Elevation Myocardial Infarction via Electrocardiogram (ARISE) — A Pragmatic Randomized Controlled Trial. NEJM AI. 2024;1(7):AIoa2400190.
.165 Jian J-Z, Ger T-R, Lai H-H, Ku C-M, Chen C-A, Abu PAR, et al. Detection of Myocardial Infarction Using ECG and Multi-Scale Feature Concatenate. 2021;21(5):1906.
.166 Chumachenko D, Butkevych M, Lode D, Frohme M, Schmailzl KJG, Nechyporenko A. Machine Learning Methods in Predicting Patients with Suspected Myocardial Infarction Based on Short-Time HRV Data. Sensors. 2022;22(18):7033.
.167 Ibrahim L, Mesinovic M, Yang KW, Eid MA. Explainable Prediction of Acute Myocardial Infarction Using Machine Learning and Shapley Values. IEEE Access. 2020;8:210410-7.
.168 Garvey JL, Zegre-Hemsey J, Gregg R, Studnek JR. Electrocardiographic diagnosis of ST segment elevation myocardial infarction: An evaluation of three automated interpretation algorithms. Journal of Electrocardiology. 2016;49(5):728-32.
.169 Rashid N, Faruque MAA, editors. Energy-efficient Real-time Myocardial Infarction Detection on Wearable Devices. 2020 42nd Annual International Conference of the IEEE Engineering in Medicine & Biology Society (EMBC); 2020 20-24 July 2020.
.170 Al-Zaiti S, Besomi L, Bouzid Z, Faramand Z, Frisch S, Martin-Gill C, et al. Machine learning-based prediction of acute coronary syndrome using only the pre-hospital 12-lead electrocardiogram. Nature Communications. 2020;11(1):3966.
.171 Panchal HB, Zheng S, White CJ, Leinaar E, Mukherjee D, Mamas MA, et al. TCT CONNECT-41 Impact of Chronic Kidney Disease on Coronary Revascularization and In-Hospital Outcomes in Patients With Acute ST-Segment Elevation Myocardial Infarction. 2020;76.
.172 Sharma LD, Sunkaria RK. Myocardial Infarction Detection and Localization Using Optimal Features Based Lead Specific Approach. IRBM. 2020;41(1):58-70.
.173 Zhang W, Li R, Shen S, Yao J, Peng Y, Chen G, et al. Interpretable Detection and Location of Myocardial Infarction Based on Ventricular Fusion Rule Features. 2021;2021.
.174 Bouzid Z, Faramand Z, Martin-Gill C, Sereika SM, Callaway CW, Saba S, et al. Incorporation of Serial 12-Lead Electrocardiogram With Machine Learning to Augment the Out-of-Hospital Diagnosis of Non-ST Elevation Acute Coronary Syndrome. Annals of Emergency Medicine. 2023;81(1):57-69.
.175 McLaren JTT, Meyers HP, Smith SW. Kenichi Harumi Plenary Address at Annual Meeting of the International Society of Computers in Electrocardiology: "What Should ECG Deep Learning Focus on? The diagnosis of acute coronary occlusion!". Journal of Electrocardiology. 2023;76:39-44.
.176 Xiao R, Ding C, Hu X, Clifford GD, Wright DW, Shah AJ, et al. Integrating multimodal information in machine learning for classifying acute myocardial infarction. Physiological Measurement. 2023;44(4):044002.
.177 Yao Q, Zhang L, Zheng W, Zhou Y, Xiao Y. Multi-scale SE-residual network with transformer encoder for myocardial infarction classification. Applied Soft Computing. 2023;149:110919.

.178 Karnewar JS, Shandilya VJRJoP, Technology. Myocardial Infarction Detection using Morphological Features of ECG Signal. 2023.
.179 Gül E, Diker A, Avcı E, Doğantekin A. MI-CSBO: a hybrid system for myocardial infarction classification using deep learning and Bayesian optimization. Computer Methods in Biomechanics and Biomedical Engineering.1-10.
.180 Kim Y, Jo H, Jang TG, Park SY, Park HY, Cho SP, et al. SleepMI: An AI-based screening algorithm for myocardial infarction using nocturnal electrocardiography. Heliyon. 2024;10(4).
.181 Ma L, Zhang F. A Novel Real-Time Detection and Classification Method for ECG Signal Images Based on Deep Learning. 2024;24(16):5087.
.182 Yildirim O, Baloglu UB, Talo M, Ganesan P, Tung JS, Kang G, et al., editors. Deep Neural Network Trained on Surface ECG Improves Diagnostic Accuracy of Prior Myocardial Infarction Over Q Wave Analysis. 2021 Computing in Cardiology (CinC); 2021 13-15 Sept. 2021.
.183 Seenivasagam V, Chitra RJNNW. Myocardial infarction detection using intelligent algorithms. 2016;26:91-110.
.184 Holst H, Ohlsson M, Peterson C, Edenbrandt LJCp. A confident decision support system for interpreting electrocardiograms. 1999;19 5:410-8.
.185 Odema M, Rashid N, Faruque MAA. Energy-Aware Design Methodology for Myocardial Infarction Detection on Low-Power Wearable Devices. Proceedings of the 26th Asia and South Pacific Design Automation Conference; Tokyo, Japan: Association for Computing Machinery; 2021. p. 621–6.
.186 Litjens G, Ciompi F, Wolterink Jelmer M, de Vos Bob D, Leiner T, Teuwen J, et al. State-of-the-Art Deep Learning in Cardiovascular Image Analysis. JACC: Cardiovascular Imaging. 12;2019(8_Part_1):1549-65.
.187 Liu W, Zhang M, Zhang Y, Liao Y, Huang Q, Chang S, et al. Real-Time Multilead Convolutional Neural Network for Myocardial Infarction Detection. IEEE Journal of Biomedical and Health Informatics. 2018;22(5):1434-44.
.188 Mirza AH, Nurmaini S, Partan RU. Automatic Classification of 15 Leads ECG Signal of Myocardial Infarction Using One Dimension Convolutional Neural Network. 2022;12(11):5603.
.189 Liu W, Wang F, Huang Q, Chang S, Wang H, He J. MFB-CBRNN: A Hybrid Network for MI Detection Using 12-Lead ECGs. IEEE Journal of Biomedical and Health Informatics. 2020;24(2):503-14.
.190 Sinha N, Das A. Identification and Localization of Myocardial Infarction Based on Analysis of ECG Signal in Cross Spectral Domain Using Boosted SVM Classifier. IEEE Transactions on Instrumentation and Measurement. 2021;70:1-9.
.191 Jafarian K, Vahdat V, Salehi S, Mobin M. Automating detection and localization of myocardial infarction using shallow and end-to-end deep neural networks. Applied Soft Computing. 2020;93:106383.
.192 Martin H, Izquierdo W, Cabrerizo M, Cabrera A, Adjouadi M. Near real-time single-beat myocardial infarction detection from single-lead electrocardiogram using Long Short-Term Memory Neural Network. Biomedical Signal Processing and Control. 2021;68:102683.
.193 Muminov B, Nasimov R, Mirzahalilov S, Sayfullaeva N, Gadoyboyeva N, editors. Localization and Classification of Myocardial Infarction Based on Artificial Neural Network. 2020 Information Communication Technologies Conference (ICTC); 2020 29-31 May 2020.

.194 Yousuf A, Hafiz R, Riaz S, Farooq M, Riaz K, Rahman MMU. Inferior Myocardial Infarction Detection From Lead II of ECG: A Gramian Angular Field-Based 2D-CNN Approach. IEEE Sensors Letters. 2024;8(10):1-4.
.195 Zhang J, Lin F, Xiong P, Du H, Zhang H, Liu M, et al. Automated Detection and Localization of Myocardial Infarction With Staked Sparse Autoencoder and TreeBagger. IEEE Access. 2019;7:70634-42.
.196 Anwar SMS, Pal D, Mukhopadhyay S, Gupta R. A Lightweight Method of Myocardial Infarction Detection and Localization From Single Lead ECG Features Using Machine Learning Approach. IEEE Sensors Letters. 2024;8(4):1-4.
.197 Bhaskarpandit S, Gade A, Dash S, Dash DK, Tripathy RK, Pachori RB. Detection of Myocardial Infarction From 12-Lead ECG Trace Images Using Eigendomain Deep Representation Learning. IEEE Transactions on Instrumentation and Measurement. 2023;72:1-12.
.198 Chang K-C, Hsieh P-H, Wu M-Y, Wang Y-C, Wei J-T, Shih ESC, et al. Usefulness of multi-labelling artificial intelligence in detecting rhythm disorders and acute ST-elevation myocardial infarction on 12-lead electrocardiogram. European Heart Journal - Digital Health. 2021;2(2):299-310.
.199 Chen K-W, Wang Y-C, Liu M-H, Tsai B-Y, Wu M-Y, Hsieh P-H, et al. Artificial intelligence-assisted remote detection of ST-elevation myocardial infarction using a mini-12-lead electrocardiogram device in prehospital ambulance care. Frontiers in Cardiovascular Medicine. 2022;Volume 9 - 2022.
.200 Dey M, Omar N, Ullah MA. Temporal Feature-Based Classification Into Myocardial Infarction and Other CVDs Merging CNN and Bi-LSTM From ECG Signal. IEEE Sensors Journal. 2021;21(19):21688-95.
.201 Fajar R, Hanoman B, Zuova R. MACHINE LEARNING CLASSIFICATION OF CORONARY HEART SYNDROME BASED ON ELECTROCARDIOGRAM (ECG) IMAGE USING CONVOLUTIONAL NEURAL NETWORK (CNN) ALGORITHM. Journal of Hypertension. 2022;40(Suppl 1):e77-e8.
.202 Herman R, Meyers HP, Smith SW, Bertolone DT, Leone A, Bermpeis K, et al., editors. INTERNATIONAL EVALUATION OF AN ARTIFICIAL INTELLIGENCE-POWERED ECG MODEL DETECTING OCCLUSION MYOCARDIAL INFARCTION. medRxiv; 2023.
.203 Jahmunah V, Ng EYK, Tan R-S, Oh SL, Acharya UR. Uncertainty quantification in DenseNet model using myocardial infarction ECG signals. Computer Methods and Programs in Biomedicine. 2023;229:107308.
.204 Jiang M, Bian F, Zhang J, Huang T, Xia L, Chu Y, et al. Myocardial infarction detection method based on the continuous T-wave area feature and multi-lead-fusion deep features. Physiological Measurement. 2024;45(5):055017.
.205 Kolliyil Jibin J, Brindise M. AUTONOMOUS DETECTION OF MYOCARDIAL INFARCTION AND OTHER ECG ABNORMALITIES USING PHYSIOLOGICALLY INTERPRETABLE FEATURES. JACC. 2024;83(13_Supplement):83-.
.206 Liang C, Sun Q, Li J, Ji B, Wu W, Zhang F, et al. An interpretable ensemble trees method with joint analysis of static and dynamic features for myocardial infarction detection. Physiological Measurement. 2024;45(8):085006.
.207 Mehta S, Fernandez F, Villagrán C, Niklitschek S, Ávila J, Botelho R, et al. SINGLE LEAD FOR STEMI DETECTION DOES NOT LOCALIZE OR IDENTIFY CULPRIT LESION. JACC. 2020;75(11_Supplement_1):3624-.

.208 Mehta S, Fernandez F, Villagran C, Vieira D, Pinto G, Pisana L, et al. TCT CONNECT-42 Artificial Intelligence Single Lead ECG as a Blueprint for Symptom-to-Balloon Time Reduction. JACC. 2020;76(17 Supplement S):B18-B.
.209 Mehta S, Fernandez F, Villagran C, Vieira D, Pinto G, Quintero S, et al. TCT CONNECT-19 Base Camp to Everest Summit: Rapid Ascent of Artificial Intelligence Algorithms for ST-Elevation Myocardial Infarction Diagnosis. JACC. 2020;76(17 Supplement S):B9-B.
.210 Mishra V. AUTOMATED MYOCARDIAL INFARCTION DETECTION IN 12-LEAD ELECTROCARDIOGRAMS USING DEEP CONVOLUTIONAL NEURAL NETWORKS. JACC. 2024;83(13_Supplement):2597-.
.211 Zhang, Sciences LJA. Application of Heartbeat-Attention Mechanism for Detection of Myocardial Infarction Using 12-Lead ECG Records. 2019.
.212 Padhy S, Dandapat S. Third-order tensor based analysis of multilead ECG for classification of myocardial infarction. Biomedical Signal Processing and Control. 2017;31:71-8.
.213 Karim SR, Helseth HC, Baker PO, Keller GA, Meyers HP, Herman R, et al. Artificial Intelligence Detection of Occlusive Myocardial Infarction from Electrocardiograms Interpreted as “Normal” by Conventional Algorithms. 2025;15(4):130.
.214 Fu L, Lu B, Nie B, Peng Z, Liu H, Pi X. Hybrid Network with Attention Mechanism for Detection and Location of Myocardial Infarction Based on 12-Lead Electrocardiogram Signals. 2020;20(4).1020:
.215 Sbrollini A, Marcantoni I, Morettini M, Swenne CA, Burattini L, editors. Repeated Structuring & Learning Procedure for Detection of Myocardial Ischemia: a Robustness Analysis. 2021 43rd Annual International Conference of the IEEE Engineering in Medicine & Biology Society (EMBC); 2021 1-5 Nov. 2021.
.216 Shin TG, Lee Y, Kim K, Lee MS, Kwon JM. ROMIAE (Rule-Out Acute Myocardial Infarction Using Artificial Intelligence Electrocardiogram Analysis) trial study protocol: a prospective multicenter observational study for validation of a deep learning-based 12-lead electrocardiogram analysis model for detecting acute myocardial infarction in patients visiting the emergency department. Clin Exp Emerg Med. 2023;10(4):438-45.
.217 Tadesse GA, Javed H, Weldemariam K, Liu Y, Liu J, Chen J, et al. DeepMI: Deep multi-lead ECG fusion for identifying myocardial infarction and its occurrence-time. Artificial Intelligence in Medicine. 2021;121:102192.
.218 Wu S, Cao Q, Chen Q, Jin Q, Liu Z, Zhuang L, et al. Using Multi-Task Learning-Based Framework to Detect ST-Segment and J-Point Deviation From Holter. Frontiers in Physiology. 2022;Volume 13 - 2022.
.219 Chen Y, Ye J, Li Y, Luo Z, Luo J, Wan X. A Multi-Domain Feature Fusion CNN for Myocardial Infarction Detection and Localization. 2025;15(6):392.
.220 Peace AJJJoE. The role of the ECG in the primary PCI pathway: A clinical perspective. 2022.
